# Whole transcriptome analyses of six thoroughbred horses before and after exercise using RNA-Seq

**DOI:** 10.1186/1471-2164-13-473

**Published:** 2012-09-12

**Authors:** Kyung-Do Park, Jongsun Park, Junsu Ko, Byung Chul Kim, Heui-Soo Kim, Kung Ahn, Kyoung-Tag Do, Hansol Choi, Hak-Min Kim, Sanghoon Song, Sunghoon Lee, Sungwoong Jho, Hong-Sik Kong, Young Mok Yang, Byung-Hak Jhun, Chulhong Kim, Tae-Hyung Kim, Seungwoo Hwang, Jong Bhak, Hak-Kyo Lee, Byung-Wook Cho

**Affiliations:** 1 Department of Biotechnology, Hankyong National University, Anseong, 456-749, Republic of Korea; 2 Personal Genomics Institute, Genome Research Foundation, 443-270, Suwon, Republic of Korea; 3 Theragen BiO Institute, TheragenEtex, 443-270, Suwon, Republic of Korea; 4 Department of Biological Sciences, College of Natural Sciences, Pusan National University, Busan, 609-735, Republic of Korea; 5 Department of Animal Science, College of Life Sciences, Pusan National University, Miryang, 627-702, Republic of Korea; 6 Department of Pathology, School of Medicine, and Institute of Biomedical Science and Technology, Konkuk University, Seoul, 143-701, Republic of Korea; 7 Department of Nanomedical Engineering, College of Nanoscience and Nanotechnology, Pusan National University, Miryang, 627-702, Republic of Korea; 8 Korean Bioinformation Center, Korea Research Institute of Bioscience and Biotechnology, 305-806, Daejeon, Republic of Korea

**Keywords:** *Transcriptome*, *Equus caballus*, Gene expression, Racing performance

## Abstract

**Background:**

Thoroughbred horses are the most expensive domestic animals, and their running ability and knowledge about their muscle-related diseases are important in animal genetics. While the horse reference genome is available, there has been no large-scale functional annotation of the genome using expressed genes derived from transcriptomes.

**Results:**

We present a large-scale analysis of whole transcriptome data. We sequenced the whole mRNA from the blood and muscle tissues of six thoroughbred horses before and after exercise. By comparing current genome annotations, we identified 32,361 unigene clusters spanning 51.83 Mb that contained 11,933 (36.87%) annotated genes. More than 60% (20,428) of the unigene clusters did not match any current equine gene model. We also identified 189,973 single nucleotide variations (SNVs) from the sequences aligned against the horse reference genome. Most SNVs (171,558 SNVs; 90.31%) were novel when compared with over 1.1 million equine SNPs from two SNP databases. Using differential expression analysis, we further identified a number of exercise-regulated genes: 62 up-regulated and 80 down-regulated genes in the blood, and 878 up-regulated and 285 down-regulated genes in the muscle. Six of 28 previously-known exercise-related genes were over-expressed in the muscle after exercise. Among the differentially expressed genes, there were 91 transcription factor-encoding genes, which included 56 functionally unknown transcription factor candidates that are probably associated with an early regulatory exercise mechanism. In addition, we found interesting RNA expression patterns where different alternative splicing forms of the same gene showed reversed expressions before and after exercising.

**Conclusion:**

The first sequencing-based horse transcriptome data, extensive analyses results, deferentially expressed genes before and after exercise, and candidate genes that are related to the exercise are provided in this study.

## Background

The thoroughbred, a “hot-blooded” horse breed, is the favorite breed for use in horse racing [1]. The speed and agility of thoroughbred horses has resulted in the emergence of an industry involved in the breeding, training, and racing of elite racehorses worth many billions of dollars [2]. Until now, relatively few genes related to their athletic phenotypes have been identified, even though physical and physiological adaptations underlying their elite athleticism are well characterized [3]. Muscle is the most critical tissue for athletic performance. The skeletal muscle of the thoroughbred horse comprises over 55% of its total body mass [4,5] and has remarkable functional and structural plasticity [3]. Furthermore, over 90 hereditary conditions in horses have corresponding human disorders [3,6], and many muscle disorders in humans and horses share common clinical and histopathological characteristics, as well as molecular features [7-9]. Therefore, the horse can be an invaluable animal model for muscle diseases.

An international team of researchers has decoded the genome of the domestic horse, *Equus caballus*, and has reported that its genome structure is remarkably similar to the human genome [8]. An additional nine domesticated horse breeds have also been sequenced, identifying around one million single nucleotide polymorphisms (SNPs) [8]. However, there has been little progress in refining the functional annotation of horse genome using expressed genes. Although a small number (around 30,000) of expressed sequence tags (ESTs) has been deposited in the dbEST [10], this is insufficient to identify all the key genes related to specific functions, such as racing performance.

RNA-Seq is one of the most useful next generation sequencing (NGS) methods used to fully understand the landscape of a transcriptome, because it produces several tens of millions of short reads (17 bp to 101 bp) from the expressed genes *in vivo*. RNA-Seq has been used successfully to investigate the transcriptome profiles of human, mouse, Arabidopsis, and yeast [11-14]. The RNA-Seq data generally exhibit a high degree of concordance with established gene annotations [15,16]. Using RNA-Seq, researchers have identified numerous novel genes and additional alternative splicing forms [14,17,18], as well as unraveling expression profiles underlying phenotypic changes, such as development stages [19,20]. In addition, RNA-Seq permits the identification of single nucleotide variations (SNVs) in coding regions from various organisms because of the large number of reads [14,21,22]. Moreover, RNA-Seq has identified novel unannotated transcriptionally active regions in rice [23], indicating that there are novel genes that cannot be detected by conventional gene prediction methods.

In horses, two transcriptome studies using RNA-Seq have been reported: one study refined the structural annotation of protein-coding genes based on the RNA-Seq sequences from several equine tissues [24], while the other analyzed RNA-Seq sequences acquired from skeletal muscle to find long-term training-related genes [25]. Both studies used very short RNA-Seq sequences, 17 bp and 35 bp, respectively, because of the limitation of NGS technologies available at that time. These very short-read RNA sequences have one critical limitation. When aligned against the reference genome, the typical success rate is as low as 66% in the case of 17 bp RNA fragments [24]. This was caused not only by the very short-read sequences, but also by intron junctions, which were not included in the short-reads. This disadvantage must be overcome to advance horse transcriptome research.

Here, we present a large scale analysis of whole transcriptome data. The samples were taken from blood and muscle tissues of six thoroughbred horses before and after 30 minutes of exercises, resulting in 24 samples.

## Results and discussion

### Gene cluster analysis and identification of novel transcripts from the horse RNA-Seq sequences

**Figure 1:**
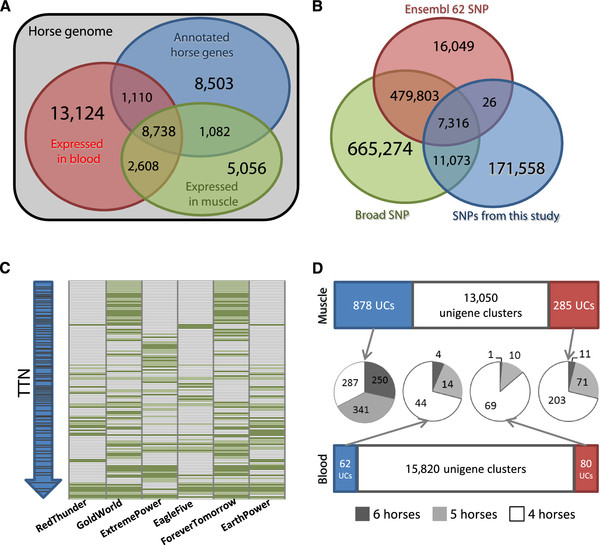
**Enhanced genome annotation, single nucleotide variation analyses, and differentially expressed genes before and after exercise in horse.** (**A**) Red and green circles indicate expressed genes in the blood and muscle tissues, respectively, and the blue circle shows the current Ensembl annotation (Release 62). The grey rectangle indicates the coverage of the current horse genome. (**B**) Green circle: SNPs provided by the Broad Institute, red circle: SNPs provided by Ensembl (release 62), blue circle: SNPs identified from this study. (**C**) SNV profiles of six horses for the titin (*TTN*) gene. The top of the blue arrow is the 5' end and the bottom is 3' end of *TTN* gene. The X-axis shows the names of the horses. Dark green horizontal bars are non-synonymous SNVs. Light green horizontal bars are synonymous SNVs. (**D**) Blue bars: >2-fold upregulated genes, red bars: >2-fold downregulated genes, white bars: not differentially expressed. The four pie charts display the composition of the DEGs supported by four horses (white), five horses (light grey), and six horses (grey).

To construct high quality horse transcriptome data, we generated over 1.3 billion 90-bp pair-end reads using an Illumina HiSeq2000 (Additional file 1: Figure S1, Additional file 1: Table S1, and Table S2). Using TopHat [26] and Cufflinks [27], 84.60% of all the reads were successfully mapped against the current horse reference genome (Additional file 1: Table S3). A novel bioinformatics protocol for processing large amounts of transcriptome sequences was built (Additional file 1: Figure S2). RNA sequences were obtained from 24 different samples; therefore, we defined a new concept, unigene cluster (UC), which contains overlapped unigene sequences originating from multiple samples. Utilizing the current annotation (Ensembl 62), 32,361 unigene clusters (UCs), with a total length of 51.83 Mb, were identified. 11,933 UCs matched current gene models, which comprised 36.87% of the 32,361 UCs (Figure 1A and Additional file 1: Supplementary Methods) [8]. The remaining 20,428 UCs (63.13%), which contained more than 60% of the transcripts, were novel (Additional file 1: Supplementary Methods and Additional file 1: Figure S3). The expressions of eight randomly selected novel UCs were confirmed by reverse transcription PCR (Additional file 1: Figure S4 and Additional file 1: Table S4). In addition, the unmapped raw sequences were processed by SOAPdenovo [28], resulting in assemblies of 42,476 to 72,011 scaffolds for each sample. These assembled sequences increased the extent of the current horse genome (Additional file 1: Supplementary Methods, Additional file 1: Figure S2, and Additional file 1: Tables S5, S6, S7, S8). When we pooled the scaffolds together, we identified around 670,000 non-redundant unigenes. 27% to 46% of these unigenes from each sample were matched to human genes using tBLASTx (Additional file 1: Table S9). 

### Identification and dissection of novel SNVs from a large amount of horse RNA-Seq sequences

We identified 182,722 non-redundant SNPs and 7,251 non-redundant INDELs from the 24 samples using several filters, including an exon-intron boundary misalignment filter (Additional file 1: Figure S5, Additional file 1: Tables S10, S11, S12, and Additional file 1: Supplementary Methods). The filters had been validated on mouse RNA-Seq data [29] by showing that 80.28% of the identified SNPs were confirmed in two inbred mouse genomes (Additional file 1: Supplementary Methods and Additional file 1: Table S13). Each horse showed a similar number of SNVs, ranging from 72,000 to 77,000 (Additional file 1: Table S11). 82,476 individual-specific SNVs were identified (Additional file 1: Table S12). Only 7,316 out of 182,722 SNPs (4.00%) overlapped with both of the two existing databases (Figure 1B). This is because only 1% (10,229 from Broad Institute and 4,287 from Ensembl) of the SNPs from the two databases were located in the exonic region of the current genome annotation (Additional file 1: Tables S11 and S14). These results demonstrate the usefulness of identifying novel SNPs from transcriptome data. Moreover, 116,650 (61.40%) of 189,973 SNVs were located in exons of unigene clusters, and 67,788 (58.11%) of the 116,650 SNVs caused amino acid changes. Some of the transcripts are possibly related to the horses’ running ability. For example, titin (*TTN*) is related to the passive stiffness of muscle by limiting the range of motion of the sarcomere in tension [30], such that *TTN* affects the ability of muscle directly [31]. Other examples include obscurin (*OBSCN*), which is associated with *TTN* and *ANK1* genes [32,33], and the skeletal muscle calcium release channel gene (*RYR1*) located in the sarcoplasmic reticulum [34,35], whose mutations caused several muscle-related diseases, including central core disease [36]. The SNV distribution profile of the *TTN* was specific to individual horses (Figure 1C and Additional file 1: Table S15).

### Comparison of the expressed genes in blood and muscle tissues with other organisms

In muscle and blood tissues, 17,484 and 25,220 UCs were identified as expressed genes, respectively, showing that blood expressed about 40% more genes than muscle tissue (Figure 1A). By comparison with two previous RNA-Seq studies conducted in human (Illumina BodyMap2 transcriptome) and in mouse skeletal muscle tissue [12], we observed that the GO classifications of all the expressed genes in the three organisms were similar to each other (Additional file 1: Figure S6). The differences in GO assignments between tissues (muscle and blood) were larger than those of the same tissue among the three species (Additional file 1: Figure S7).

### Identification and characterization of differentially expressed genes in blood and muscle tissues regulated by exercise

We calculated the expression level of all unigenes from the 24 samples. The distribution of 32,361 UCs’ average expression levels in the 24 samples showed that half of the UCs were expressed at less than 0.19 FPKM (fragments per kilobase of exon model per million mapped reads) and three-quarters of the UCs were expressed at less than 4.81 FPKM (Additional file 1: Figure S10). The correlation coefficients of the FPKM values among the samples were comparable to previous RNA-Seq studies [15,37] (Additional file 1: Figures S8 and S9). By comparing before and after 30 minutes of exercise, we identified 1,285 differentially expressed genes (DEGs), consisting of 62 up- and 80 downregulated UCs in blood and 878 up- and 285 downregulated UCs in muscle (Additional file 1: Supplementary Methods and Figure 1D). While the overall number of all the differentially expressed UCs was much larger in muscle than in blood, the number of novel differentially expressed UCs was larger in blood (42 UCs) than in muscle (8 UCs) (Additional file 1: Table S16).

We examined 28 genes that are known to be associated with racing performance in horses [38]. Twelve of the 25 genes successfully mapped on the genome annotation were expressed in muscle and blood, and six were differentially expressed in muscle (Additional file 1: Table S17). The rest were detected in neither of the two tissues. The six genes upregulated by exercise were: *HIF1A*, which encodes a transcription factor that responds to hypoxia*; ADRB2*, which is involved in the regulation of energy expenditure and lipid mobilization from adipose; *PPARD*, which regulates expression of genes involved in lipid and carbohydrate metabolisms; *VEGF*, which is an important angiogenic factor recovering the oxygen supply to tissues when blood vessels are blocked; *TNC*, which is located in positive-selected regions for racing performance [4]; and *BDNF*, which is a candidate gene that may be associated with exercise behavior [39].

We also compared differentially expressed genes (DEGs) in muscle tissue with the 15 upregulated and 53 downregulated DEGs that are associated with exercise training [25] (Additional file 1: Table S18). Among these 68 DEGs, only five genes, ACTR3B, FBXO32, PER3, C1orf51, and GATM, were identified as DEGs in this study, among which C1orf51 and PER3 showed a different expression profile.

Sampling the transcriptomes immediately after exercise enabled the identification of differentially expressed early response genes that are rapidly induced by exercise. Many early response proteins include transcriptional regulators, such as Mitogen-activated protein kinases (MAPKs) and NF-κB, which promote fuel homeostasis and prevent skeletal muscle atrophy [40]. In addition, important oxidative stress-sensitive enzymes that can be activated by NF-κB and MAPKs after exercise, such as inducible nitric oxide synthase (iNOS; ENSECAT00000026843) [41], were upregulated in horse muscle after exercise. Among the 1,285 DEGs from the two tissues, we identified 91 transcription factors, which might regulate downstream components of exercise-triggered signaling pathways (Additional file 1: Table S19). *GATA2*, which can interact with AP1 transcription factors to regulate MAPK and NF-κB signaling [42], was underexpressed in blood, while *CREB5*, whose zinc-finger and bZIP domain can specifically bind to the CRE with c-Jun or CRE-BP1 [43], was overexpressed in muscle. Upregulation of *CREB5* might be explained by the fact that *CREB5* and c-Jun genes are involved in calcium-dependent transcriptional pathways in skeletal muscle [44].

At least 56 uncharacterized transcription factors could be candidates for novel primary transcriptional regulators accompanying exercise. We validated the expression levels of seven randomly chosen transcription factors using quantitative RT-PCR from the remaining sample materials (Additional file 1: Tables S20 and S21).

### Switching the expression pattern of alternative splicing forms of the gene before and after exercising

**Figure 2:**
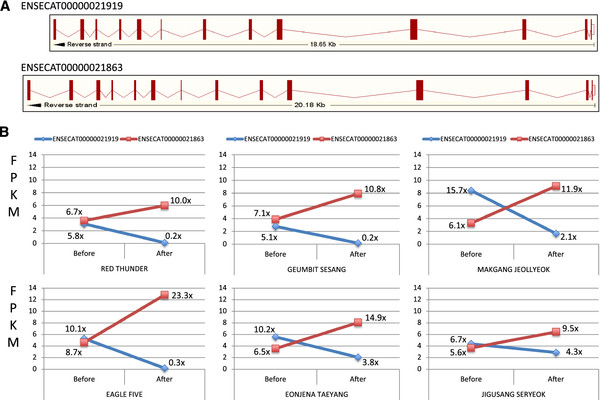
**Switching expressions of alternatively spliced forms before and after exercise.** (**A**) Red bars are the exons of the two transcripts of the *DYNC1* gene: ENSECAT00000021919 and ENSECAT00000021863. (**B**) Each plot shows the gene expression level (FPKM value; fragments per kilobase of exon per million fragments mapped) of the two transcripts (Blue lines represent the ENSECAT00000021919 transcript and red lines represent the ENSECAT00000021863 transcript) in each individual horse, whose name is shown as the plot title. Percentages inside the plots are the coverages of the transcripts.

Four genes (three from muscle, one from blood) showed interesting RNA expression patterns, in which two different alternative splicing forms of the same gene showed reversed expression patterns before and after exercising, similar to that of the *SXL* gene in Drosophila [45]. This observation suggested a cost-effective method of regulation: the cells do not have to produce completely new exons and proteins, but merely change the composition of the existing exons [46]. The genes with reversed expression are: *AXL*, *DYNC1*, *PLEKHG1*, and *COBLL1* (Additional file 1: Table S22 and Additional file 1: Figure S11). Figure 2 shows cytoplasmic dynein intermediate chain (ENSECAG00000020218) protein (DYNC1) [47] as an example of the reversed expression pattern in muscle before and after exercising. 

## Conclusion

We generated a large amount of horse transcriptome data. Their analyses provided candidate genes that are related to horse racing performance: six previously identified exercise-associated genes and 91 early regulated transcription factors that are differentially expressed by exercise, three genes that display high SNV density, and four alternatively expressed splicing variants. In addition, all 1,258 differentially expressed genes could be important candidate genes for further research.

## Methods

The muscle and blood samples from six retired thoroughbred horses were taken before and after trotting (30 minutes). 90-bp pair-end sequences were obtained with an Illumina HiSeq2000, San Diego, US, from the samples. These sequences were mapped against the horse reference genome (Ensembl release 62) using TopHat 1.2.0 with two options (−−mate-inner-dist = 200 and --allow-indels) for paired-end sequences and identified unigenes using the Cufflinks program without genome annotation data. From the results of 24 samples, novel genes were clustered based on the genomic coordination to define unigene clusters (UCs). The generated UCs were subjected to the filter, which extracted UCs overlapping with the genome annotation. The expressed genes annotated by the pipeline and the filtered UCs were merged as the final set of UCs. In addition, unmapped sequences were assembled by the SOAPdenovo [28] program and were subjected to an ORF length filtering process. In-house bioinformatics pipelines were used to identify SNVs under several stringent conditions, especially for the exon-intron boundary misalignment filter (See Additional file 1: Supplementary Methods). Differentially expressed genes were selected by comparing the expression profiles of the six horses, with a selection criterion of more than two fold up- or downregulation in four or more horses.

## Data access

All raw sequences of the horse transcriptomes are openfreely available at http://www.horsegenome.net/.

## Competing interests

The authors declare that they have no competing interests.

## Authors’ contributions

KDP, THK, SL, HKL, BWC, and JB designed and supervised the experiments and analyses. KDP, JP, BCK, HKL, BWC, and JB supervised the progress of the project. KDP, BWC, KTD, YMY, HSK, CK, and THK prepared blood and muscle samples from the six horses. CK, BCK, and THK generated sequences from the samples. JP, HC, HMK, SS, and SJ conducted the bioinformatics analyses. HSK, JP, and JB designed the validation experiments, and KA and KTD conducted the experiments. JP, BCK, SL, SH, JK, and JB wrote the manuscript and BCK, HC, SL, SH, and YMY participated in improving the manuscript. All authors read and approved the final manuscript.

## Supplementary Material

Additional file 1 **Figure S1.** Six thoroughbred horses. **Table S1.** 24 sample names from six thoroughbred horses used in this study. **Table S2.** The statistics of RNA-Seq raw data from 24 different samples. **Table S3.** The mapping results against the horse reference genome (Ensembl 62). **Figure S2.** Procedure for identifying horse unigenes. **Figure S3.** Distribution plot of the exons identified without gene models which contain ORFs. **Figure S4.** Reverse transcript PCR (RT-PCR) confirmation of the 8 novel unigene clusters (UCs) **Table S4.** Primer information for the RT-PCR experiment. **Table S5.** Statistics of the filtered *de novo* transcripts identified from cufflink. **Table S6.***de novo* assembly results from one sample with various k-mer values. **Table S7.***de novo* assembly of unmapped sequences originated from 24 samples. **Table S8.** Filtered and clustered unigenes from the scaffolds assembled from unmatched sequences. **Figure S5.** The whole process of identifying SNVs. **Table S9.** The proportion of the scaffolds from the unmapped sequences which were matched against the human genome. **Table S10.** The statistics of total SNVs identified from 24 samples. **Table S11.** The number of total SNVs identified in thoroughbred horses. **Table S12.** The number of individual-specific SNVs. **Table S13.** Conformation of the SNPs identified from the mouse sample [2]. **Table S14.** Distribution of SNP locations in three datasets. **Table S15.** The list of transcripts which have ten or more non-synonymous SNPs. **Figure S6.** GO classification of all expressed genes in human, mouse, and horse muscle tissue. **Figure S7.** GO classification of all expressed horse genes in blood and muscle tissue. **Figure S8.** Correlation matrix of the 24 samples. **Figure S9.** Correlation matrix of three human samples from kidney and liver tissues. **Figure S10.** Histogram of average expression level of the unigene clusters in the 24 samples. **Table S16.** List of DEGs in muscle and blood tissues. **Table S17.** Expression profiles of known exercise-related horse genes. **Table S18.** Comparison between DEGs in muscle tissue and the DEGs which are responsible to exercise training [25]. **Table S19.** List of transcription factors differentially expressed in muscle and blood tissues. **Table S20.** RT-PCR primers for seven transcription factors. **Table S21.** RT-PCR results of differentially expressed transcription factors in muscle tissue. **Table S22.** The list of four genes of which alternative splicing forms showed reversed expression patterns before and after exercising. **Figure S11.** Expression profiles of the genes of which alternative splicing forms showed reversed expression patterns before and after exercising. **Table S23.** Number of filtered *de novo* transcripts identified by **Cufflink. Supplementary methods.** Click here for file
