# Supplementary material for: Whole transcriptome analyses of six thoroughbred horses before and after exercise using RNA-Seq
Source: BMC Genomics. 2012 Sep 12;13:473. doi: 10.1186/1471-2164-13-473 (PMC3472166; doi:10.1186/1471-2164-13-473)
Supplement: Additional file 1 — Figure S1. Six thoroughbred horses. Table S1. 24 sample names from six thoroughbred horses used in this study. Table S2. The statistics of RNA-Seq raw data from 24 different samples. Table S3. The mapping results against the horse reference genome (Ensembl 62). Figure S2. Procedure for identifying horse unigenes. Figure S3. Distribution plot of the exons identified without gene models which contain ORFs. Figure S4. Reverse transcript PCR (RT-PCR) confirmation of the 8 novel unigene clusters (UCs) Table S4. Primer information for the RT-PCR experiment. Table S5. Statistics of the filtered de novo transcripts identified from cufflink. Table S6.de novo assembly results from one sample with various k-mer values. Table S7.de novo assembly of unmapped sequences originated from 24 samples. Table S8. Filtered and clustered unigenes from the scaffolds assembled from unmatched sequences. Figure S5. The whole process of identifying SNVs. Table S9. The proportion of the scaffolds from the unmapped sequences which were matched against the human genome. Table S10. The statistics of total SNVs identified from 24 samples. Table S11. The number of total SNVs identified in thoroughbred horses. Table S12. The number of individual-specific SNVs. Table S13. Conformation of the SNPs identified from the mouse sample [2]. Table S14. Distribution of SNP locations in three datasets. Table S15. The list of transcripts which have ten or more non-synonymous SNPs. Figure S6. GO classification of all expressed genes in human, mouse, and horse muscle tissue. Figure S7. GO classification of all expressed horse genes in blood and muscle tissue. Figure S8. Correlation matrix of the 24 samples. Figure S9. Correlation matrix of three human samples from kidney and liver tissues. Figure S10. Histogram of average expression level of the unigene clusters in the 24 samples. Table S16. List of DEGs in muscle and blood tissues. Table S17. Expression profiles of known exercise-related horse genes. Table S18. C [file 1471-2164-13-473-S1.doc]

**Supplementary Figure S1. Six thoroughbred horses.**

**
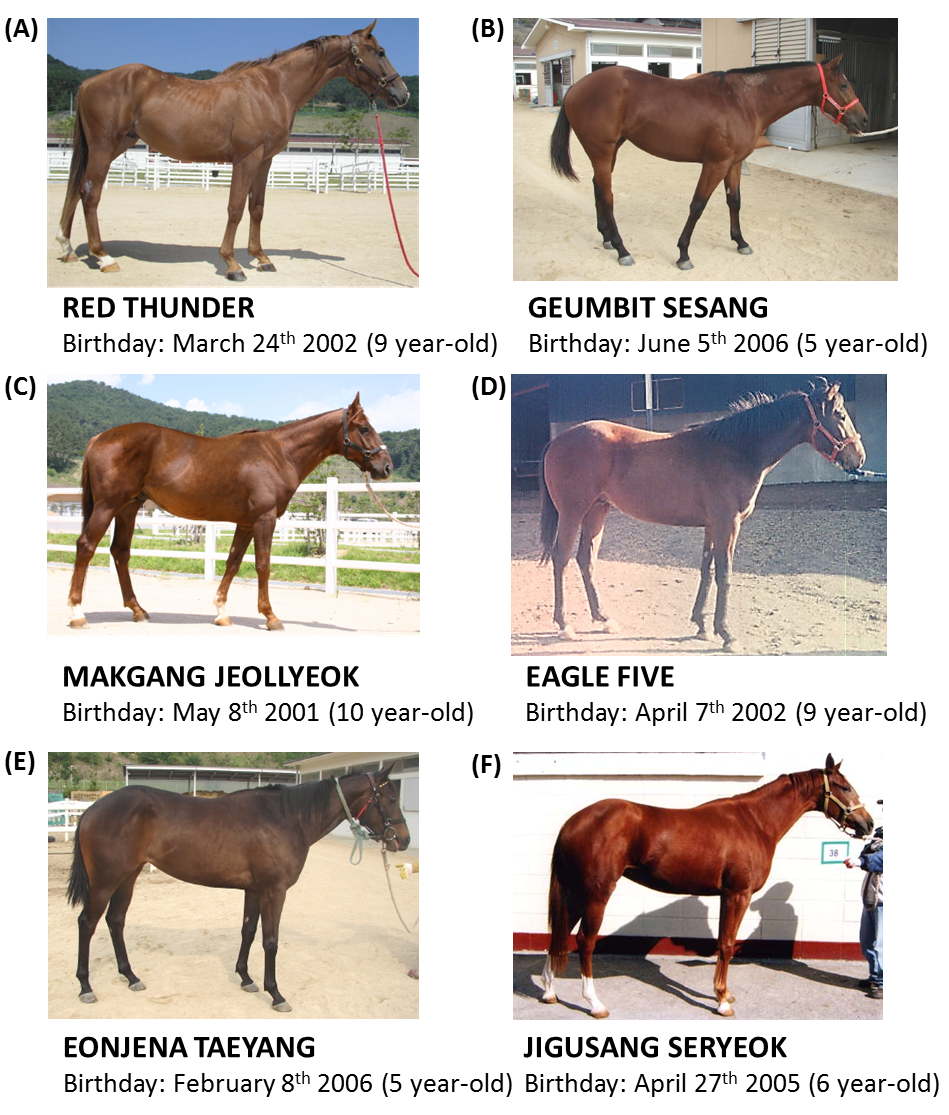
**

**Supplementary Table S1. 24 sample names from six thoroughbred horses used in this study.**

| **Tissues** | **Horse name** | **Before exercise** | **After exercise** |
| --- | --- | --- | --- |
| **Muscle** | RED THUNDER | MF1B | MF1P |
|  | GEUMBIT SESANG | MF2B | MF2P |
|  | MAKGANG JEOLLYEOK | MF3B | MF3P |
|  | EAGLE FIVE | MS1B | MS1P |
|  | EONJENA TAEYANG | MS2B | MS2P |
|  | JIGUSANG SERYEOK | MS3B | MS3P |
| **Blood** | RED THUNDER | BF1B | BF1P |
|  | GEUMBIT SESANG | BF2B | BF2P |
|  | MAKGANG JEOLLYEOK | BF3B | BF3P |
|  | EAGLE FIVE | BS1B | BS1P |
|  | EONJENA TAEYANG | BS2B | BS2P |
|  | JIGUSANG SERYEOK | BS3B | BS3P |

**Supplementary Table S2. The statistics of RNA-Seq raw data from 24 different samples.**

| **ID** | **# of reads** | **Total length (nt)** | **Q20** | **N bases** | **GC ratio** |
| --- | --- | --- | --- | --- | --- |
| MF1B | 57,906,674 | 5,211,600,660 | 90.29% | 0.00% | 50.35% |
| MF1P | 56,437,124 | 5,079,341,160 | 92.21% | 0.00% | 50.64% |
| MF2B | 57,212,080 | 5,149,087,200 | 91.70% | 0.00% | 51.72% |
| MF2P | 57,906,674 | 5,211,600,660 | 92.22% | 0.00% | 48.29% |
| MF3B | 57,906,674 | 5,211,600,660 | 93.37% | 0.01% | 49.36% |
| MF3P | 57,190,010 | 5,147,100,900 | 90.06% | 0.01% | 47.94% |
| MS1B | 57,906,674 | 5,211,600,660 | 89.65% | 0.01% | 49.57% |
| MS1P | 57,906,674 | 5,211,600,660 | 92.16% | 0.00% | 49.29% |
| MS2B | 57,906,674 | 5,211,600,660 | 93.03% | 0.01% | 50.05% |
| MS2P | 56,138,892 | 5,052,500,280 | 93.05% | 0.01% | 49.37% |
| MS3B | 56,138,892 | 5,052,500,280 | 92.12% | 0.00% | 48.92% |
| MS3P | 56,138,892 | 5,052,500,280 | 93.45% | 0.01% | 47.72% |
| BF1B | 53,155,558 | 4,784,000,220 | 93.95% | 0.00% | 50.85% |
| BF1P | 52,222,222 | 4,699,999,980 | 93.25% | 0.00% | 50.83% |
| BF2B | 52,222,222 | 4,699,999,980 | 93.61% | 0.00% | 51.28% |
| BF2P | 52,222,222 | 4,699,999,980 | 93.79% | 0.00% | 50.52% |
| BF3B | 52,222,222 | 4,699,999,980 | 93.26% | 0.00% | 51.57% |
| BF3P | 53,155,558 | 4,784,000,220 | 94.67% | 0.01% | 50.14% |
| BS1B | 52,222,222 | 4,699,999,980 | 93.57% | 0.00% | 50.64% |
| BS1P | 51,244,448 | 4,612,000,320 | 95.34% | 0.01% | 50.64% |
| BS2B | 52,022,226 | 4,682,000,340 | 95.20% | 0.01% | 51.20% |
| BS2P | 50,015,638 | 4,501,407,420 | 94.62% | 0.01% | 51.32% |
| BS3B | 51,666,600 | 4,649,994,000 | 95.31% | 0.00% | 50.96% |
| BS3P | 52,266,672 | 4,704,000,480 | 95.05% | 0.00% | 51.65% |
| **Total** | **1,311,333,744** | **118,020,036,960** | **93.06%** | **0.01%** | **50.17%** |

**Supplementary Table S3. The mapping results against the horse reference genome (Ensembl 62).**

| **ID** | **# of reads** | **Mapped reads** | **Unmapped reads (both pairs)** | **Unmapped reads (single pair)** | **Mapping ratio** |
| --- | --- | --- | --- | --- | --- |
| MF1B | 57,906,672 | 49,141,985 | 3,088,276 | 5,676,411 | 84.86% |
| MF1P | 56,437,122 | 48,282,317 | 2,958,178 | 5,196,627 | 85.55% |
| MF2B | 57,212,080 | 48,125,939 | 3,104,274 | 5,981,867 | 84.12% |
| MF2P | 57,906,672 | 51,362,530 | 2,255,886 | 4,288,256 | 88.70% |
| MF3B | 57,906,672 | 49,830,724 | 2,605,680 | 5,470,268 | 86.05% |
| MF3P | 57,190,008 | 51,211,208 | 1,910,560 | 4,068,240 | 89.55% |
| MS1B | 57,906,672 | 49,043,120 | 3,220,488 | 5,643,064 | 84.69% |
| MS1P | 57,906,672 | 49,748,165 | 2,927,222 | 5,231,285 | 85.91% |
| MS2B | 57,906,672 | 49,152,226 | 2,894,074 | 5,860,372 | 84.88% |
| MS2P | 56,138,890 | 48,100,882 | 2,696,528 | 5,341,480 | 85.68% |
| MS3B | 56,138,890 | 48,970,763 | 2,457,826 | 4,710,301 | 87.23% |
| MS3P | 56,138,890 | 49,464,883 | 2,229,928 | 4,444,079 | 88.11% |
| BF1B | 53,155,556 | 44,396,061 | 3,514,986 | 5,244,509 | 83.52% |
| BF1P | 52,222,220 | 43,662,727 | 3,475,008 | 5,084,485 | 83.61% |
| BF2B | 52,222,220 | 43,754,824 | 3,391,738 | 5,075,658 | 83.79% |
| BF2P | 52,222,220 | 43,873,930 | 3,380,832 | 4,967,458 | 84.01% |
| BF3B | 52,222,220 | 43,416,635 | 3,569,232 | 5,236,353 | 83.14% |
| BF3P | 53,155,556 | 44,514,469 | 3,533,228 | 5,107,859 | 83.74% |
| BS1B | 52,222,220 | 43,758,629 | 3,458,934 | 5,004,657 | 83.79% |
| BS1P | 51,244,444 | 42,968,497 | 3,420,608 | 4,855,339 | 83.85% |
| BS2B | 52,022,222 | 43,226,680 | 3,575,576 | 5,219,966 | 83.09% |
| BS2P | 50,015,638 | 37,605,265 | 7,623,916 | 4,786,457 | 75.19% |
| BS3B | 51,666,598 | 42,915,032 | 3,523,066 | 5,228,500 | 83.06% |
| BS3P | 52,266,668 | 42,894,229 | 3,985,796 | 5,386,643 | 82.07% |
| **Total** | **1,311,333,694** | **1,109,421,720** | **78,801,840** | **123,110,134** | **84.60%** |

**Supplementary Figure S2. Procedure for identifying horse unigenes.**

**
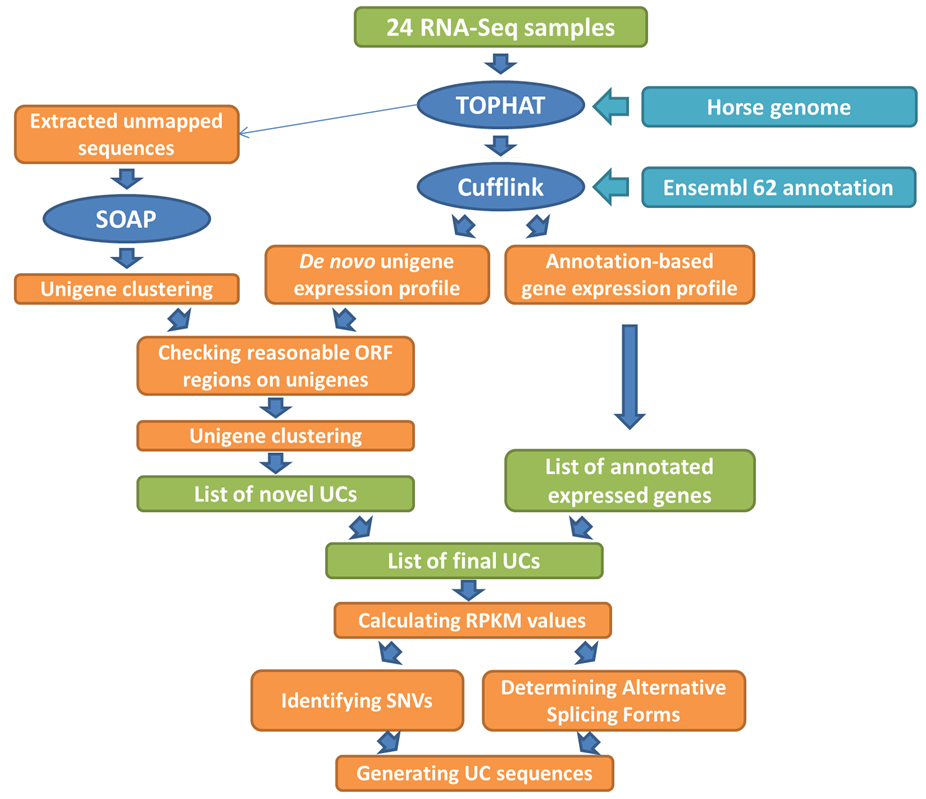
**

Orange boxes are processes, and green boxes are intermediate results. Blue circles are the programs used in this procedure, and light-blue boxes are external data utilized.

**Supplementary Figure S3. Distribution plot of the exons identified without gene models which contain ORFs.**

**
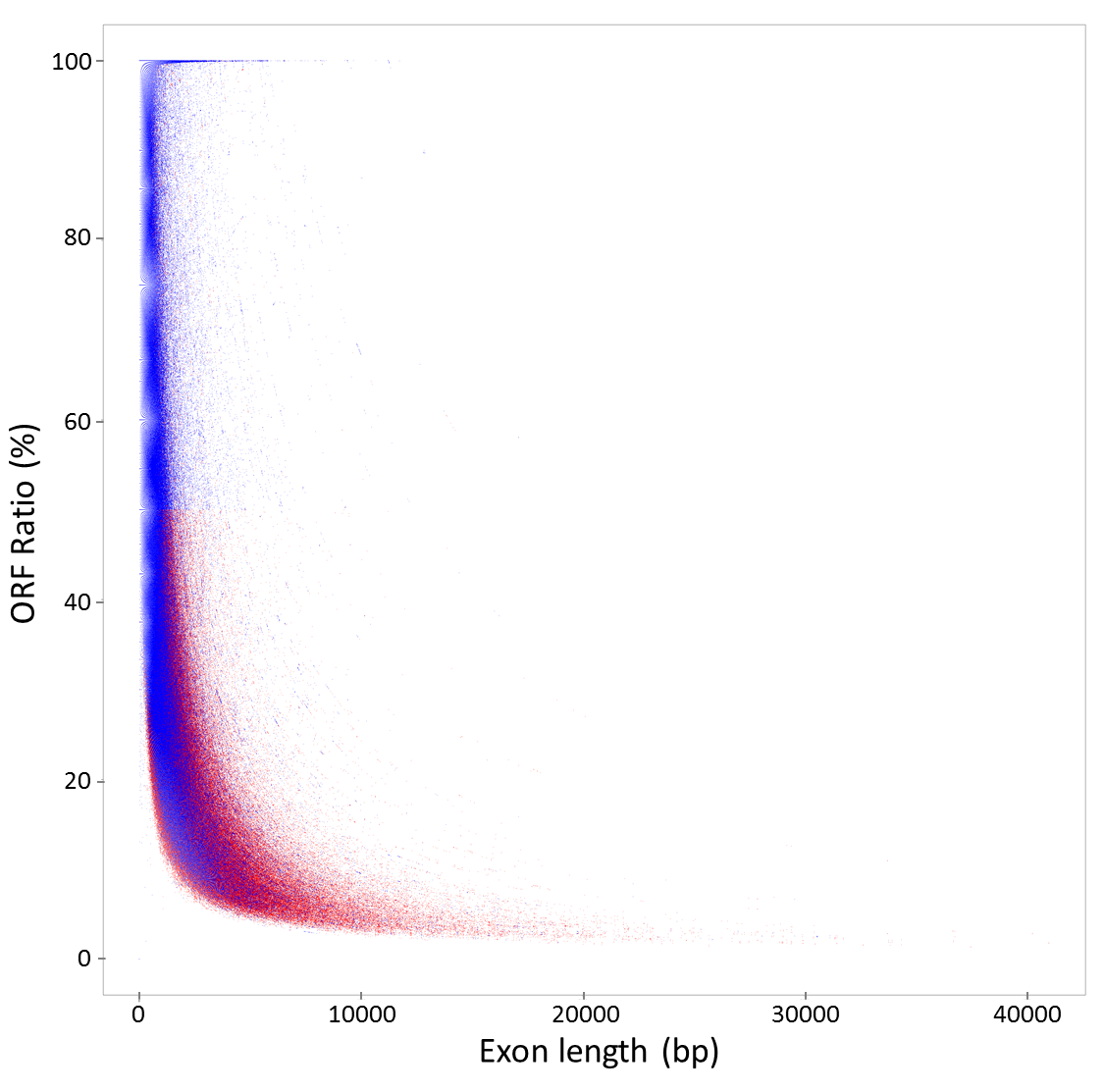
**

X-axis is exon length, and Y-axis is the percentage of the translated regions based on the exon length. Each dot represents the exons predicted by the Cufflink program without the gene model. Blue dots were selected because they contained long enough ORFs, while red dots were excluded due to short translated regions.

**Supplementary Figure S4. Reverse transcript PCR (RT-PCR) confirmation of the 8 novel unigene clusters (UCs)**

**
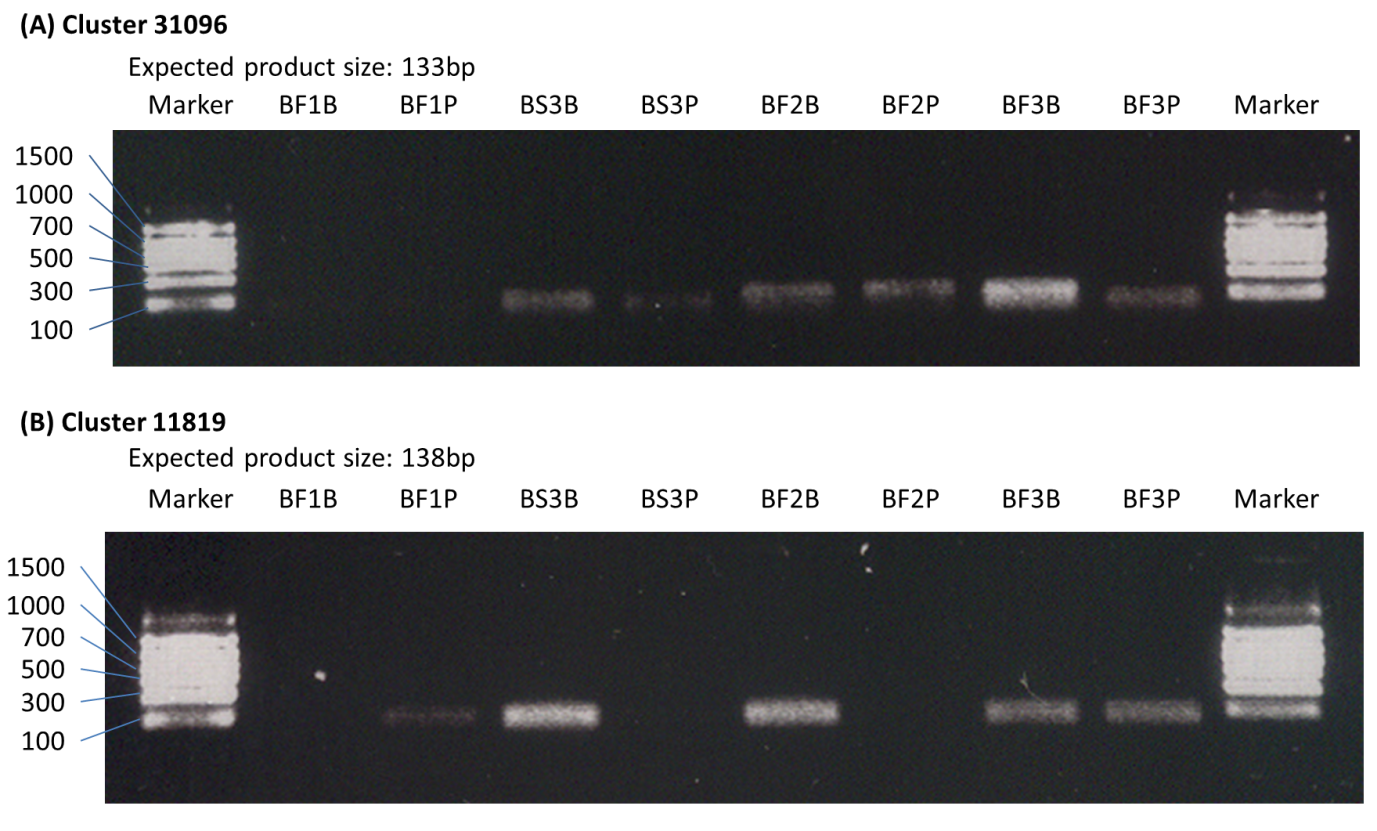
**

**
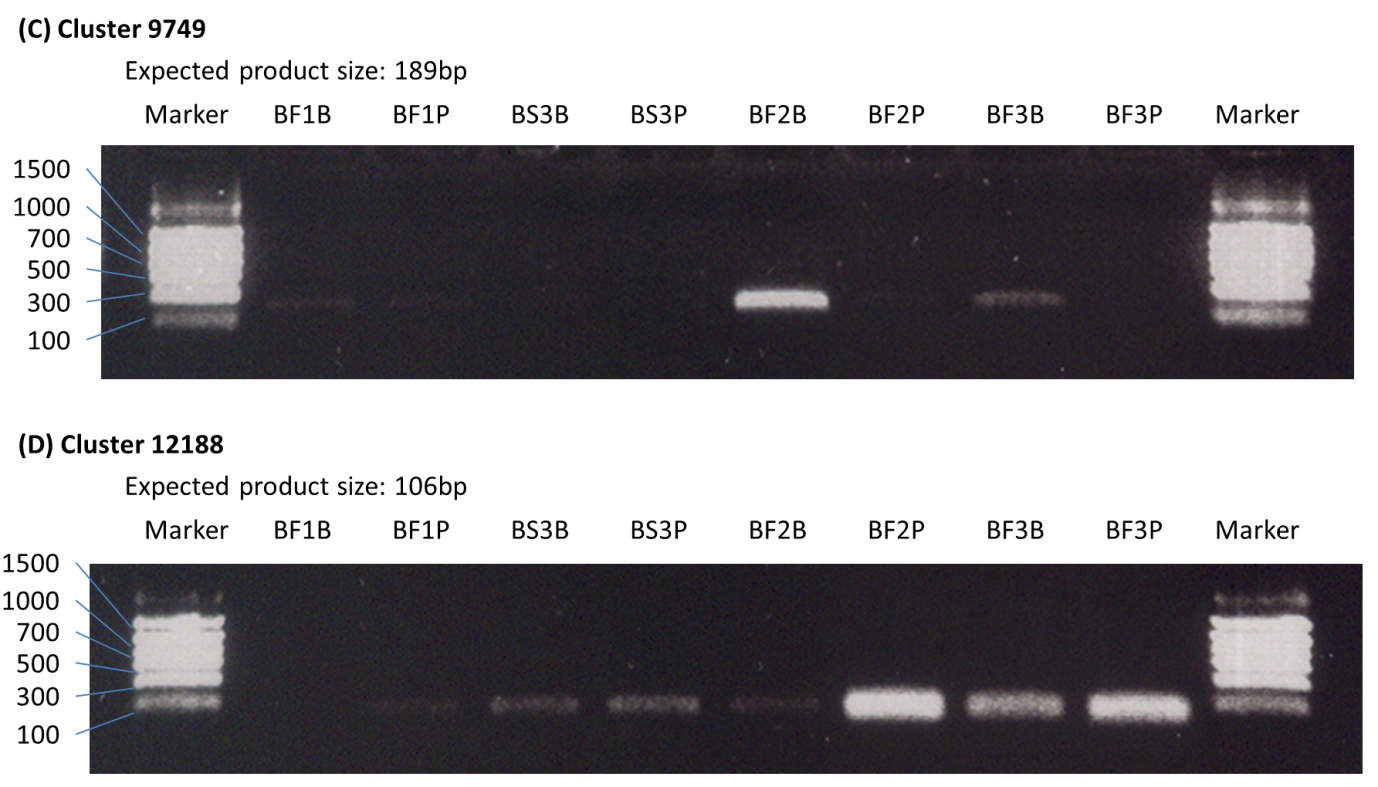
**

**
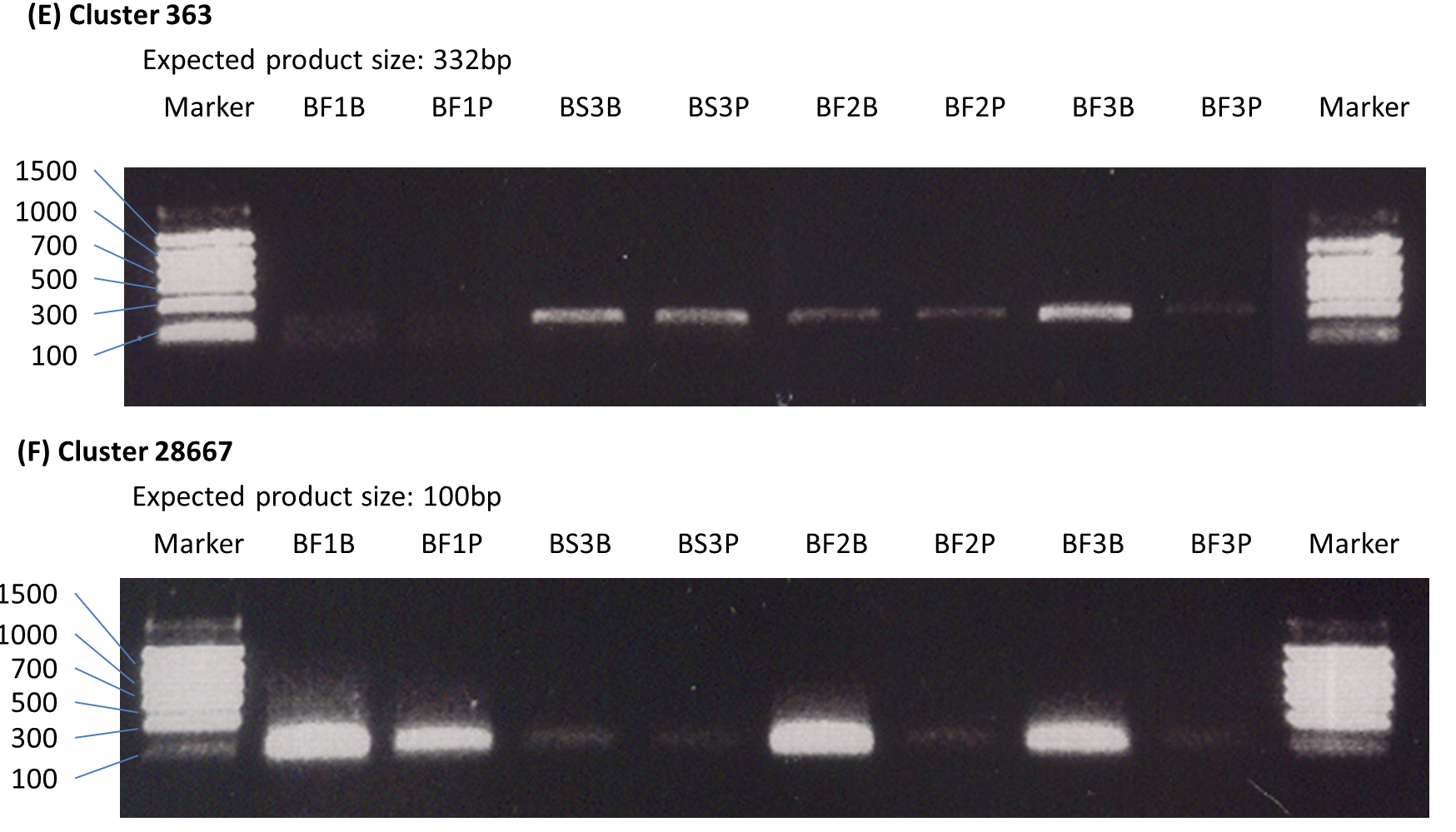
**

**Supplementary Table S4. Primer information for the RT-PCR experiment**

| **Unigene cluster** | **Primer sequence** | | **Product size (bp)** | **TM(℃)** |
| --- | --- | --- | --- | --- |
|  | **(5')-Forward primer-(3')** | **(5')-Reverse primer-(3')** |  |  |
| Cluster 31096 | TGTTAGCGGGAAGCTCCTTA | CCCAAGAGCCCTCCTAAATC | 133 | 53 |
| Cluster 11819 | ATCCCGCACTTCTAGGTTGA | CATTTCCTTTTCTCCCCCA | 138 | 53 |
| Cluster 9749 | AGTTGATGGACCAATCAGGC | TCACCTGGGGCTACAGAATC | 189 | 55 |
| Cluster 12188 | TCAAATTTCCAAGCTTTCGC | TGGCAAAGCAGATTAGCATAC | 106 | 53 |
| Cluster 21909 | CTGTGCCTTTCCCAGGTCTA | GGCCAACTGTACAGGGAAGT | 101 | 53 |
| Cluster 28667 | ATTCTTGGCAGAAGGCAAAA | TTGGGAACATTGTTTGACCC | 100 | 55 |
| Cluster 23092 | AGTTTTCACGGCAGACTCCA | CAAAACCCAAAGGCTACAGG | 398 | 55 |
| Cluster 363 | GACCTTTTGATCTTGGCGTT | ATTGAAGTGTGGTTCAGGGC | 332 | 55 |

**Supplementary Table S5. Statistics of the filtered *de novo* transcripts identified from cufflink**.

| **ID** | **# of *de novo* transcripts** | **# of filtered *de novo* transcripts** | **Filtered Ratio** |
| --- | --- | --- | --- |
| MF1B | 51,030 | 30,411 | 59.59% |
| MF1P | 48,366 | 28,962 | 59.88% |
| MF2B | 46,299 | 25,463 | 55.00% |
| MF2P | 44,490 | 25,225 | 56.70% |
| MF3B | 45,411 | 26,227 | 57.75% |
| MF3P | 39,451 | 21,183 | 53.69% |
| MS1B | 51,246 | 29,350 | 57.27% |
| MS1P | 49,915 | 28,176 | 56.45% |
| MS2B | 42,757 | 23,816 | 55.70% |
| MS2P | 45,500 | 26,672 | 58.62% |
| MS3B | 39,102 | 21,332 | 54.55% |
| MS3P | 38,384 | 22,213 | 57.87% |
| BF1B | 74,105 | 49,806 | 67.21% |
| BF1P | 77,492 | 51,520 | 66.48% |
| BF2B | 82,305 | 55,347 | 67.25% |
| BF2P | 81,997 | 56,088 | 68.40% |
| BF3B | 74,039 | 50,018 | 67.56% |
| BF3P | 77,407 | 51,917 | 67.07% |
| BS1B | 85,327 | 57,701 | 67.62% |
| BS1P | 80,665 | 55,535 | 68.85% |
| BS2B | 80,439 | 55,249 | 68.68% |
| BS2P | 74,968 | 49,820 | 66.46% |
| BS3B | 77,035 | 52,429 | 68.06% |
| BS3P | 71,390 | 47,943 | 67.16% |
| **Total** | **1,479,120** | **942,403** | **63.71%** |

**Supplementary Table S6. *de novo* assembly results from one sample with various k-mer values**.

| **K-mer** | **17** | **19** | **21** | **23** | **25** | **27** |
| --- | --- | --- | --- | --- | --- | --- |
| **# of scaffolds** | 80,037 | 84,605 | 83,616 | 81,956 | 79,951 | 77,492 |
| **Total length (bp)** | 13,498,263 | 15,509,480 | 15,714,307 | 15,613,862 | 15,382,668 | 15,081,275 |
| **Average length (bp)** | 168.65 | 183.32 | 187.93 | 190.52 | 192.4 | 194.62 |
| **N50 (bp)** | 166 | 186 | 193 | 197 | 201 | 205 |

**Supplementary Table S7. *de novo* assembly of unmapped sequences originated from 24 samples.**

| **ID** | **# of reads** | **# of scaffolds** | **Total length (bp)** | **Average (bp)** | **N50 (bp)** |
| --- | --- | --- | --- | --- | --- |
| MF1B | 8,764,687 | 63,591 | 11,312,319 | 177.89 | 177 |
| MF1P | 8,154,805 | 55,629 | 10,434,135 | 187.57 | 193 |
| MF2B | 9,086,141 | 55,706 | 10,277,208 | 184.49 | 190 |
| MF2P | 6,544,142 | 51,587 | 9,285,450 | 180.00 | 182 |
| MF3B | 8,075,948 | 62,736 | 11,702,703 | 186.54 | 189 |
| MF3P | 5,978,800 | 49,228 | 8,541,446 | 173.51 | 171 |
| MS1B | 8,863,552 | 67,102 | 11,781,203 | 175.57 | 175 |
| MS1P | 8,158,507 | 61,335 | 11,268,211 | 183.72 | 188 |
| MS2B | 8,754,446 | 59,902 | 11,032,080 | 184.17 | 187 |
| MS2P | 8,038,008 | 61,261 | 11,174,275 | 182.40 | 185 |
| MS3B | 7,168,127 | 50,936 | 9,343,873 | 183.44 | 185 |
| MS3P | 6,674,007 | 51,265 | 9,252,129 | 180.48 | 180 |
| BF1B | 8,759,495 | 83,616 | 15,714,307 | 187.93 | 193 |
| BF1P | 8,559,493 | 81,697 | 15,086,921 | 184.67 | 187 |
| BF2B | 8,467,396 | 87,387 | 16,329,210 | 186.86 | 190 |
| BF2P | 8,348,290 | 84,564 | 15,813,206 | 187.00 | 192 |
| BF3B | 8,805,585 | 84,532 | 16,225,111 | 191.94 | 198 |
| BF3P | 8,641,087 | 85,568 | 16,022,717 | 187.25 | 192 |
| BS1B | 8,463,591 | 83,705 | 15,831,604 | 189.14 | 195 |
| BS1P | 8,275,947 | 87,545 | 16,580,924 | 189.40 | 194 |
| BS2B | 8,795,542 | 87,765 | 16,955,107 | 193.19 | 200 |
| BS2P | 12,410,373 | 161,265 | 31,975,301 | 198.28 | 212 |
| BS3B | 8,751,566 | 84,846 | 16,031,907 | 188.95 | 194 |
| BS3P | 9,372,439 | 82,371 | 15,547,123 | 188.75 | 194 |

,

**Supplementary Table S8. Filtered and clustered unigenes from the scaffolds assembled from unmatched sequences.**

| **ID** | **# of scaffolds** | **# of filtered scaffolds** | **Ratio (%)** | **Total length (bp)** | **Average (bp)** | **N50 (bp)** |
| --- | --- | --- | --- | --- | --- | --- |
| MF1B | 63,591 | 53,575 | 84.25% | 9,672,649 | 182.56 | 170 |
| MF1P | 55,629 | 47,577 | 85.53% | 9,051,690 | 189.61 | 182 |
| MF2B | 55,706 | 48,532 | 87.12% | 9,092,908 | 176.36 | 181 |
| MF2P | 51,587 | 44,304 | 85.88% | 8,088,060 | 177.56 | 174 |
| MF3B | 62,736 | 53,639 | 85.50% | 10,170,659 | 186.63 | 182 |
| MF3P | 49,228 | 42,476 | 86.28% | 7,491,021 | 187.30 | 166 |
| MS1B | 67,102 | 56,763 | 84.59% | 10,078,949 | 185.54 | 167 |
| MS1P | 61,335 | 52,480 | 85.56% | 9,794,469 | 186.65 | 179 |
| MS2B | 59,902 | 51,569 | 86.09% | 9,658,721 | 183.84 | 180 |
| MS2P | 61,261 | 52,504 | 85.71% | 9,741,743 | 189.61 | 177 |
| MS3B | 50,936 | 43,890 | 86.17% | 8,191,858 | 186.32 | 178 |
| MS3P | 51,265 | 43,465 | 84.78% | 7,990,684 | 188.42 | 173 |
| BF1B | 83,616 | 68,816 | 82.30% | 13,048,218 | 187.90 | 180 |
| BF1P | 81,697 | 67,056 | 82.08% | 12,494,132 | 182.56 | 175 |
| BF2B | 87,387 | 72,011 | 82.40% | 13,568,465 | 189.61 | 178 |
| BF2P | 84,564 | 69,360 | 82.02% | 13,032,549 | 176.36 | 176 |
| BF3B | 84,532 | 69,715 | 82.47% | 13,430,619 | 192.65 | 184 |
| BF3P | 85,568 | 69,592 | 81.33% | 13,107,990 | 188.35 | 178 |
| BS1B | 83,705 | 68,543 | 81.89% | 13,033,854 | 190.16 | 180 |
| BS1P | 87,545 | 71,259 | 81.40% | 13,571,988 | 190.46 | 179 |
| BS2B | 87,765 | 72,606 | 82.73% | 14,108,755 | 194.32 | 185 |
| BS2P | 161,265 | 126,598 | 78.50% | 24,223,724 | 191.34 | 181 |
| BS3B | 84,846 | 69,998 | 82.50% | 13,320,368 | 190.30 | 179 |
| BS3P | 82,371 | 67,582 | 82.05% | 12,853,279 | 190.19 | 178 |
| **Total** | **1,785,139** | **1,411,304** | **79.06%** | **278,817,352** | **197.56** | **-** |

**Supplementary Figure S5. The whole process of identifying SNVs.**

**
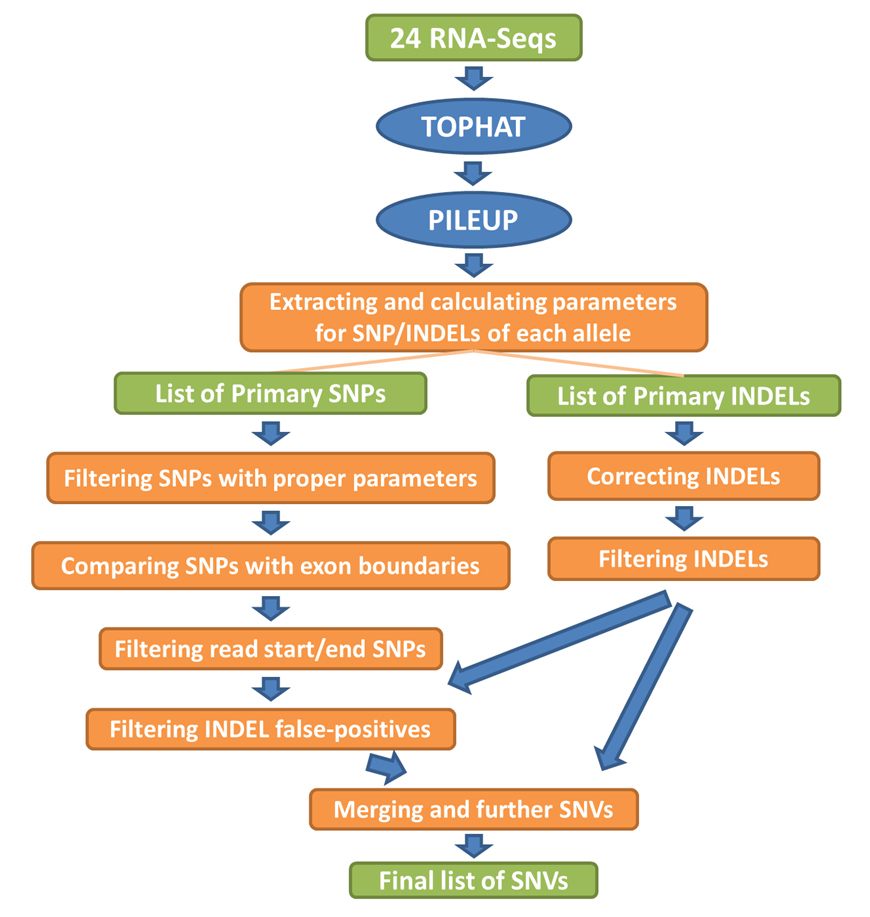
**

**Supplementary Table S9. The proportion of the scaffolds from the unmapped sequences which were matched against the human genome**

| **Samples** | **# of filtered scaffolds** | **# of scaffolds matched to the human genome** | **%** |
| --- | --- | --- | --- |
| BF1B | 68,816 | 28,585 | 41.54% |
| BF1P | 67,056 | 28,637 | 42.71% |
| BF2B | 72,011 | 30,868 | 42.87% |
| BF2P | 69,360 | 29,554 | 42.61% |
| BF3B | 69,715 | 28,691 | 41.15% |
| BF3P | 69,592 | 29,818 | 42.85% |
| BS1B | 68,543 | 28,802 | 42.02% |
| BS1P | 71,259 | 31,215 | 43.80% |
| BS2B | 72,606 | 30,333 | 41.78% |
| BS2P | 126,598 | 59,314 | 46.85% |
| BS3B | 69,998 | 28,149 | 40.21% |
| BS3P | 67,582 | 28,722 | 42.50% |
| MF1B | 53,575 | 18,241 | 34.05% |
| MF1P | 47,577 | 14,989 | 31.50% |
| MF2B | 48,532 | 13,867 | 28.57% |
| MF2P | 44,304 | 13,491 | 30.45% |
| MF3B | 53,639 | 16,652 | 31.04% |
| MF3P | 42,476 | 11,725 | 27.60% |
| MS1B | 56,763 | 20,693 | 36.46% |
| MS1P | 52,480 | 16,935 | 32.27% |
| MS2B | 51,569 | 16,092 | 31.20% |
| MS2P | 52,504 | 16,628 | 31.67% |
| MS3B | 43,890 | 12,662 | 28.85% |
| MS3P | 43,465 | 13,737 | 31.60% |

**Supplementary Table S10**. The statistics of total SNVs identified from 24 samples.

| **Sample** | **# of SNPs** | **# of INDELs** | **Sample** | **# of SNPs** | **# of INDELs** |
| --- | --- | --- | --- | --- | --- |
| **BF1B** | 49,533 | 1,682 | **MF1B** | 23,221 | 808 |
| **BF1P** | 47,247 | 1,599 | **MF1P** | 21,775 | 722 |
| **BF2B** | 50,141 | 1,640 | **MF2B** | 20,358 | 684 |
| **BF2P** | 51,640 | 1,714 | **MF2P** | 18,681 | 650 |
| **BF3B** | 49,207 | 1,591 | **MF3B** | 23,458 | 831 |
| **BF3P** | 49,792 | 1,734 | **MF3P** | 15,090 | 499 |
| **BS1B** | 50,979 | 1,644 | **MS1B** | 21,321 | 775 |
| **BS1P** | 51,060 | 1,616 | **MS1P** | 22,808 | 846 |
| **BS2B** | 50,126 | 1,699 | **MS2B** | 20,554 | 732 |
| **BS2P** | 49,849 | 1,476 | **MS2P** | 21,945 | 795 |
| **BS3B** | 51,604 | 1,687 | **MS3B** | 17,506 | 662 |
| **BS3P** | 45,778 | 1,502 | **MS3P** | 18,619 | 697 |

**Supplementary Table S11**. The number of total SNVs identified in thoroughbred horses.

| **Horse name** | **# of non-redundant SNPs** | **# of INDELs** | **SNV density (per Kb)^a^** |
| --- | --- | --- | --- |
| RED THUNDER | 75,036 | 3,051 | 0.46 |
| GEUMBIT SESANG | 75,878 | 3,015 | 0.46 |
| MAKGANG JEOLLYEOK | 73,238 | 2,970 | 0.45 |
| EAGLE FIVE | 76,894 | 3,015 | 0.47 |
| EONJENA TAEYANG | 76,207 | 2,862 | 0.46 |
| JIGUSANG SERYEOK | 72,509 | 2,920 | 0.44 |
| **Total** | 449,762 | 17,883 | - |

^a^This value was calculated based on the total length of maximized exons (170,539,108 bp)

**Supplementary Table S12. The number of individual-**specific SNVs.

| **Horse name** | **# of SNPs** | **# of INDELs** | **# of individual specific SNPs** | **# of individual specific INDELs** | **Ratio (%)** | |
| --- | --- | --- | --- | --- | --- | --- |
| RED THUNDER | 75,036 | 3,051 | 13,225 | 562 | 17.62 | 18.42 |
| GEUMBIT SESANG | 75,878 | 3,015 | 12,892 | 534 | 16.99 | 17.71 |
| MAKGANG JEOLLYEOK | 73,238 | 2,970 | 11,300 | 512 | 15.43 | 17.24 |
| EAGLE FIVE | 76,894 | 3,015 | 14,132 | 602 | 18.38 | 19.97 |
| EONJENA TAEYANG | 76,207 | 2,862 | 16,086 | 503 | 21.11 | 17.58 |
| JIGUSANG SERYEOK | 72,509 | 2,920 | 11,597 | 531 | 15.99 | 18.18 |
| **Total** | - | - | 79,232 | 3,244 | - | - |

**Supplementary Table S13. Conformation of the SNPs identified from the mouse sample .**

| **RNA-Seq Sample** | **# of SNPs identified by our filter** | **# of SNPs identified from mouse genome sequences** | **# of overlapped SNPs** | **Ratio (%)** |
| --- | --- | --- | --- | --- |
| **C57BL6J x CastEiJ** | 52,832 | 18,186,916 | 42,148 | 79.78 |
| **CastEiJ x C57BL6J** | 55,009 | 18,186,916 | 44,431 | 80.77 |
| **Total** | **107,842** | **-** | **86,579** | **80.28** |

**Supplementary Table S14**. Distribution of SNP locations in three datasets.

| **Type** | **SNP dataset in this study** | **INDEL dataset in this study** | **Ensembl SNP dataset** | **Broad Institute SNP dataset** |
| --- | --- | --- | --- | --- |
| **Exon** | 35,782 | 928 | 4,287 | 10,229 |
| **Intron** | 67,222 | 2,122 | 116,567 | 263,839 |
| **5' UTR** | 3,075 | 111 | 4,518 | 25,021 |
| **3' UTR** | 2,889 | 148 | 3,199 | 10,077 |
| **Intergenic** | 73,754 | 3,942 | 376,528 | 854,300 |
| **Total** | 182,722 | 7,251 | 505,099 | 1,163,466 |

**Supplementary Table S15**. The list of transcripts which have ten or more non-synonymous SNPs.

| **No** | **Protein name** | **Transcript name** | **Genename** | **Ensembl genename** | **# of nsSNVs** |
| --- | --- | --- | --- | --- | --- |
| 1 | ENSECAP00000013718 | ENSECAT00000016948 | TTN | ENSECAG00000012544 | 73 |
| 2 | ENSECAP00000014761 | ENSECAT00000018114 | OBSCN | ENSECAG00000016720 | 57 |
| 3 | ENSECAP00000012698 | ENSECAT00000015763 | OFD1 | ENSECAG00000014736 | 50 |
| 4 | ENSECAP00000011888 | ENSECAT00000014851 | AHNAK | ENSECAG00000014229 | 38 |
| 5 | ENSECAP00000018340 | ENSECAT00000022206 | Q30462_HORSE | ENSECAG00000020358 | 34 |
| 6 | ENSECAP00000005223 | ENSECAT00000007204 | FINC_HORSE | ENSECAG00000000701 | 29 |
| 7 | ENSECAP00000005228 | ENSECAT00000007210 | FINC_HORSE | ENSECAG00000000701 | 29 |
| 8 | ENSECAP00000005229 | ENSECAT00000007212 | FINC_HORSE | ENSECAG00000000701 | 28 |
| 9 | ENSECAP00000014450 | ENSECAT00000017767 | Q0R0C2_HORSE | ENSECAG00000015782 | 25 |
| 10 | ENSECAP00000002837 | ENSECAT00000004108 | XIRP2 | ENSECAG00000002596 | 25 |
| 11 | ENSECAP00000002510 | ENSECAT00000003559 | XIRP2 | ENSECAG00000002596 | 25 |
| 12 | ENSECAP00000020573 | ENSECAT00000024746 | | ENSECAG00000021671 | 24 |
| 13 | ENSECAP00000000451 | ENSECAT00000000577 | | ENSECAG00000000683 | 24 |
| 14 | ENSECAP00000020515 | ENSECAT00000024683 | LOC100054254 | ENSECAG00000022961 | 23 |
| 15 | ENSECAP00000014316 | ENSECAT00000017611 | MACF1 | ENSECAG00000013366 | 23 |
| 16 | ENSECAP00000009219 | ENSECAT00000011750 | LOC100055473 | ENSECAG00000011057 | 23 |
| 17 | ENSECAP00000006893 | ENSECAT00000009107 | RANBP2 | ENSECAG00000008458 | 23 |
| 18 | ENSECAP00000020460 | ENSECAT00000024620 | | ENSECAG00000021671 | 22 |
| 19 | ENSECAP00000016711 | ENSECAT00000020358 | LOC100067361 | ENSECAG00000019130 | 22 |
| 20 | ENSECAP00000014751 | ENSECAT00000018104 | Q30469_HORSE | ENSECAG00000017143 | 22 |
| 21 | ENSECAP00000014763 | ENSECAT00000018116 | Q30469_HORSE | ENSECAG00000017143 | 22 |
| 22 | ENSECAP00000014661 | ENSECAT00000018004 | Q0R0C2_HORSE | ENSECAG00000015782 | 22 |
| 23 | ENSECAP00000007504 | ENSECAT00000009807 | | ENSECAG00000009625 | 22 |
| 24 | ENSECAP00000022720 | ENSECAT00000027155 | MYH3 | ENSECAG00000025060 | 21 |
| 25 | ENSECAP00000006914 | ENSECAT00000009133 | RANBP2 | ENSECAG00000008458 | 21 |
| 26 | ENSECAP00000003552 | ENSECAT00000005076 | LOC100050968 | ENSECAG00000005116 | 21 |
| 27 | ENSECAP00000020377 | ENSECAT00000024524 | | ENSECAG00000021671 | 20 |
| 28 | ENSECAP00000009251 | ENSECAT00000011792 | LOC100055473 | ENSECAG00000011057 | 20 |
| 29 | ENSECAP00000021755 | ENSECAT00000026107 | Q30457_HORSE | ENSECAG00000024259 | 19 |
| 30 | ENSECAP00000019909 | ENSECAT00000023992 | DRB | ENSECAG00000022072 | 19 |
| 31 | ENSECAP00000019115 | ENSECAT00000023093 | LOC100072672 | ENSECAG00000021708 | 19 |
| 32 | ENSECAP00000009517 | ENSECAT00000012088 | MYO18B | ENSECAG00000011440 | 19 |
| 33 | ENSECAP00000009264 | ENSECAT00000011806 | Q38RB9_HORSE | ENSECAG00000009069 | 19 |
| 34 | ENSECAP00000020078 | ENSECAT00000024180 | LOC100058098 | ENSECAG00000021750 | 18 |
| 35 | ENSECAP00000008920 | ENSECAT00000011411 | | ENSECAG00000009368 | 18 |
| 36 | ENSECAP00000008517 | ENSECAT00000010960 | | ENSECAG00000009368 | 18 |
| 37 | ENSECAP00000016196 | ENSECAT00000019763 | TACC2 | ENSECAG00000018280 | 17 |
| 38 | ENSECAP00000010284 | ENSECAT00000013000 | MIA3 | ENSECAG00000012339 | 17 |
| 39 | ENSECAP00000010256 | ENSECAT00000012971 | MIA3 | ENSECAG00000012339 | 17 |
| 40 | ENSECAP00000009859 | ENSECAT00000012478 | BDP1 | ENSECAG00000011402 | 17 |
| 41 | ENSECAP00000008739 | ENSECAT00000011207 | LOC100064396 | ENSECAG00000010232 | 17 |
| 42 | ENSECAP00000004572 | ENSECAT00000006449 | Q9BCX4_HORSE | ENSECAG00000006492 | 17 |
| 43 | ENSECAP00000000911 | ENSECAT00000001158 | LOC100071582 | ENSECAG00000001218 | 17 |
| 44 | ENSECAP00000002356 | ENSECAT00000003319 | Q38RB6_HORSE | ENSECAG00000000325 | 17 |
| 45 | ENSECAP00000020931 | ENSECAT00000025168 | LOC100067561 | ENSECAG00000023475 | 16 |
| 46 | ENSECAP00000018545 | ENSECAT00000022437 | LOC100068911 | ENSECAG00000021110 | 16 |
| 47 | ENSECAP00000014157 | ENSECAT00000017426 | RNF213 | ENSECAG00000016217 | 16 |
| 48 | ENSECAP00000014762 | ENSECAT00000018115 | LOC100147640 | ENSECAG00000015514 | 16 |
| 49 | ENSECAP00000014756 | ENSECAT00000018109 | LOC100147640 | ENSECAG00000015514 | 16 |
| 50 | ENSECAP00000014783 | ENSECAT00000018139 | LOC100147640 | ENSECAG00000015514 | 16 |
| 51 | ENSECAP00000014774 | ENSECAT00000018128 | LOC100147640 | ENSECAG00000015514 | 16 |
| 52 | ENSECAP00000014793 | ENSECAT00000018149 | LOC100147640 | ENSECAG00000015514 | 16 |
| 53 | ENSECAP00000014735 | ENSECAT00000018085 | LOC100147640 | ENSECAG00000015514 | 16 |
| 54 | ENSECAP00000014393 | ENSECAT00000017705 | MACF1 | ENSECAG00000013366 | 16 |
| 55 | ENSECAP00000008796 | ENSECAT00000011275 | LOC100054114 | ENSECAG00000008977 | 16 |
| 56 | ENSECAP00000006541 | ENSECAT00000008698 | | ENSECAG00000008594 | 16 |
| 57 | ENSECAP00000005104 | ENSECAT00000007060 | | ENSECAG00000007049 | 16 |
| 58 | ENSECAP00000023051 | ENSECAT00000028869 | | ENSECAG00000026907 | 15 |
| 59 | ENSECAP00000020651 | ENSECAT00000024835 | PDE4DIP | ENSECAG00000022891 | 15 |
| 60 | ENSECAP00000017726 | ENSECAT00000021519 | LOC100054613 | ENSECAG00000019875 | 15 |
| 61 | ENSECAP00000022805 | ENSECAT00000028872 | LOC100062842 | ENSECAG00000017353 | 15 |
| 62 | ENSECAP00000016175 | ENSECAT00000019739 | LOC100062842 | ENSECAG00000017353 | 15 |
| 63 | ENSECAP00000014908 | ENSECAT00000018287 | TRPS1 | ENSECAG00000017249 | 15 |
| 64 | ENSECAP00000008790 | ENSECAT00000011269 | LOC100064396 | ENSECAG00000010232 | 15 |
| 65 | ENSECAP00000008674 | ENSECAT00000011136 | LOC100064396 | ENSECAG00000010232 | 15 |
| 66 | ENSECAP00000006528 | ENSECAT00000008684 | LOC100059800 | ENSECAG00000007640 | 15 |
| 67 | ENSECAP00000019451 | ENSECAT00000023481 | NCKAP1 | ENSECAG00000021389 | 14 |
| 68 | ENSECAP00000018565 | ENSECAT00000022459 | LOC100068911 | ENSECAG00000021110 | 14 |
| 69 | ENSECAP00000020159 | ENSECAT00000024271 | NEB | ENSECAG00000020638 | 14 |
| 70 | ENSECAP00000012604 | ENSECAT00000015660 | | ENSECAG00000014997 | 14 |
| 71 | ENSECAP00000010867 | ENSECAT00000013673 | Q30463_HORSE | ENSECAG00000012933 | 14 |
| 72 | ENSECAP00000010488 | ENSECAT00000013234 | LOC100053014 | ENSECAG00000011696 | 14 |
| 73 | ENSECAP00000010500 | ENSECAT00000013247 | LOC100053014 | ENSECAG00000011696 | 14 |
| 74 | ENSECAP00000008602 | ENSECAT00000011054 | LOC100064870 | ENSECAG00000010736 | 14 |
| 75 | ENSECAP00000006832 | ENSECAT00000009041 | NLRP1 | ENSECAG00000008523 | 14 |
| 76 | ENSECAP00000005894 | ENSECAT00000007948 | ALMS1 | ENSECAG00000007763 | 14 |
| 77 | ENSECAP00000005005 | ENSECAT00000006939 | LOC100072024 | ENSECAG00000006930 | 14 |
| 78 | ENSECAP00000001811 | ENSECAT00000002542 | LOC100070235 | ENSECAG00000002575 | 14 |
| 79 | ENSECAP00000022484 | ENSECAT00000026896 | LOC100059007 | ENSECAG00000024917 | 13 |
| 80 | ENSECAP00000019787 | ENSECAT00000023854 | C7AGG4_HORSE | ENSECAG00000022371 | 13 |
| 81 | ENSECAP00000017390 | ENSECAT00000021134 | Q38RB3_HORSE | ENSECAG00000019474 | 13 |
| 82 | ENSECAP00000015717 | ENSECAT00000019218 | DST | ENSECAG00000016469 | 13 |
| 83 | ENSECAP00000015777 | ENSECAT00000019286 | DST | ENSECAG00000016469 | 13 |
| 84 | ENSECAP00000013498 | ENSECAT00000016698 | Q0ZNY5_HORSE | ENSECAG00000015344 | 13 |
| 85 | ENSECAP00000011904 | ENSECAT00000014868 | LOC100055542 | ENSECAG00000014009 | 13 |
| 86 | ENSECAP00000006310 | ENSECAT00000008429 | LOC100147522 | ENSECAG00000008322 | 13 |
| 87 | ENSECAP00000003323 | ENSECAT00000004779 | LOC100053450 | ENSECAG00000004795 | 13 |
| 88 | ENSECAP00000000401 | ENSECAT00000000513 | | ENSECAG00000000505 | 13 |
| 89 | ENSECAP00000000238 | ENSECAT00000000310 | CDK5RAP2 | ENSECAG00000000135 | 13 |
| 90 | ENSECAP00000020282 | ENSECAT00000024416 | LOC100060845 | ENSECAG00000022610 | 12 |
| 91 | ENSECAP00000018568 | ENSECAT00000022463 | | ENSECAG00000020879 | 12 |
| 92 | ENSECAP00000020135 | ENSECAT00000024243 | NEB | ENSECAG00000020638 | 12 |
| 93 | ENSECAP00000018161 | ENSECAT00000021999 | Q30486_HORSE | ENSECAG00000019928 | 12 |
| 94 | ENSECAP00000018273 | ENSECAT00000022135 | Q30486_HORSE | ENSECAG00000019928 | 12 |
| 95 | ENSECAP00000018392 | ENSECAT00000022272 | LOC100066683 | ENSECAG00000019458 | 12 |
| 96 | ENSECAP00000018434 | ENSECAT00000022316 | LOC100066683 | ENSECAG00000019458 | 12 |
| 97 | ENSECAP00000011300 | ENSECAT00000014166 | AKNA | ENSECAG00000013443 | 12 |
| 98 | ENSECAP00000011127 | ENSECAT00000013970 | | ENSECAG00000012217 | 12 |
| 99 | ENSECAP00000009040 | ENSECAT00000011553 | A8DS43_HORSE | ENSECAG00000010990 | 12 |
| 100 | ENSECAP00000009178 | ENSECAT00000011706 | VWF | ENSECAG00000010778 | 12 |
| 101 | ENSECAP00000001933 | ENSECAT00000002701 | LOC100071709 | ENSECAG00000002762 | 12 |
| 102 | ENSECAP00000023060 | ENSECAT00000028999 | LOC100052379 | ENSECAG00000026927 | 11 |
| 103 | ENSECAP00000022716 | ENSECAT00000027151 | LOC100065703 | ENSECAG00000025146 | 11 |
| 104 | ENSECAP00000022623 | ENSECAT00000027044 | MYH2_HORSE | ENSECAG00000024667 | 11 |
| 105 | ENSECAP00000021590 | ENSECAT00000025925 | LOC100056231 | ENSECAG00000023986 | 11 |
| 106 | ENSECAP00000021611 | ENSECAT00000025949 | LOC100056231 | ENSECAG00000023986 | 11 |
| 107 | ENSECAP00000021595 | ENSECAT00000025930 | LOC100056231 | ENSECAG00000023986 | 11 |
| 108 | ENSECAP00000020412 | ENSECAT00000024564 | LOC100067431 | ENSECAG00000023006 | 11 |
| 109 | ENSECAP00000020442 | ENSECAT00000024599 | LOC100067431 | ENSECAG00000023006 | 11 |
| 110 | ENSECAP00000019559 | ENSECAT00000023605 | LOC100063263 | ENSECAG00000022166 | 11 |
| 111 | ENSECAP00000018443 | ENSECAT00000022326 | LOC100053918 | ENSECAG00000020384 | 11 |
| 112 | ENSECAP00000017567 | ENSECAT00000021339 | LOC100063079 | ENSECAG00000019716 | 11 |
| 113 | ENSECAP00000014769 | ENSECAT00000018123 | Q30469_HORSE | ENSECAG00000017143 | 11 |
| 114 | ENSECAP00000014009 | ENSECAT00000017262 | LOC100050626 | ENSECAG00000016442 | 11 |
| 115 | ENSECAP00000012986 | ENSECAT00000016099 | A8BJR0_HORSE | ENSECAG00000015188 | 11 |
| 116 | ENSECAP00000012792 | ENSECAT00000015871 | LOC100057368 | ENSECAG00000015097 | 11 |
| 117 | ENSECAP00000011729 | ENSECAT00000014661 | | ENSECAG00000013636 | 11 |
| 118 | ENSECAP00000010669 | ENSECAT00000013435 | ALPK2 | ENSECAG00000012921 | 11 |
| 119 | ENSECAP00000009322 | ENSECAT00000011877 | LOC100057663 | ENSECAG00000011097 | 11 |
| 120 | ENSECAP00000009558 | ENSECAT00000012133 | INTS1 | ENSECAG00000010792 | 11 |
| 121 | ENSECAP00000007604 | ENSECAT00000009916 | GOLGB1 | ENSECAG00000009513 | 11 |
| 122 | ENSECAP00000007413 | ENSECAT00000009695 | DQA | ENSECAG00000009142 | 11 |
| 123 | ENSECAP00000007042 | ENSECAT00000009278 | LOC100071075 | ENSECAG00000008291 | 11 |
| 124 | ENSECAP00000006530 | ENSECAT00000008686 | LOC100059800 | ENSECAG00000007640 | 11 |
| 125 | ENSECAP00000005674 | ENSECAT00000007707 | LOC100055532 | ENSECAG00000007490 | 11 |
| 126 | ENSECAP00000005477 | ENSECAT00000007492 | A3F2 | ENSECAG00000007480 | 11 |
| 127 | ENSECAP00000001525 | ENSECAT00000002154 | LOC100064026 | ENSECAG00000000744 | 11 |
| 128 | ENSECAP00000021338 | ENSECAT00000025639 | LOC100062791 | ENSECAG00000023761 | 10 |
| 129 | ENSECAP00000020670 | ENSECAT00000024857 | PALLD | ENSECAG00000023122 | 10 |
| 130 | ENSECAP00000020604 | ENSECAT00000024782 | LOC100067571 | ENSECAG00000023096 | 10 |
| 131 | ENSECAP00000020441 | ENSECAT00000024598 | LOC100051829 | ENSECAG00000022735 | 10 |
| 132 | ENSECAP00000020445 | ENSECAT00000024602 | LOC100051829 | ENSECAG00000022735 | 10 |
| 133 | ENSECAP00000019854 | ENSECAT00000023927 | PAM | ENSECAG00000021895 | 10 |
| 134 | ENSECAP00000019816 | ENSECAT00000023883 | PAM | ENSECAG00000021895 | 10 |
| 135 | ENSECAP00000019858 | ENSECAT00000023931 | PAM | ENSECAG00000021895 | 10 |
| 136 | ENSECAP00000019060 | ENSECAT00000023032 | LOC100061960 | ENSECAG00000021649 | 10 |
| 137 | ENSECAP00000019067 | ENSECAT00000023039 | LOC100061960 | ENSECAG00000021649 | 10 |
| 138 | ENSECAP00000019305 | ENSECAT00000023320 | ATM | ENSECAG00000021147 | 10 |
| 139 | ENSECAP00000019300 | ENSECAT00000023313 | ATM | ENSECAG00000021147 | 10 |
| 140 | ENSECAP00000019275 | ENSECAT00000023286 | LOC100057490 | ENSECAG00000020991 | 10 |
| 141 | ENSECAP00000018459 | ENSECAT00000022345 | LOC100050762 | ENSECAG00000020944 | 10 |
| 142 | ENSECAP00000018316 | ENSECAT00000022181 | LOC100054381 | ENSECAG00000020551 | 10 |
| 143 | ENSECAP00000016551 | ENSECAT00000020181 | Q95MC4_HORSE | ENSECAG00000018853 | 10 |
| 144 | ENSECAP00000018019 | ENSECAT00000021847 | AOX1 | ENSECAG00000018147 | 10 |
| 145 | ENSECAP00000018027 | ENSECAT00000021855 | AOX1 | ENSECAG00000018147 | 10 |
| 146 | ENSECAP00000017845 | ENSECAT00000021655 | AOX1 | ENSECAG00000018147 | 10 |
| 147 | ENSECAP00000017729 | ENSECAT00000021522 | AOX1 | ENSECAG00000018147 | 10 |
| 148 | ENSECAP00000017778 | ENSECAT00000021577 | AOX1 | ENSECAG00000018147 | 10 |
| 149 | ENSECAP00000015802 | ENSECAT00000019314 | MAST4 | ENSECAG00000017672 | 10 |
| 150 | ENSECAP00000015250 | ENSECAT00000018685 | NLRP12 | ENSECAG00000017662 | 10 |
| 151 | ENSECAP00000016049 | ENSECAT00000019599 | MYOM2 | ENSECAG00000017592 | 10 |
| 152 | ENSECAP00000015006 | ENSECAT00000018396 | LOC100072177 | ENSECAG00000016641 | 10 |
| 153 | ENSECAP00000012829 | ENSECAT00000015913 | LOC100054217 | ENSECAG00000014934 | 10 |
| 154 | ENSECAP00000013082 | ENSECAT00000016211 | LOC100058067 | ENSECAG00000014711 | 10 |
| 155 | ENSECAP00000022902 | ENSECAT00000028943 | SWT1 | ENSECAG00000012085 | 10 |
| 156 | ENSECAP00000010291 | ENSECAT00000013008 | SPTB | ENSECAG00000011792 | 10 |
| 157 | ENSECAP00000009041 | ENSECAT00000011554 | SOLH | ENSECAG00000011148 | 10 |
| 158 | ENSECAP00000010079 | ENSECAT00000012758 | LOC100050706 | ENSECAG00000010913 | 10 |
| 159 | ENSECAP00000008683 | ENSECAT00000011146 | HIVEP1 | ENSECAG00000010759 | 10 |
| 160 | ENSECAP00000008215 | ENSECAT00000010622 | LOC100061193 | ENSECAG00000010255 | 10 |
| 161 | ENSECAP00000008746 | ENSECAT00000011217 | | ENSECAG00000010013 | 10 |
| 162 | ENSECAP00000008764 | ENSECAT00000011239 | | ENSECAG00000010013 | 10 |
| 163 | ENSECAP00000008667 | ENSECAT00000011129 | | ENSECAG00000010013 | 10 |
| 164 | ENSECAP00000007671 | ENSECAT00000009989 | PARP4 | ENSECAG00000009458 | 10 |
| 165 | ENSECAP00000006999 | ENSECAT00000009231 | LOC100051158 | ENSECAG00000008537 | 10 |
| 166 | ENSECAP00000005883 | ENSECAT00000007937 | LOC100063370 | ENSECAG00000007621 | 10 |
| 167 | ENSECAP00000006018 | ENSECAT00000008088 | A8DS41_HORSE | ENSECAG00000007192 | 10 |
| 168 | ENSECAP00000002220 | ENSECAT00000003107 | FRIL_HORSE | ENSECAG00000003192 | 10 |
| 169 | ENSECAP00000001048 | ENSECAT00000001358 | LOC100061825 | ENSECAG00000001481 | 10 |
| 170 | ENSECAP00000000750 | ENSECAT00000000926 | LOC100052422 | ENSECAG00000001003 | 10 |

**Supplementary Figure S6. GO classification of all expressed genes in human, mouse, and horse muscle tissue.**

1. Biological process

**
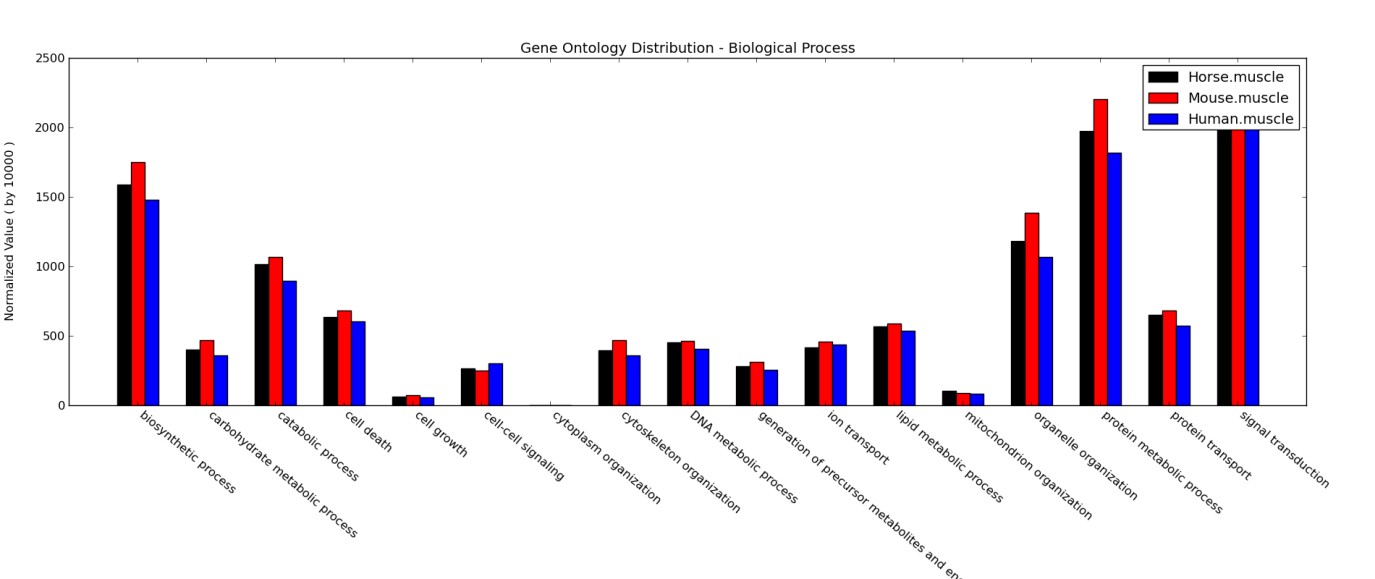
**

1. Molecular function

**
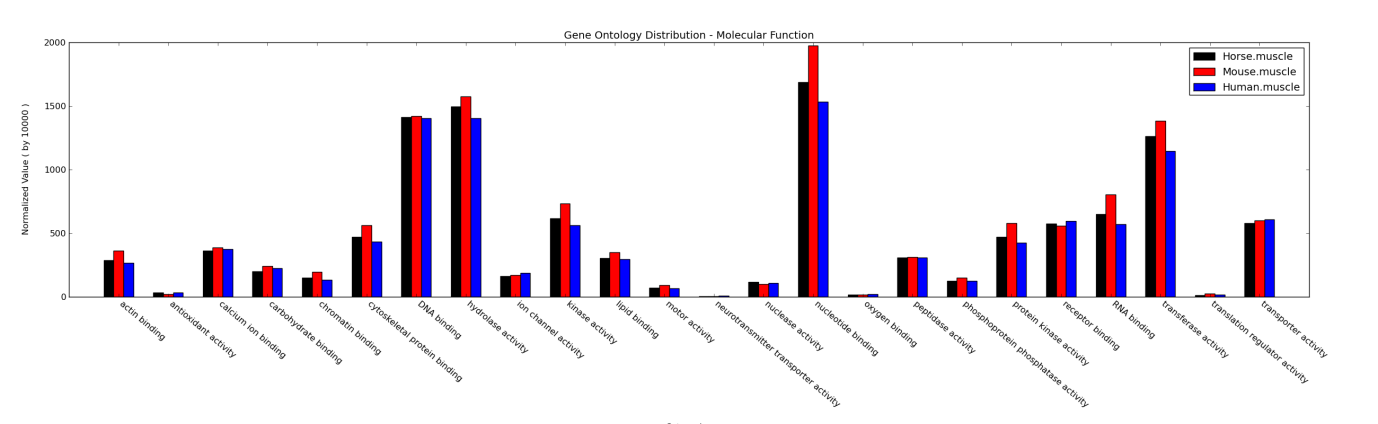
**

1. Cellular process

**
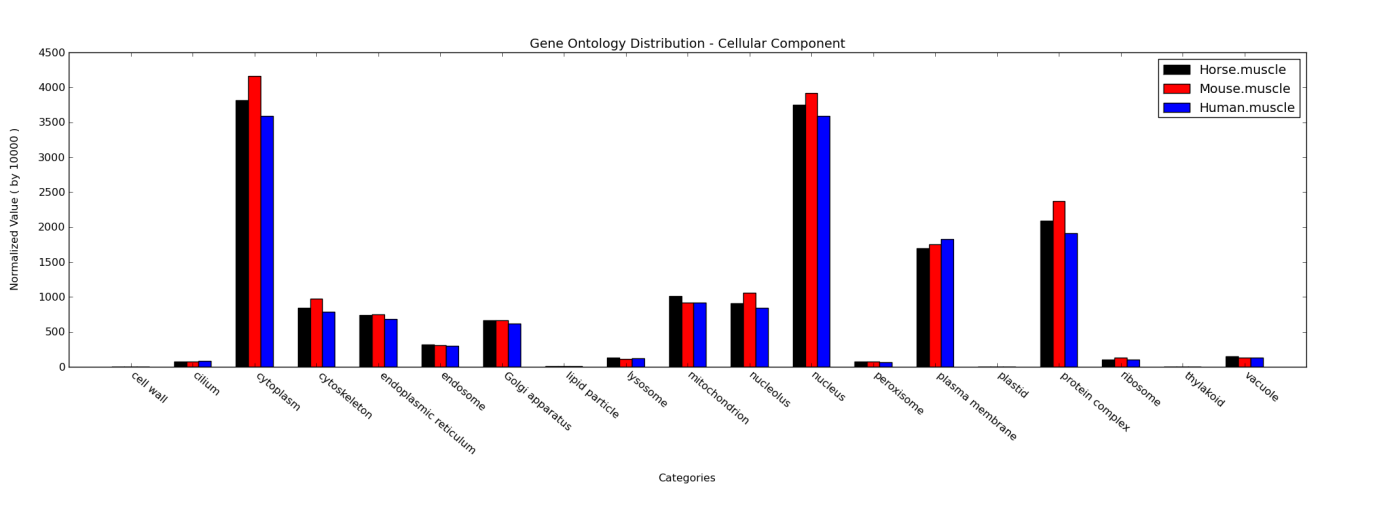
**

**Supplementary Figure S7. GO classification of all expressed horse genes in blood and muscle tissue.**

1. Biological process

**
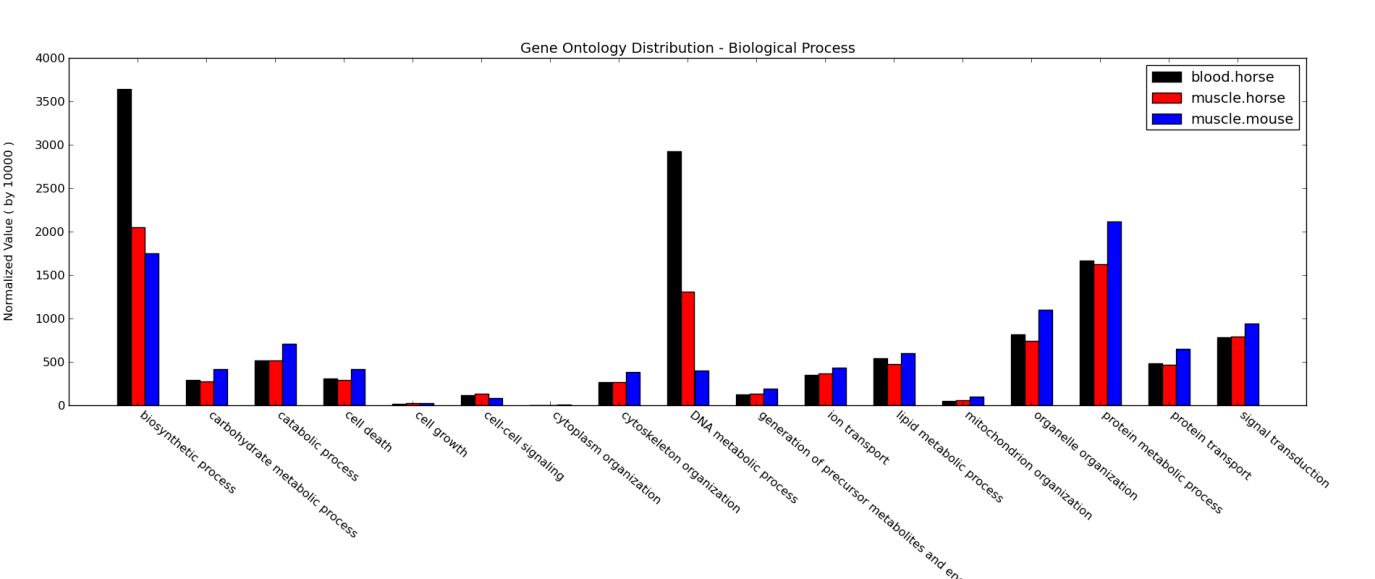
**

1. Molecular function

**
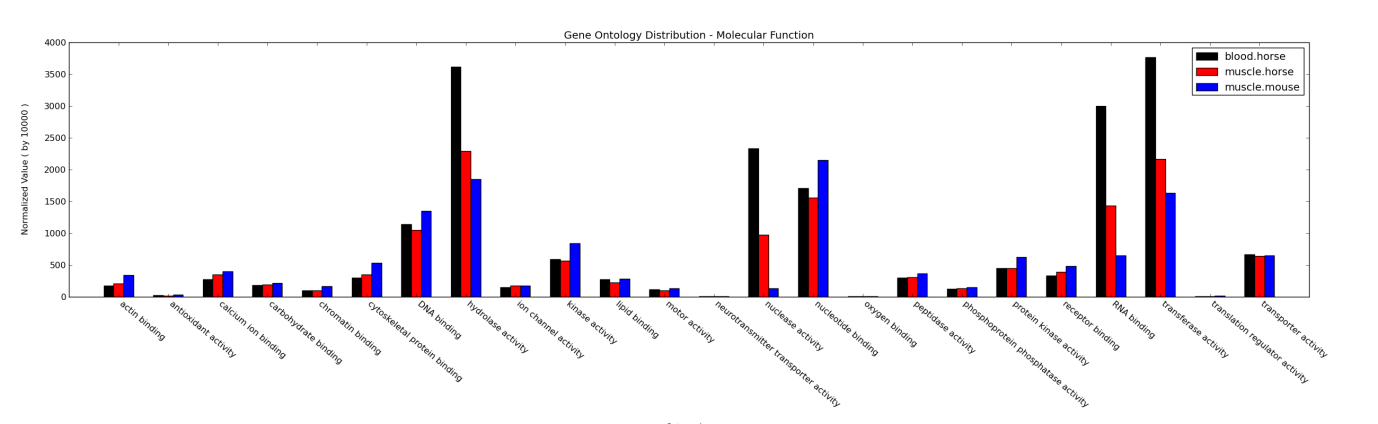
**

1. Cellular component

**
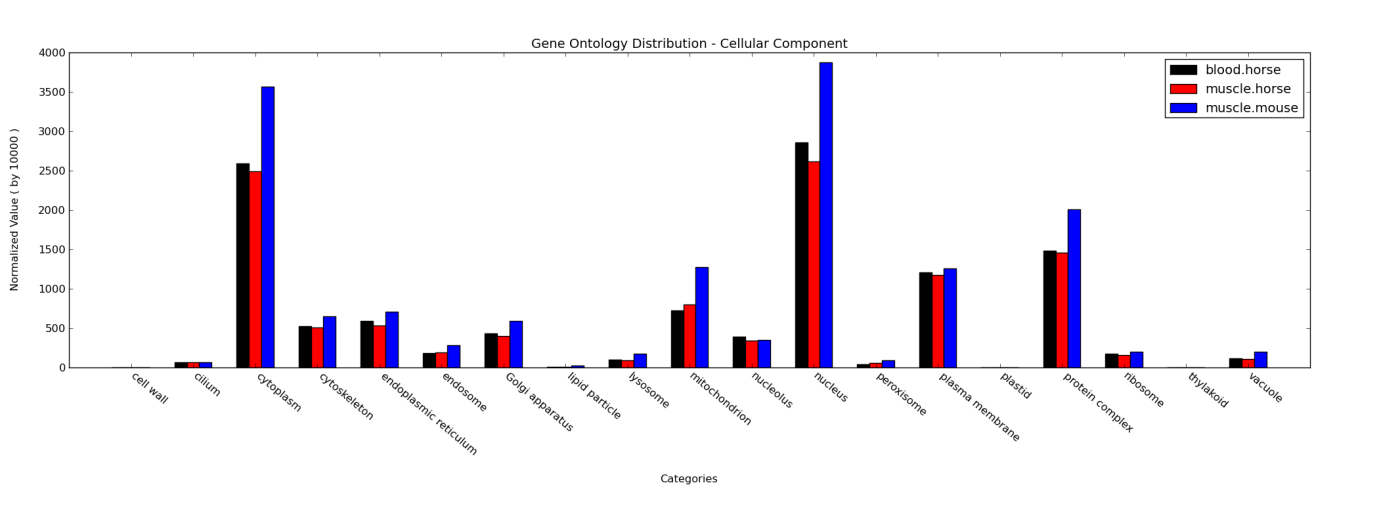
**

**Supplementary Figure S8. Correlation matrix of the 24 samples.**


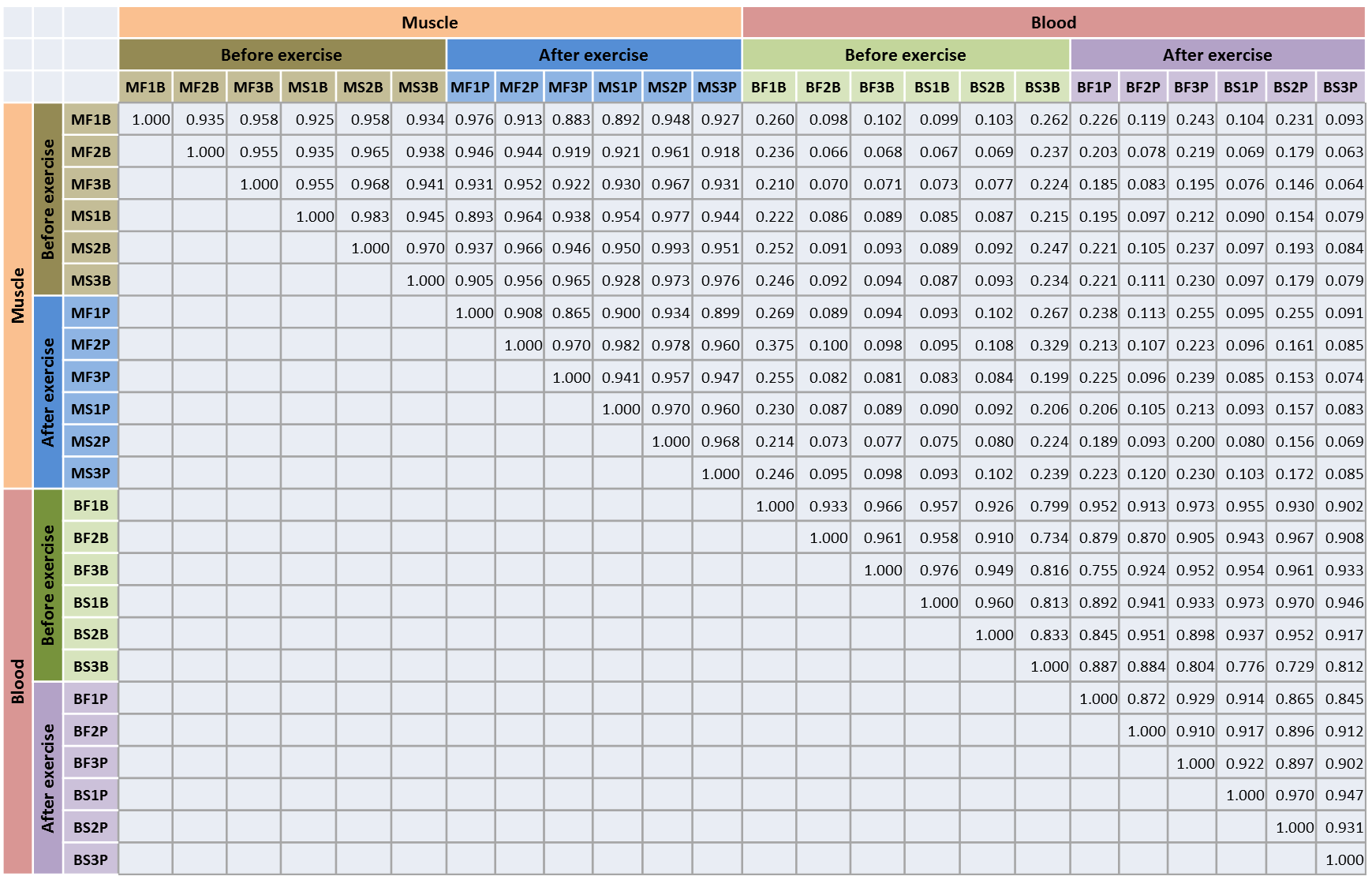


**Supplementary Figure S9. Correlation matrix of three human samples from kidney and liver tissues.**


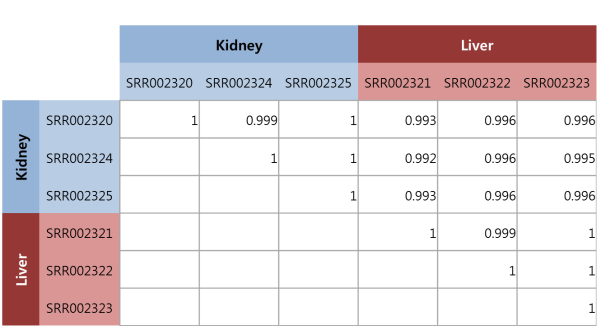


**Supplementary Figure S10. Histogram of average expression level of the unigene clusters in the 24 samples.**


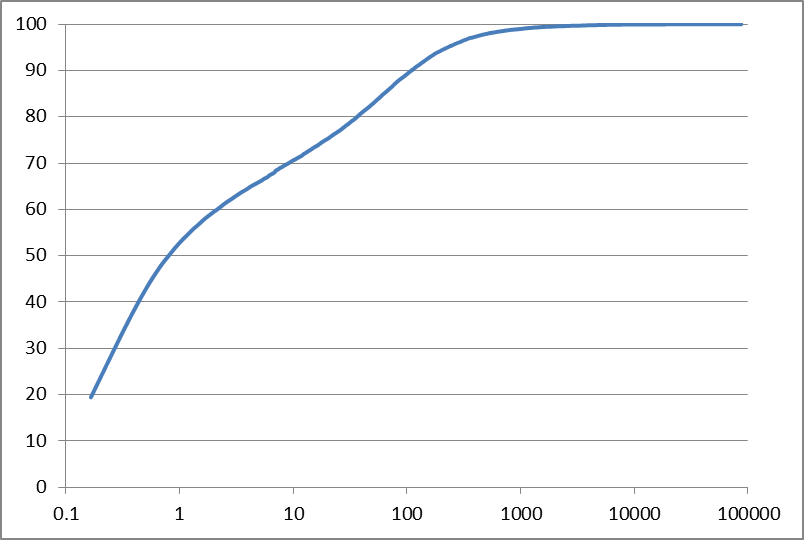


**Supplementary Table S16. List of DEGs in muscle and blood tissues.**

1. List of up-regulated DEGs in muscle tissue

| **Transcript name** | **Gene name (Ensembl)** | **Gene name** | **Average log2 ratio** |
| --- | --- | --- | --- |
| Cluster 26390 | Novel gene |  | 0.85 |
| Cluster 31835 | Novel gene |  | 1.16 |
| Cluster 36948 | Novel gene |  | 2.41 |
| Cluster 505 | Novel gene |  | 1.07 |
| Cluster 6966 | Novel gene |  | 1.68 |
| ENSECAT00000000057 | ENSECAG00000000063 | LOC100065129 | 1.89 |
| ENSECAT00000000076 | ENSECAG00000000074 | LOC100056359 | 3.09 |
| ENSECAT00000000116 | ENSECAG00000000090 | MAP3K8 | 2.7 |
| ENSECAT00000000154 | ENSECAG00000000153 | LOC100050197 | 3.14 |
| ENSECAT00000000159 | ENSECAG00000000186 | LOC100055442 | 2.17 |
| ENSECAT00000000191 | ENSECAG00000000096 | LOC100054432 | 1.08 |
| ENSECAT00000000193 | Known psuedogene | | 1.79 |
| ENSECAT00000000213 | ENSECAG00000000266 | LOC100062395 | 3.23 |
| ENSECAT00000000230 | ENSECAG00000000173 | LOC100062559 | 2.33 |
| ENSECAT00000000254 | ENSECAG00000000122 | GDA | 1.08 |
| ENSECAT00000000312 | ENSECAG00000000382 | CLEC2B | 3.71 |
| ENSECAT00000000351 | ENSECAG00000000387 | LOC100050110 | 1.19 |
| ENSECAT00000000475 | ENSECAG00000000400 | LOC100062876 | 1.39 |
| ENSECAT00000000484 | ENSECAG00000000377 | LOC100053899 | 0.97 |
| ENSECAT00000000516 | ENSECAG00000000303 | Q45FB0_HORSE | 2.17 |
| ENSECAT00000000538 | ENSECAG00000000466 | LOC100070980 | 1.34 |
| ENSECAT00000000567 | ENSECAG00000000464 | | 1.32 |
| ENSECAT00000000572 | ENSECAG00000000395 | RAPGEF5 | 1.72 |
| ENSECAT00000000613 | ENSECAG00000000642 | LOC100069588 | 1.3 |
| ENSECAT00000000676 | ENSECAG00000000763 | LOC100068414 | 5.31 |
| ENSECAT00000000697 | ENSECAG00000000298 | LOC100052312 | 1.02 |
| ENSECAT00000000703 | ENSECAG00000000874 | LOC100057225 | 3.83 |
| ENSECAT00000000747 | ENSECAG00000000901 | LOC100057968 | 1.4 |
| ENSECAT00000000781 | ENSECAG00000000591 | LOC100066812 | 1.59 |
| ENSECAT00000000828 | ENSECAG00000000624 | LOC100057795 | 1 |
| ENSECAT00000000902 | ENSECAG00000001069 | LOC100054419 | 2.79 |
| ENSECAT00000000944 | ENSECAG00000000650 | NPL | 1.72 |
| ENSECAT00000001012 | ENSECAG00000001110 | LOC100050888 | 2.24 |
| ENSECAT00000001035 | ENSECAG00000001037 | Q95JF0_HORSE | 1.96 |
| ENSECAT00000001397 | ENSECAG00000001554 | LOC100050149 | 1.95 |
| ENSECAT00000001504 | ENSECAG00000001495 | LOC100059035 | 1.47 |
| ENSECAT00000001514 | ENSECAG00000001674 | LOC100056419 | 1.4 |
| ENSECAT00000001574 | ENSECAG00000001129 | LOC100069058 | 3.35 |
| ENSECAT00000001658 | ENSECAG00000001784 | Q9TTT3_HORSE | 3.68 |
| ENSECAT00000001723 | ENSECAG00000001863 | LOC100058694 | 3.15 |
| ENSECAT00000001758 | ENSECAG00000001900 | LOC100058739 | 3.28 |
| ENSECAT00000001865 | ENSECAG00000000383 | LOC100070532 | 2.4 |
| ENSECAT00000001957 | ENSECAG00000002090 | | 2.29 |
| ENSECAT00000002028 | ENSECAG00000002176 | | 1.51 |
| ENSECAT00000002055 | ENSECAG00000002212 | LOC100050481 | 1.87 |
| ENSECAT00000002198 | ENSECAG00000002250 | LOC100050086 | 1.02 |
| ENSECAT00000002269 | ENSECAG00000002437 | LOC100062993 | 2.19 |
| ENSECAT00000002299 | ENSECAG00000001774 | Q4JIK2_HORSE | 1.53 |
| ENSECAT00000002340 | ENSECAG00000002387 | A5JUZ4_HORSE | 1.38 |
| ENSECAT00000002355 | Known psuedogene | | 1.4 |
| ENSECAT00000002468 | ENSECAG00000000816 | LOC100055812 | 2.14 |
| ENSECAT00000002568 | Known psuedogene | | 1.13 |
| ENSECAT00000002615 | ENSECAG00000000480 | ABI3BP | 0.7 |
| ENSECAT00000002767 | ENSECAG00000002142 | LOC100069114 | 1.73 |
| ENSECAT00000002798 | ENSECAG00000001581 | MAP7D1 | 3.75 |
| ENSECAT00000002905 | ENSECAG00000002186 | Q6RG76_HORSE | 3.4 |
| ENSECAT00000002908 | ENSECAG00000002992 | LOC100055994 | 2.14 |
| ENSECAT00000002912 | ENSECAG00000003072 | LOC100050602 | 2.88 |
| ENSECAT00000002920 | ENSECAG00000022502 | LOC100072399 | 0.7 |
| ENSECAT00000003107 | ENSECAG00000003192 | FRIL_HORSE | 2.02 |
| ENSECAT00000003156 | ENSECAG00000003315 | LOC100146249 | 3.02 |
| ENSECAT00000003237 | ENSECAG00000003398 | LOC100072561 | 1.45 |
| ENSECAT00000003301 | ENSECAG00000003428 | UBIQ_HORSE | 1.25 |
| ENSECAT00000003346 | ENSECAG00000003495 | LOC100063334 | 1.49 |
| ENSECAT00000003374 | ENSECAG00000003516 | PNRC1 | 1.17 |
| ENSECAT00000003445 | ENSECAG00000001445 | LOC100052622 | 1.36 |
| ENSECAT00000003662 | ENSECAG00000003386 | LOC100051825 | 1.07 |
| ENSECAT00000003664 | ENSECAG00000001989 | LOC100056552 | 1.75 |
| ENSECAT00000003672 | ENSECAG00000003772 | LOC100065272 | 1.01 |
| ENSECAT00000003723 | ENSECAG00000003277 | LOC100065217 | 2.35 |
| ENSECAT00000003759 | ENSECAG00000003816 | LOC100146801 | 5.11 |
| ENSECAT00000003790 | ENSECAG00000003823 | LOC100050888 | 2.17 |
| ENSECAT00000003900 | ENSECAG00000004005 | MESDC1 | 2.58 |
| ENSECAT00000003925 | ENSECAG00000002988 | PSTPIP1 | 2.06 |
| ENSECAT00000003977 | ENSECAG00000004066 | LOC100066662 | 2.2 |
| ENSECAT00000004041 | ENSECAG00000004162 | LOC100058650 | 2.15 |
| ENSECAT00000004048 | ENSECAG00000003761 | LOC100062761 | 1.15 |
| ENSECAT00000004096 | ENSECAG00000004055 | LOC100073278 | 1.09 |
| ENSECAT00000004120 | ENSECAG00000004249 | LOC100067299 | 1.59 |
| ENSECAT00000004150 | ENSECAG00000004264 | VASN | 2.38 |
| ENSECAT00000004200 | ENSECAG00000002866 | Q3KS03_HORSE | 2.98 |
| ENSECAT00000004209 | ENSECAG00000001700 | LOC100071237 | 1.2 |
| ENSECAT00000004262 | ENSECAG00000004349 | LOC100071577 | 1.49 |
| ENSECAT00000004385 | ENSECAG00000004492 | LOC100052275 | 1.89 |
| ENSECAT00000004408 | ENSECAG00000004433 | LOC100071588 | 1.33 |
| ENSECAT00000004571 | ENSECAG00000004159 | BCL9 | 0.93 |
| ENSECAT00000004600 | ENSECAG00000004725 | XIRP1 | 4.45 |
| ENSECAT00000004642 | ENSECAG00000004762 | NRIP1 | 1.27 |
| ENSECAT00000004653 | ENSECAG00000004773 | LOC100073065 | 2.55 |
| ENSECAT00000004683 | ENSECAG00000004763 | | 4.62 |
| ENSECAT00000004699 | ENSECAG00000003462 | RUNX1 | 4.9 |
| ENSECAT00000004778 | ENSECAG00000004444 | LOC100054000 | 1.34 |
| ENSECAT00000004802 | ENSECAG00000004810 | Q9BFW9_HORSE | 1.7 |
| ENSECAT00000004830 | ENSECAG00000002321 | SEC24A | 2.6 |
| ENSECAT00000004884 | ENSECAG00000004791 | TICAM1 | 1.48 |
| ENSECAT00000004978 | ENSECAG00000005090 | LOC100069914 | 2.67 |
| ENSECAT00000005046 | ENSECAG00000004871 | LOC100064560 | 2.41 |
| ENSECAT00000005059 | ENSECAG00000004975 | NUFIP2 | 1.5 |
| ENSECAT00000005118 | ENSECAG00000005241 | LOC100146206 | 4.18 |
| ENSECAT00000005150 | ENSECAG00000005199 | LOC100071280 | 2.26 |
| ENSECAT00000005310 | ENSECAG00000005387 | LOC100054342 | 1.14 |
| ENSECAT00000005363 | ENSECAG00000005467 | LOC100070269 | 1.61 |
| ENSECAT00000005391 | ENSECAG00000005489 | LOC100056682 | 3.41 |
| ENSECAT00000005416 | ENSECAG00000005267 | LOC100065784 | 1.9 |
| ENSECAT00000005498 | ENSECAG00000005525 | Q8WMP0_HORSE | 2.64 |
| ENSECAT00000005541 | ENSECAG00000005628 | LOC100057288 | 1.9 |
| ENSECAT00000005565 | ENSECAG00000005649 | LOC100071257 | 1.88 |
| ENSECAT00000005750 | ENSECAG00000004312 | IL1RA_HORSE | 5.79 |
| ENSECAT00000005851 | ENSECAG00000005675 | NFE2L2 | 1.52 |
| ENSECAT00000006007 | ENSECAG00000005814 | NEDD9 | 1.88 |
| ENSECAT00000006093 | ENSECAG00000005305 | BACE2 | 1.7 |
| ENSECAT00000006120 | ENSECAG00000005708 | ABL2 | 1.99 |
| ENSECAT00000006133 | ENSECAG00000006153 | LOC100065997 | 1.92 |
| ENSECAT00000006134 | ENSECAG00000005685 | LOC100061076 | 1.98 |
| ENSECAT00000006222 | ENSECAG00000006067 | USP48 | 1.21 |
| ENSECAT00000006237 | ENSECAG00000001652 | ITGA6 | 2.66 |
| ENSECAT00000006238 | ENSECAG00000006234 | LOC100050100 | 3.78 |
| ENSECAT00000006248 | ENSECAG00000002676 | SMARCA5 | 1.18 |
| ENSECAT00000006373 | ENSECAG00000005570 | TMEM49 | 2.77 |
| ENSECAT00000006406 | ENSECAG00000005905 | SMOX | 1.38 |
| ENSECAT00000006415 | ENSECAG00000005853 | TAF5 | 1.76 |
| ENSECAT00000006606 | ENSECAG00000006436 | LOC100055104 | 1.97 |
| ENSECAT00000006644 | ENSECAG00000003599 | LOC100051016 | 1.57 |
| ENSECAT00000006652 | ENSECAG00000006595 | LOC100055552 | 2.31 |
| ENSECAT00000006682 | ENSECAG00000006474 | LOC100050367 | 0.93 |
| ENSECAT00000006703 | ENSECAG00000006288 | LOC100057456 | 4.16 |
| ENSECAT00000006705 | ENSECAG00000006711 | LOC100058291 | 2.57 |
| ENSECAT00000006747 | ENSECAG00000006453 | LOC100052831 | 1.85 |
| ENSECAT00000006807 | ENSECAG00000006400 | LOC100059448 | 1.17 |
| ENSECAT00000006828 | ENSECAG00000006600 | ZSWIM4 | 1.14 |
| ENSECAT00000006848 | ENSECAG00000006867 | LOC100056801 | 1.62 |
| ENSECAT00000006852 | ENSECAG00000006674 | LOC100065651 | 6.09 |
| ENSECAT00000006865 | ENSECAG00000006472 | LOC100066216 | 1.3 |
| ENSECAT00000006900 | ENSECAG00000006931 | LOC100070334 | 1.07 |
| ENSECAT00000006907 | ENSECAG00000005741 | LOC100070256 | 1.32 |
| ENSECAT00000006938 | ENSECAG00000005927 | TP53BP2 | 1.22 |
| ENSECAT00000006984 | ENSECAG00000006965 | LOC100062278 | 1.41 |
| ENSECAT00000007051 | ENSECAG00000007021 | LOC100057885 | 1.99 |
| ENSECAT00000007104 | ENSECAG00000007088 | LOC100064087 | 1.32 |
| ENSECAT00000007109 | ENSECAG00000006749 | ATP13A3 | 1.41 |
| ENSECAT00000007148 | ENSECAG00000006442 | LOC100058578 | 1.73 |
| ENSECAT00000007186 | ENSECAG00000006915 | LOC100064585 | 2.29 |
| ENSECAT00000007198 | ENSECAG00000007191 | LOC100053991 | 1.05 |
| ENSECAT00000007199 | ENSECAG00000006923 | LOC100062536 | 1.86 |
| ENSECAT00000007204 | ENSECAG00000000701 | FINC_HORSE | 19.19 |
| ENSECAT00000007212 | ENSECAG00000000701 | FINC_HORSE | 1.18 |
| ENSECAT00000007281 | ENSECAG00000006984 | ZCCHC8 | 1.04 |
| ENSECAT00000007455 | ENSECAG00000006575 | LOC100059816 | 1.02 |
| ENSECAT00000007483 | ENSECAG00000007164 | LOC100067099 | 1.71 |
| ENSECAT00000007495 | ENSECAG00000007359 | LOC100052549 | 1.79 |
| ENSECAT00000007523 | ENSECAG00000007095 | LOC100063506 | 2.67 |
| ENSECAT00000007540 | ENSECAG00000007444 | LOC100068453 | 1.65 |
| ENSECAT00000007605 | ENSECAG00000007476 | LOC100065672 | 1.56 |
| ENSECAT00000007620 | ENSECAG00000007391 | LOC100067333 | 1.06 |
| ENSECAT00000007639 | ENSECAG00000007518 | LOC100063655 | 1.75 |
| ENSECAT00000007651 | ENSECAG00000007210 | LOC100061532 | 1.46 |
| ENSECAT00000007679 | ENSECAG00000006078 | EIF2C2 | 1.92 |
| ENSECAT00000007692 | ENSECAG00000007266 | | 1.82 |
| ENSECAT00000007708 | ENSECAG00000007351 | Q95M89_HORSE | 2.15 |
| ENSECAT00000007733 | ENSECAG00000007240 | CYLD | 1.04 |
| ENSECAT00000007737 | ENSECAG00000007626 | LOC100051083 | 2 |
| ENSECAT00000007857 | ENSECAG00000007625 | LOC100050034 | 2.44 |
| ENSECAT00000007863 | ENSECAG00000007789 | LOC100070766 | 1.28 |
| ENSECAT00000007886 | ENSECAG00000007837 | LOC100056804 | 1.06 |
| ENSECAT00000008054 | ENSECAG00000008015 | LOC100066128 | 1.11 |
| ENSECAT00000008075 | ENSECAG00000007156 | | 2.16 |
| ENSECAT00000008088 | ENSECAG00000007192 | A8DS41_HORSE | 1.46 |
| ENSECAT00000008114 | ENSECAG00000007894 | RNF19A | 1.43 |
| ENSECAT00000008141 | ENSECAG00000008096 | FAM111B | 3.36 |
| ENSECAT00000008154 | ENSECAG00000007818 | LOC100070851 | 1.26 |
| ENSECAT00000008241 | ENSECAG00000007751 | BAZ1A | 2.29 |
| ENSECAT00000008246 | ENSECAG00000007576 | SLC38A1 | 2.12 |
| ENSECAT00000008260 | ENSECAG00000007878 | FRMD3 | 1.05 |
| ENSECAT00000008287 | ENSECAG00000007952 | LOC100054237 | 1.12 |
| ENSECAT00000008354 | ENSECAG00000008273 | LOC100060911 | 1.37 |
| ENSECAT00000008490 | ENSECAG00000007796 | BTAF1 | 1.08 |
| ENSECAT00000008522 | ENSECAG00000007962 | LOC100050551 | 1.38 |
| ENSECAT00000008536 | ENSECAG00000007942 | LOC100073057 | 3.29 |
| ENSECAT00000008556 | ENSECAG00000008261 | LOC100057010 | 1.55 |
| ENSECAT00000008601 | ENSECAG00000008288 | PAK1IP1 | 1.3 |
| ENSECAT00000008608 | ENSECAG00000008287 | LOC100057232 | 1.88 |
| ENSECAT00000008661 | ENSECAG00000008162 | LOC100060169 | 1.09 |
| ENSECAT00000008669 | ENSECAG00000008227 | LOC100053404 | 2.8 |
| ENSECAT00000008675 | ENSECAG00000008327 | PI4K2A | 1.95 |
| ENSECAT00000008682 | ENSECAG00000007709 | SEMA3F | 1.95 |
| ENSECAT00000008686 | ENSECAG00000007640 | LOC100059800 | 0.93 |
| ENSECAT00000008745 | ENSECAG00000008335 | LOC100052078 | 5.34 |
| ENSECAT00000008809 | ENSECAG00000008107 | FERMT3 | 1.37 |
| ENSECAT00000008836 | ENSECAG00000008623 | SPRED1 | 1.91 |
| ENSECAT00000008950 | ENSECAG00000008811 | KCNJ15 | 2.78 |
| ENSECAT00000008953 | ENSECAG00000008668 | Q8MKE0_HORSE | 4.84 |
| ENSECAT00000009045 | ENSECAG00000008777 | LOC100065720 | 1.11 |
| ENSECAT00000009059 | ENSECAG00000008912 | LOC100057048 | 1.73 |
| ENSECAT00000009103 | ENSECAG00000008585 | LOC100066877 | 1.59 |
| ENSECAT00000009151 | ENSECAG00000008851 | LOC100050292 | 1.89 |
| ENSECAT00000009173 | ENSECAG00000009012 | LOC100063913 | 2.77 |
| ENSECAT00000009174 | ENSECAG00000007650 | EPHA2 | 2.57 |
| ENSECAT00000009183 | ENSECAG00000008693 | PTPN12 | 1.27 |
| ENSECAT00000009278 | ENSECAG00000008291 | LOC100071075 | 1.21 |
| ENSECAT00000009299 | ENSECAG00000009046 | LOC100060850 | 1.01 |
| ENSECAT00000009303 | ENSECAG00000009067 | SLC16A6 | 1.85 |
| ENSECAT00000009310 | ENSECAG00000009168 | LOC100059768 | 3.79 |
| ENSECAT00000009314 | ENSECAG00000009044 | PRDM1 | 2.09 |
| ENSECAT00000009322 | Known psuedogene | | 2.09 |
| ENSECAT00000009345 | ENSECAG00000009192 | LOC100063248 | 2.76 |
| ENSECAT00000009359 | ENSECAG00000008705 | LOC100050053 | 1.56 |
| ENSECAT00000009562 | ENSECAG00000009277 | LOC100057639 | 1.36 |
| ENSECAT00000009573 | ENSECAG00000008927 | HIPK2 | 1.77 |
| ENSECAT00000009596 | ENSECAG00000009221 | NSUN6 | 1.54 |
| ENSECAT00000009616 | ENSECAG00000009363 | LOC100052594 | 2.59 |
| ENSECAT00000009669 | ENSECAG00000009354 | LOC100071443 | 1.01 |
| ENSECAT00000009682 | ENSECAG00000009306 | PVRL2 | 0.96 |
| ENSECAT00000009705 | ENSECAG00000009164 | LOC100055716 | 1.89 |
| ENSECAT00000009707 | ENSECAG00000008923 | THBS1 | 5.71 |
| ENSECAT00000009725 | ENSECAG00000009535 | FCER1G | 2.25 |
| ENSECAT00000009748 | ENSECAG00000009482 | PPRC1 | 1.8 |
| ENSECAT00000009761 | ENSECAG00000009562 | LOC100069173 | 3.6 |
| ENSECAT00000009805 | ENSECAG00000009481 | LOC100066682 | 1.14 |
| ENSECAT00000009845 | ENSECAG00000009402 | VEGFA_HORSE | 2.59 |
| ENSECAT00000009850 | ENSECAG00000009402 | VEGFA_HORSE | 1.55 |
| ENSECAT00000009864 | ENSECAG00000009400 | LOC100055139 | 2.85 |
| ENSECAT00000009884 | ENSECAG00000008866 | MAST2 | 0.5 |
| ENSECAT00000009890 | ENSECAG00000009572 | PLIN3 | 1.29 |
| ENSECAT00000009924 | ENSECAG00000009261 | LOC100055508 | 2.07 |
| ENSECAT00000009946 | ENSECAG00000009722 | YOD1 | 6.7 |
| ENSECAT00000010015 | ENSECAG00000009626 | LOC100073197 | 2.15 |
| ENSECAT00000010020 | ENSECAG00000009744 | IRF7 | 1.37 |
| ENSECAT00000010038 | ENSECAG00000009787 | LOC100071451 | 2.5 |
| ENSECAT00000010040 | ENSECAG00000008876 | PTPRE | 4 |
| ENSECAT00000010051 | ENSECAG00000009303 | LOC100067002 | 3.09 |
| ENSECAT00000010162 | ENSECAG00000009828 | LOC100073033 | 3.15 |
| ENSECAT00000010165 | ENSECAG00000008876 | PTPRE | 3.4 |
| ENSECAT00000010174 | ENSECAG00000009958 | LOC100069834 | 2.41 |
| ENSECAT00000010183 | ENSECAG00000009680 | LOC100058853 | 1.82 |
| ENSECAT00000010285 | ENSECAG00000010042 | LOC100050794 | 1.4 |
| ENSECAT00000010307 | ENSECAG00000009570 | LOC100058419 | 1.66 |
| ENSECAT00000010348 | ENSECAG00000010087 | LOC100058238 | 1.51 |
| ENSECAT00000010372 | ENSECAG00000009902 | IRF5 | 1.14 |
| ENSECAT00000010456 | ENSECAG00000010146 | ZSCAN10 | 1.48 |
| ENSECAT00000010476 | ENSECAG00000010231 | IL18BP | 1.04 |
| ENSECAT00000010481 | ENSECAG00000010107 | LOC100053621 | 1.07 |
| ENSECAT00000010506 | ENSECAG00000009896 | ILEU_HORSE | 1.56 |
| ENSECAT00000010526 | Known psuedogene | | 1.29 |
| ENSECAT00000010528 | ENSECAG00000009648 | CLIP1 | 2.38 |
| ENSECAT00000010529 | ENSECAG00000010174 | LOC100146263 | 1.26 |
| ENSECAT00000010557 | ENSECAG00000010277 | LOC100051382 | 2.57 |
| ENSECAT00000010579 | ENSECAG00000009799 | LOC100051146 | 1.5 |
| ENSECAT00000010606 | ENSECAG00000010340 | LOC100070825 | 1.84 |
| ENSECAT00000010637 | ENSECAG00000010015 | LOC100070128 | 1.09 |
| ENSECAT00000010683 | ENSECAG00000010349 | LOC100058902 | 1.61 |
| ENSECAT00000010707 | ENSECAG00000010339 | TLR4_HORSE | 3.22 |
| ENSECAT00000010710 | ENSECAG00000010325 | LOC100064427 | 1.74 |
| ENSECAT00000010751 | ENSECAG00000010418 | LOC100056176 | 1.98 |
| ENSECAT00000010825 | ENSECAG00000010260 | LOC100063402 | 1.32 |
| ENSECAT00000010837 | ENSECAG00000010503 | ZNF398 | 1.86 |
| ENSECAT00000010883 | ENSECAG00000009901 | LOC100058480 | 1.51 |
| ENSECAT00000010895 | ENSECAG00000010546 | LOC100052058 | 2.53 |
| ENSECAT00000010924 | ENSECAG00000010394 | LOC100054867 | 2.29 |
| ENSECAT00000010956 | ENSECAG00000010284 | LOC100057650 | 1.4 |
| ENSECAT00000010964 | ENSECAG00000010656 | HBEGF | 3.18 |
| ENSECAT00000011003 | ENSECAG00000010420 | LOC100070138 | 1.49 |
| ENSECAT00000011049 | ENSECAG00000010324 | | 2.17 |
| ENSECAT00000011058 | ENSECAG00000010613 | KLF4 | 1.72 |
| ENSECAT00000011061 | ENSECAG00000010212 | SLK | 1.23 |
| ENSECAT00000011074 | ENSECAG00000010663 | LOC100057606 | 1.73 |
| ENSECAT00000011095 | ENSECAG00000010267 | B4Y7K2_HORSE | 3.08 |
| ENSECAT00000011116 | ENSECAG00000010808 | LOC100053123 | 1.84 |
| ENSECAT00000011133 | ENSECAG00000010815 | | 1.59 |
| ENSECAT00000011152 | ENSECAG00000010818 | LOC100062890 | 3.47 |
| ENSECAT00000011157 | ENSECAG00000010715 | LOC100067359 | 0.94 |
| ENSECAT00000011188 | ENSECAG00000010681 | SAMD4A | 0.9 |
| ENSECAT00000011189 | ENSECAG00000009792 | LOC100072764 | 2.11 |
| ENSECAT00000011194 | ENSECAG00000010839 | LOC100147209 | 1.43 |
| ENSECAT00000011206 | ENSECAG00000010817 | CTTNBP2NL | 1.93 |
| ENSECAT00000011210 | ENSECAG00000010847 | LOC100071026 | 1.37 |
| ENSECAT00000011218 | ENSECAG00000010521 | LOC100051655 | 1.42 |
| ENSECAT00000011264 | ENSECAG00000010493 | LOC100053982 | 1.42 |
| ENSECAT00000011271 | ENSECAG00000010914 | CHSY1 | 2.54 |
| ENSECAT00000011300 | ENSECAG00000010667 | ERG | 1.18 |
| ENSECAT00000011401 | ENSECAG00000011086 | LOC100062982 | 1.54 |
| ENSECAT00000011415 | ENSECAG00000011048 | LOC100058900 | 2.44 |
| ENSECAT00000011460 | ENSECAG00000011078 | LOC100051444 | 3.29 |
| ENSECAT00000011461 | ENSECAG00000011107 | | 2.99 |
| ENSECAT00000011464 | ENSECAG00000010727 | LOC100068150 | 2 |
| ENSECAT00000011485 | ENSECAG00000011055 | LOC100057181 | 1.65 |
| ENSECAT00000011498 | ENSECAG00000010897 | O62767_HORSE | 1.44 |
| ENSECAT00000011501 | ENSECAG00000010772 | LOC100053845 | 1.43 |
| ENSECAT00000011504 | ENSECAG00000010810 | LOC100072661 | 2.99 |
| ENSECAT00000011519 | ENSECAG00000011145 | LOC100053208 | 2.24 |
| ENSECAT00000011552 | ENSECAG00000011135 | LOC100057984 | 1.88 |
| ENSECAT00000011553 | ENSECAG00000010990 | A8DS43_HORSE | 1.86 |
| ENSECAT00000011640 | ENSECAG00000011009 | STARD13 | 1.71 |
| ENSECAT00000011669 | ENSECAG00000011163 | FCHSD2 | 1.01 |
| ENSECAT00000011693 | ENSECAG00000011257 | LOC100060923 | 2.48 |
| ENSECAT00000011754 | ENSECAG00000011072 | LOC100071653 | 1.49 |
| ENSECAT00000011801 | ENSECAG00000011391 | LOC100057820 | 1.49 |
| ENSECAT00000011822 | ENSECAG00000011198 | LOC100072355 | 2.22 |
| ENSECAT00000011823 | ENSECAG00000011263 | LOC100061904 | 1.37 |
| ENSECAT00000011849 | ENSECAG00000011380 | TACC3 | 1.95 |
| ENSECAT00000011862 | ENSECAG00000010867 | FUBP1 | 1.64 |
| ENSECAT00000011865 | ENSECAG00000011433 | TET2 | 2.47 |
| ENSECAT00000011926 | ENSECAG00000010919 | MYO9B | 1.84 |
| ENSECAT00000011931 | ENSECAG00000011486 | LOC100050849 | 5.62 |
| ENSECAT00000011948 | ENSECAG00000011003 | PDE4B | 1.94 |
| ENSECAT00000011957 | ENSECAG00000011309 | ARHGAP21 | 1.09 |
| ENSECAT00000011959 | ENSECAG00000011132 | LOC100064168 | 1.53 |
| ENSECAT00000011969 | ENSECAG00000010918 | Q5J3Q6_HORSE | 3.26 |
| ENSECAT00000011993 | ENSECAG00000011296 | LOC100070959 | 0.87 |
| ENSECAT00000012048 | ENSECAG00000011671 | TGFB1_HORSE | 1.67 |
| ENSECAT00000012055 | ENSECAG00000011512 | | 1.62 |
| ENSECAT00000012062 | ENSECAG00000011528 | LOC100053880 | 1.89 |
| ENSECAT00000012091 | ENSECAG00000011733 | RNASE6 | 1.62 |
| ENSECAT00000012141 | ENSECAG00000011697 | LOC100064943 | 3.12 |
| ENSECAT00000012144 | ENSECAG00000011711 | LOC100053211 | 1.25 |
| ENSECAT00000012155 | ENSECAG00000011496 | Q6QNF5_HORSE | 4.64 |
| ENSECAT00000012216 | ENSECAG00000009700 | ANK3 | 4.03 |
| ENSECAT00000012220 | ENSECAG00000010052 | LOC100071760 | 1.4 |
| ENSECAT00000012241 | ENSECAG00000011827 | SBNO2 | 3.38 |
| ENSECAT00000012281 | ENSECAG00000011647 | GMIP | 0.93 |
| ENSECAT00000012300 | ENSECAG00000011902 | LOC100054073 | 1.16 |
| ENSECAT00000012325 | ENSECAG00000011553 | LOC100067716 | 0.96 |
| ENSECAT00000012340 | ENSECAG00000010845 | LOC100063323 | 1.63 |
| ENSECAT00000012350 | ENSECAG00000011895 | ILT11B | 2.23 |
| ENSECAT00000012354 | ENSECAG00000011966 | ZNF217 | 2.91 |
| ENSECAT00000012373 | ENSECAG00000011928 | LRRC8C | 2.08 |
| ENSECAT00000012392 | ENSECAG00000011890 | ARHGAP30 | 1.25 |
| ENSECAT00000012403 | ENSECAG00000011858 | UBE2V1 | 2.41 |
| ENSECAT00000012430 | ENSECAG00000011767 | KRI1 | 0.77 |
| ENSECAT00000012434 | ENSECAG00000011938 | LOC100052301 | 1.17 |
| ENSECAT00000012482 | ENSECAG00000011471 | AXL | 3.17 |
| ENSECAT00000012505 | ENSECAG00000012104 | Q29XY3_HORSE | 3.16 |
| ENSECAT00000012557 | ENSECAG00000012148 | C15orf48 | 3.93 |
| ENSECAT00000012559 | ENSECAG00000012134 | LOC100063632 | 1.08 |
| ENSECAT00000012594 | ENSECAG00000012179 | B7X6D7_HORSE | 3.49 |
| ENSECAT00000012596 | ENSECAG00000011830 | LOC100066965 | 1.3 |
| ENSECAT00000012602 | ENSECAG00000011939 | CLIP2 | 0.8 |
| ENSECAT00000012630 | ENSECAG00000011746 | LOC100065793 | 4.09 |
| ENSECAT00000012715 | ENSECAG00000012173 | LOC100062426 | 0.89 |
| ENSECAT00000012721 | ENSECAG00000011621 | NAT10 | 1 |
| ENSECAT00000012864 | ENSECAG00000012338 | LOC100065914 | 1.78 |
| ENSECAT00000012875 | ENSECAG00000011935 | SMURF2 | 1.13 |
| ENSECAT00000012913 | ENSECAG00000012140 | LOC100053554 | 0.77 |
| ENSECAT00000012927 | ENSECAG00000011995 | LOC100054030 | 1.75 |
| ENSECAT00000012931 | ENSECAG00000012280 | LOC100061779 | 1.33 |
| ENSECAT00000012942 | ENSECAG00000012476 | LOC100056892 | 2.73 |
| ENSECAT00000012986 | ENSECAG00000012229 | LOC100068894 | 4.68 |
| ENSECAT00000013011 | ENSECAG00000012526 | LOC100055586 | 0.82 |
| ENSECAT00000013018 | ENSECAG00000012584 | GNG2 | 1.44 |
| ENSECAT00000013231 | ENSECAG00000012481 | LOC100055840 | 0.95 |
| ENSECAT00000013285 | ENSECAG00000012738 | LOC100070195 | 3.32 |
| ENSECAT00000013305 | ENSECAG00000012639 | NOP2 | 1.66 |
| ENSECAT00000013317 | ENSECAG00000012479 | LARP4 | 1.4 |
| ENSECAT00000013323 | ENSECAG00000012776 | RLF | 1.09 |
| ENSECAT00000013338 | ENSECAG00000011917 | DOCK8 | 1.14 |
| ENSECAT00000013376 | ENSECAG00000012685 | Q206K2_HORSE | 1.66 |
| ENSECAT00000013395 | ENSECAG00000012858 | LOC100051189 | 2.88 |
| ENSECAT00000013416 | ENSECAG00000012701 | LOC100067887 | 0.96 |
| ENSECAT00000013437 | ENSECAG00000012646 | SYNJ2 | 2.24 |
| ENSECAT00000013490 | ENSECAG00000011972 | UBR1 | 1.62 |
| ENSECAT00000013491 | N/A | N/A | 1.19 |
| ENSECAT00000013503 | ENSECAG00000012727 | TNFAIP1 | 1.39 |
| ENSECAT00000013536 | ENSECAG00000013046 | ZNF295 | 2.57 |
| ENSECAT00000013598 | ENSECAG00000012659 | Q2LGF2_HORSE | 0.97 |
| ENSECAT00000013603 | ENSECAG00000012960 | LOC100058093 | 3.08 |
| ENSECAT00000013604 | ENSECAG00000013073 | ZBTB10 | 1.22 |
| ENSECAT00000013620 | ENSECAG00000012558 | LOC100061692 | 1.28 |
| ENSECAT00000013629 | ENSECAG00000012876 | LOC100069303 | 2.17 |
| ENSECAT00000013636 | ENSECAG00000011994 | CYFIP2 | 1.58 |
| ENSECAT00000013680 | ENSECAG00000013124 | BCL3 | 4.16 |
| ENSECAT00000013715 | ENSECAG00000013153 | LOC100062273 | 3.01 |
| ENSECAT00000013742 | ENSECAG00000013239 | LOC100067188 | 2.21 |
| ENSECAT00000013743 | ENSECAG00000012166 | LRP1 | 1.37 |
| ENSECAT00000013768 | ENSECAG00000012941 | JMJD1C | 1.71 |
| ENSECAT00000013833 | ENSECAG00000012445 | CHD1 | 2.29 |
| ENSECAT00000013936 | ENSECAG00000013345 | LOC100053868 | 1.66 |
| ENSECAT00000013951 | ENSECAG00000013300 | B1AB90_HORSE | 2.55 |
| ENSECAT00000013977 | ENSECAG00000013359 | LOC100066843 | 1.21 |
| ENSECAT00000014006 | ENSECAG00000013035 | ENPP1 | 1.15 |
| ENSECAT00000014037 | ENSECAG00000012032 | A8E0G5_HORSE | 1.33 |
| ENSECAT00000014042 | ENSECAG00000013035 | ENPP1 | 1.37 |
| ENSECAT00000014065 | ENSECAG00000013035 | ENPP1 | 1.94 |
| ENSECAT00000014089 | ENSECAG00000013081 | A8HDR2_HORSE | 2.67 |
| ENSECAT00000014158 | ENSECAG00000013094 | LOC100056511 | 1.23 |
| ENSECAT00000014165 | ENSECAG00000013525 | LOC100065093 | 2.68 |
| ENSECAT00000014166 | ENSECAG00000013443 | AKNA | 1.14 |
| ENSECAT00000014176 | ENSECAG00000009706 | LOC100070242 | 1.41 |
| ENSECAT00000014185 | ENSECAG00000012032 | A8E0G5_HORSE | 0.69 |
| ENSECAT00000014206 | ENSECAG00000013478 | LOC100052080 | 1.25 |
| ENSECAT00000014225 | ENSECAG00000013534 | LOC100058745 | 2.72 |
| ENSECAT00000014268 | ENSECAG00000013457 | CSF3R | 2.21 |
| ENSECAT00000014276 | ENSECAG00000013004 | ADCY4 | 1.61 |
| ENSECAT00000014291 | ENSECAG00000013624 | LOC100064344 | 1.34 |
| ENSECAT00000014297 | ENSECAG00000013635 | LOC100059103 | 2.85 |
| ENSECAT00000014315 | ENSECAG00000013691 | LOC100054579 | 1.41 |
| ENSECAT00000014335 | ENSECAG00000013404 | CAND1 | 1.2 |
| ENSECAT00000014366 | ENSECAG00000013778 | LOC100054913 | 1.51 |
| ENSECAT00000014392 | ENSECAG00000013110 | LOC100051975 | 2.06 |
| ENSECAT00000014409 | ENSECAG00000013055 | LOC100067695 | 2.14 |
| ENSECAT00000014415 | ENSECAG00000012862 | LYN | 1.6 |
| ENSECAT00000014451 | ENSECAG00000013055 | LOC100067695 | 3.52 |
| ENSECAT00000014453 | Known psuedogene | | 1.4 |
| ENSECAT00000014461 | ENSECAG00000013757 | ERO1L | 0.9 |
| ENSECAT00000014465 | ENSECAG00000013731 | LOC100054071 | 3.32 |
| ENSECAT00000014480 | ENSECAG00000013745 | LOC100068610 | 2.51 |
| ENSECAT00000014496 | ENSECAG00000013283 | LOC100072996 | 1.38 |
| ENSECAT00000014530 | ENSECAG00000013939 | ZFHX2 | 1.82 |
| ENSECAT00000014554 | ENSECAG00000013542 | LOC100069901 | 1.03 |
| ENSECAT00000014573 | ENSECAG00000013711 | DOT1L | 1.75 |
| ENSECAT00000014621 | ENSECAG00000013711 | DOT1L | 2.38 |
| ENSECAT00000014653 | ENSECAG00000013814 | LOC100071434 | 2.49 |
| ENSECAT00000014661 | ENSECAG00000013636 | | 1.99 |
| ENSECAT00000014665 | ENSECAG00000014017 | LOC100071872 | 1.77 |
| ENSECAT00000014702 | ENSECAG00000013909 | LOC100072571 | 1.24 |
| ENSECAT00000014707 | ENSECAG00000013901 | ANKRD12 | 1.73 |
| ENSECAT00000014743 | ENSECAG00000014019 | LOC100054052 | 1.68 |
| ENSECAT00000014762 | ENSECAG00000013728 | MFSD2A | 5.16 |
| ENSECAT00000014776 | ENSECAG00000013952 | CADM4 | 1.24 |
| ENSECAT00000014779 | ENSECAG00000014094 | LOC100073020 | 2.26 |
| ENSECAT00000014786 | ENSECAG00000013973 | TRAF3 | 1.23 |
| ENSECAT00000014799 | ENSECAG00000013779 | AFAP1 | 1.01 |
| ENSECAT00000014856 | ENSECAG00000014260 | LOC100051632 | 4.91 |
| ENSECAT00000014900 | ENSECAG00000014071 | LOC100055424 | 2.35 |
| ENSECAT00000014937 | ENSECAG00000014259 | TIMP1_HORSE | 4.8 |
| ENSECAT00000014939 | ENSECAG00000014232 | NR4A2 | 5.29 |
| ENSECAT00000014965 | ENSECAG00000014338 | LOC100072574 | 5.71 |
| ENSECAT00000014969 | ENSECAG00000013883 | LOC100058207 | 4.61 |
| ENSECAT00000014994 | ENSECAG00000014364 | LOC100062679 | 1.4 |
| ENSECAT00000015017 | ENSECAG00000014087 | LOC100072549 | 1.22 |
| ENSECAT00000015084 | ENSECAG00000014479 | LOC100058493 | 2.62 |
| ENSECAT00000015133 | ENSECAG00000014300 | CBLB | 1.41 |
| ENSECAT00000015171 | ENSECAG00000014357 | LOC100052876 | 1.23 |
| ENSECAT00000015196 | ENSECAG00000014563 | TWISTNB | 1.28 |
| ENSECAT00000015220 | ENSECAG00000013759 | ARHGEF2 | 1.32 |
| ENSECAT00000015222 | ENSECAG00000014601 | GADD45A | 3.66 |
| ENSECAT00000015229 | ENSECAG00000014370 | TRIM55 | 1.45 |
| ENSECAT00000015243 | ENSECAG00000014112 | LOC100064887 | 2.01 |
| ENSECAT00000015253 | ENSECAG00000014489 | CNN1 | 1.92 |
| ENSECAT00000015325 | ENSECAG00000014142 | LOC100063737 | 1.5 |
| ENSECAT00000015334 | ENSECAG00000014701 | LOC100060199 | 1.54 |
| ENSECAT00000015336 | ENSECAG00000014702 | LOC100063991 | 1.85 |
| ENSECAT00000015344 | ENSECAG00000014268 | KDM5B | 1.5 |
| ENSECAT00000015402 | ENSECAG00000014699 | NUAK2 | 2.83 |
| ENSECAT00000015485 | ENSECAG00000014422 | Q6R757_HORSE | 1.48 |
| ENSECAT00000015486 | ENSECAG00000014770 | CDR2 | 2.23 |
| ENSECAT00000015516 | ENSECAG00000014787 | RCAN1 | 3.29 |
| ENSECAT00000015549 | ENSECAG00000014780 | XBP1 | 2.1 |
| ENSECAT00000015551 | ENSECAG00000014838 | | 1.38 |
| ENSECAT00000015567 | ENSECAG00000014755 | LOC100069400 | 1.77 |
| ENSECAT00000015608 | ENSECAG00000014800 | MAP3K14 | 1.83 |
| ENSECAT00000015633 | ENSECAG00000014916 | LOC100065885 | 3.23 |
| ENSECAT00000015648 | ENSECAG00000014952 | LOC100056724 | 1.06 |
| ENSECAT00000015683 | ENSECAG00000014594 | LOC100068622 | 2.22 |
| ENSECAT00000015695 | ENSECAG00000015009 | UGDH | 2.68 |
| ENSECAT00000015718 | ENSECAG00000015000 | LOC100147132 | 1.19 |
| ENSECAT00000015773 | ENSECAG00000014968 | JMY | 1.1 |
| ENSECAT00000015842 | ENSECAG00000015133 | LOC100060644 | 1.6 |
| ENSECAT00000015857 | ENSECAG00000014874 | LOC100052186 | 1.09 |
| ENSECAT00000015862 | ENSECAG00000014676 | PREX1 | 1.27 |
| ENSECAT00000015869 | ENSECAG00000015069 | LOC100059809 | 1.75 |
| ENSECAT00000015883 | ENSECAG00000014676 | PREX1 | 1.36 |
| ENSECAT00000015892 | Known psuedogene | | 1.86 |
| ENSECAT00000015906 | Known psuedogene | | 2.16 |
| ENSECAT00000015939 | ENSECAG00000014427 | LOC100067268 | 2.58 |
| ENSECAT00000015943 | Known psuedogene | | 1.35 |
| ENSECAT00000015965 | ENSECAG00000015134 | PELI2 | 2.06 |
| ENSECAT00000015995 | ENSECAG00000015275 | MT1B_HORSE | 1.58 |
| ENSECAT00000016001 | ENSECAG00000014760 | RRBP1 | 1.28 |
| ENSECAT00000016047 | ENSECAG00000015128 | LOC100053297 | 2.08 |
| ENSECAT00000016048 | ENSECAG00000015287 | LOC100069912 | 1.3 |
| ENSECAT00000016053 | ENSECAG00000015280 | LOC100054386 | 1.06 |
| ENSECAT00000016076 | ENSECAG00000015301 | LOC100060999 | 1.43 |
| ENSECAT00000016077 | ENSECAG00000015053 | Q6X9X5_HORSE | 1.5 |
| ENSECAT00000016114 | ENSECAG00000014833 | A0MLS6_HORSE | 1.16 |
| ENSECAT00000016120 | ENSECAG00000015428 | CMTM6 | 1.09 |
| ENSECAT00000016152 | ENSECAG00000015010 | LOC100070707 | 2.48 |
| ENSECAT00000016205 | ENSECAG00000014963 | PIK3C2A | 1.73 |
| ENSECAT00000016288 | ENSECAG00000015510 | Q8SPL1_HORSE | 2.2 |
| ENSECAT00000016309 | ENSECAG00000015042 | LOC100067894 | 1.47 |
| ENSECAT00000016467 | ENSECAG00000014690 | CAD | 1.31 |
| ENSECAT00000016547 | ENSECAG00000015413 | LOC100063073 | 4.16 |
| ENSECAT00000016577 | ENSECAG00000015813 | WHAMM | 1.03 |
| ENSECAT00000016602 | ENSECAG00000015460 | LOC100060842 | 2.34 |
| ENSECAT00000016636 | ENSECAG00000015369 | LOC100067227 | 1.46 |
| ENSECAT00000016664 | ENSECAG00000015336 | NUP98 | 1.08 |
| ENSECAT00000016695 | ENSECAG00000015762 | SNAPC4 | 1.61 |
| ENSECAT00000016709 | ENSECAG00000015440 | TBC1D9 | 3.01 |
| ENSECAT00000016720 | ENSECAG00000015952 | BDNF_HORSE | 2.68 |
| ENSECAT00000016723 | ENSECAG00000015740 | LOC100147601 | 1.83 |
| ENSECAT00000016824 | ENSECAG00000015865 | GLUL | 1.59 |
| ENSECAT00000016840 | ENSECAG00000015797 | LOC100058457 | 2.29 |
| ENSECAT00000016846 | ENSECAG00000015986 | LOC100071480 | 1.11 |
| ENSECAT00000016848 | ENSECAG00000015256 | LOC100070202 | 1.25 |
| ENSECAT00000016885 | ENSECAG00000015858 | NDEL1 | 2.06 |
| ENSECAT00000016943 | ENSECAG00000015597 | LOC100061166 | 1.9 |
| ENSECAT00000016973 | ENSECAG00000016126 | LOC100064945 | 2.64 |
| ENSECAT00000016977 | ENSECAG00000016134 | RAB11FIP1 | 3.13 |
| ENSECAT00000017004 | ENSECAG00000016087 | LOC100057301 | 2.01 |
| ENSECAT00000017020 | ENSECAG00000015006 | Q6XA02_HORSE | 2.03 |
| ENSECAT00000017032 | ENSECAG00000016002 | FBXO32 | 1.69 |
| ENSECAT00000017038 | ENSECAG00000016099 | BMP2K | 1.41 |
| ENSECAT00000017044 | ENSECAG00000015894 | ANGPT2 | 3.31 |
| ENSECAT00000017119 | ENSECAG00000015801 | LOC100057807 | 1.13 |
| ENSECAT00000017144 | ENSECAG00000016324 | CD93 | 2.08 |
| ENSECAT00000017186 | ENSECAG00000016284 | LOC100062585 | 1.06 |
| ENSECAT00000017188 | ENSECAG00000016116 | USP36 | 2.48 |
| ENSECAT00000017238 | ENSECAG00000016168 | LOC100071457 | 1.22 |
| ENSECAT00000017244 | ENSECAG00000015794 | ANXA1_HORSE | 1.55 |
| ENSECAT00000017343 | ENSECAG00000016535 | LOC100058421 | 4.23 |
| ENSECAT00000017346 | ENSECAG00000016449 | ILT11A | 1.97 |
| ENSECAT00000017379 | ENSECAG00000016383 | USP53 | 1.63 |
| ENSECAT00000017404 | ENSECAG00000016332 | PIGO | 1.48 |
| ENSECAT00000017430 | ENSECAG00000016297 | SUPV3L1 | 1.2 |
| ENSECAT00000017447 | ENSECAG00000016131 | FRMD4B | 4.51 |
| ENSECAT00000017451 | ENSECAG00000016330 | D1MH71_HORSE | 1.65 |
| ENSECAT00000017536 | ENSECAG00000016532 | LOC100058148 | 1.06 |
| ENSECAT00000017545 | ENSECAG00000016610 | LOC100073098 | 1.25 |
| ENSECAT00000017563 | ENSECAG00000016577 | LOC100058590 | 2.72 |
| ENSECAT00000017575 | ENSECAG00000016735 | A7LP08_HORSE | 2.01 |
| ENSECAT00000017618 | ENSECAG00000016772 | NANS | 1.27 |
| ENSECAT00000017629 | ENSECAG00000016339 | Q8HZM8_HORSE | 3.26 |
| ENSECAT00000017631 | ENSECAG00000016738 | LOC100068623 | 1.17 |
| ENSECAT00000017645 | ENSECAG00000016394 | Q9TV71_HORSE | 1.93 |
| ENSECAT00000017662 | ENSECAG00000016265 | STK10 | 1 |
| ENSECAT00000017703 | ENSECAG00000016721 | LOC100058229 | 5.39 |
| ENSECAT00000017731 | ENSECAG00000016661 | LOC100061353 | 2.15 |
| ENSECAT00000017760 | ENSECAG00000016658 | LOC100058405 | 4.12 |
| ENSECAT00000017789 | ENSECAG00000016667 | | 1.63 |
| ENSECAT00000017797 | ENSECAG00000016899 | LOC100147549 | 0.85 |
| ENSECAT00000017809 | ENSECAG00000016644 | LOC100061344 | 1.9 |
| ENSECAT00000017838 | ENSECAG00000016866 | LOC100054291 | 2.17 |
| ENSECAT00000017840 | ENSECAG00000016926 | LOC100055527 | 0.89 |
| ENSECAT00000017859 | ENSECAG00000016508 | LOC100068725 | 1.72 |
| ENSECAT00000017863 | ENSECAG00000016548 | WDR46 | 0.99 |
| ENSECAT00000017872 | ENSECAG00000016862 | LOC100057608 | 2.24 |
| ENSECAT00000017880 | ENSECAG00000016948 | LOC100054462 | 0.96 |
| ENSECAT00000017985 | ENSECAG00000017104 | ERRFI1 | 3.41 |
| ENSECAT00000018086 | ENSECAG00000017157 | LOC100055430 | 2.97 |
| ENSECAT00000018108 | ENSECAG00000016637 | LOC100060271 | 1.02 |
| ENSECAT00000018124 | ENSECAG00000017223 | LOC100053597 | 1.77 |
| ENSECAT00000018126 | ENSECAG00000017203 | LOC100053122 | 6.04 |
| ENSECAT00000018136 | ENSECAG00000016983 | LOC100071482 | 4.23 |
| ENSECAT00000018153 | ENSECAG00000016922 | LOC100050864 | 1.52 |
| ENSECAT00000018163 | ENSECAG00000017046 | LOC100068915 | 1.23 |
| ENSECAT00000018216 | ENSECAG00000016829 | ABTB2 | 1.33 |
| ENSECAT00000018252 | ENSECAG00000017319 | LOC100066114 | 1.44 |
| ENSECAT00000018255 | ENSECAG00000016439 | Q6X9Y0_HORSE | 1.6 |
| ENSECAT00000018286 | ENSECAG00000016998 | TNR5_HORSE | 1.77 |
| ENSECAT00000018321 | ENSECAG00000016782 | MYO1F | 1.58 |
| ENSECAT00000018336 | ENSECAG00000017179 | PHC2 | 2.04 |
| ENSECAT00000018405 | ENSECAG00000017436 | LOC100054285 | 6.56 |
| ENSECAT00000018418 | ENSECAG00000016782 | MYO1F | 1.78 |
| ENSECAT00000018424 | ENSECAG00000017457 | LOC100059499 | 2 |
| ENSECAT00000018477 | ENSECAG00000017253 | LOC100050392 | 1.08 |
| ENSECAT00000018521 | ENSECAG00000017193 | LOC100058365 | 1.08 |
| ENSECAT00000018531 | ENSECAG00000016931 | MLL5 | 1.3 |
| ENSECAT00000018547 | ENSECAG00000017295 | LOC100051043 | 2.38 |
| ENSECAT00000018581 | ENSECAG00000016238 | RPS6KA1 | 1.85 |
| ENSECAT00000018624 | ENSECAG00000017419 | LOC100053990 | 3.25 |
| ENSECAT00000018682 | ENSECAG00000017724 | LOC100062972 | 1.73 |
| ENSECAT00000018687 | ENSECAG00000017676 | Q6RF65_HORSE | 1.89 |
| ENSECAT00000018723 | ENSECAG00000017657 | LOC100071081 | 3.31 |
| ENSECAT00000018742 | ENSECAG00000016912 | LOC100066562 | 1.92 |
| ENSECAT00000018749 | ENSECAG00000017715 | CSF1 | 3.65 |
| ENSECAT00000018803 | ENSECAG00000017644 | CFP | 2.09 |
| ENSECAT00000018805 | ENSECAG00000017405 | PPP1R13B | 2.1 |
| ENSECAT00000018836 | ENSECAG00000017839 | | 2.02 |
| ENSECAT00000018852 | ENSECAG00000017346 | LOC100056731 | 1.95 |
| ENSECAT00000018865 | ENSECAG00000017574 | LOC100063651 | 3.06 |
| ENSECAT00000018899 | ENSECAG00000016824 | NRP2 | 1.28 |
| ENSECAT00000018999 | ENSECAG00000017794 | LOC100063253 | 2.47 |
| ENSECAT00000019009 | ENSECAG00000016927 | LOC100051725 | 3.15 |
| ENSECAT00000019012 | ENSECAG00000017901 | LOC100062490 | 1.99 |
| ENSECAT00000019013 | ENSECAG00000018028 | TLR2_HORSE | 3.42 |
| ENSECAT00000019016 | ENSECAG00000017984 | Q8MKD9_HORSE | 2.01 |
| ENSECAT00000019021 | ENSECAG00000018014 | LOC100058079 | 1.71 |
| ENSECAT00000019034 | ENSECAG00000017433 | Q6X9X2_HORSE | 3.31 |
| ENSECAT00000019051 | ENSECAG00000017787 | LOC100050536 | 2.67 |
| ENSECAT00000019056 | ENSECAG00000018002 | LOC100065680 | 5.42 |
| ENSECAT00000019058 | ENSECAG00000018056 | LOC100056246 | 1.44 |
| ENSECAT00000019079 | ENSECAG00000017400 | SMURF1 | 2.04 |
| ENSECAT00000019226 | ENSECAG00000018072 | LOC100069924 | 1.33 |
| ENSECAT00000019244 | ENSECAG00000017968 | SLC41A1 | 1.46 |
| ENSECAT00000019270 | ENSECAG00000017970 | LOC100067445 | 2.17 |
| ENSECAT00000019314 | ENSECAG00000017672 | MAST4 | 2.06 |
| ENSECAT00000019370 | ENSECAG00000017934 | LOC100070527 | 1.49 |
| ENSECAT00000019462 | ENSECAG00000018113 | LYSC2_HORSE | 1.92 |
| ENSECAT00000019475 | ENSECAG00000018406 | LOC100061442 | 1.59 |
| ENSECAT00000019513 | ENSECAG00000018012 | Q6DTK4_HORSE | 1.22 |
| ENSECAT00000019521 | ENSECAG00000018426 | ETF1 | 1.26 |
| ENSECAT00000019554 | ENSECAG00000018261 | LOC100058342 | 3.83 |
| ENSECAT00000019557 | ENSECAG00000018343 | LENG8 | 1.21 |
| ENSECAT00000019582 | ENSECAG00000018395 | LOC100051740 | 1.88 |
| ENSECAT00000019584 | ENSECAG00000018478 | LOC100055172 | 1.04 |
| ENSECAT00000019610 | ENSECAG00000018421 | LOC100061659 | 1.24 |
| ENSECAT00000019618 | ENSECAG00000018141 | LASS2 | 1.05 |
| ENSECAT00000019685 | ENSECAG00000018600 | ACTB_HORSE | 1.25 |
| ENSECAT00000019687 | ENSECAG00000018531 | LOC100050475 | 2.5 |
| ENSECAT00000019693 | ENSECAG00000018548 | LOC100060341 | 1.13 |
| ENSECAT00000019701 | ENSECAG00000018490 | LOC100067790 | 2.31 |
| ENSECAT00000019717 | ENSECAG00000018649 | LOC100052144 | 0.94 |
| ENSECAT00000019751 | ENSECAG00000018664 | LOC100058490 | 3.09 |
| ENSECAT00000019802 | ENSECAG00000017799 | MYO1G | 3 |
| ENSECAT00000019833 | ENSECAG00000018580 | LOC100067358 | 2.97 |
| ENSECAT00000019839 | ENSECAG00000018333 | EZR | 1.06 |
| ENSECAT00000019843 | ENSECAG00000018324 | LOC100072377 | 1.98 |
| ENSECAT00000019892 | ENSECAG00000018743 | LOC100063805 | 2.26 |
| ENSECAT00000019896 | ENSECAG00000017765 | LOC100054744 | 3.86 |
| ENSECAT00000019925 | ENSECAG00000018834 | LOC100060953 | 2.15 |
| ENSECAT00000019974 | ENSECAG00000018823 | LOC100054765 | 1.32 |
| ENSECAT00000019988 | ENSECAG00000018860 | LOC100063597 | 1.84 |
| ENSECAT00000019993 | ENSECAG00000018843 | LOC100059506 | 1 |
| ENSECAT00000020011 | ENSECAG00000018849 | LOC100070723 | 1.08 |
| ENSECAT00000020163 | ENSECAG00000018428 | LOC100062150 | 3.54 |
| ENSECAT00000020165 | ENSECAG00000018874 | LOC100050052 | 1.43 |
| ENSECAT00000020220 | ENSECAG00000019069 | LOC100060300 | 1.27 |
| ENSECAT00000020251 | ENSECAG00000019124 | LOC100055141 | 2.18 |
| ENSECAT00000020272 | ENSECAG00000018824 | MOSPD2 | 1.06 |
| ENSECAT00000020370 | ENSECAG00000018996 | LOC100051039 | 3.57 |
| ENSECAT00000020382 | ENSECAG00000019152 | KIAA0284 | 1.14 |
| ENSECAT00000020406 | ENSECAG00000019071 | LOC100050742 | 2.36 |
| ENSECAT00000020453 | ENSECAG00000019340 | ZNF800 | 1.36 |
| ENSECAT00000020461 | ENSECAG00000019345 | LOC100050779 | 1.76 |
| ENSECAT00000020485 | ENSECAG00000019065 | ABCG1 | 1.34 |
| ENSECAT00000020501 | ENSECAG00000018584 | LOC100066875 | 1.84 |
| ENSECAT00000020516 | ENSECAG00000019327 | LOC100051514 | 4.59 |
| ENSECAT00000020599 | ENSECAG00000018921 | LOC100067900 | 0.94 |
| ENSECAT00000020601 | ENSECAG00000019398 | LOC100051361 | 2.17 |
| ENSECAT00000020602 | ENSECAG00000019438 | OSMR | 4.49 |
| ENSECAT00000020642 | ENSECAG00000019115 | LOC100055312 | 1.49 |
| ENSECAT00000020669 | ENSECAG00000018845 | PTBP1 | 1.22 |
| ENSECAT00000020707 | ENSECAG00000019173 | LOC100063998 | 3.16 |
| ENSECAT00000020769 | ENSECAG00000019411 | HERC6 | 1.64 |
| ENSECAT00000020800 | ENSECAG00000019596 | LOC100065275 | 2.64 |
| ENSECAT00000020819 | ENSECAG00000019626 | LOC100059755 | 2.82 |
| ENSECAT00000020871 | ENSECAG00000019726 | | 1.28 |
| ENSECAT00000020917 | Known psuedogene | | 1.46 |
| ENSECAT00000020929 | ENSECAG00000019660 | Q8MKC9_HORSE | 3.84 |
| ENSECAT00000020973 | ENSECAG00000019747 | LOC100064714 | 1.88 |
| ENSECAT00000020979 | ENSECAG00000019083 | SORL1 | 1.78 |
| ENSECAT00000020991 | ENSECAG00000019594 | DENND2C | 1.86 |
| ENSECAT00000021004 | ENSECAG00000019554 | LOC100068435 | 0.92 |
| ENSECAT00000021005 | ENSECAG00000018924 | PTK2B | 2.49 |
| ENSECAT00000021013 | ENSECAG00000019083 | SORL1 | 1.81 |
| ENSECAT00000021023 | ENSECAG00000019830 | LOC100070605 | 1.84 |
| ENSECAT00000021030 | ENSECAG00000019506 | ARRDC3 | 2.03 |
| ENSECAT00000021068 | ENSECAG00000019895 | LOC100063749 | 2.71 |
| ENSECAT00000021091 | ENSECAG00000019795 | ARID5B | 3.43 |
| ENSECAT00000021133 | ENSECAG00000019900 | LOC100057603 | 1.06 |
| ENSECAT00000021144 | ENSECAG00000019821 | LOC100050643 | 1.45 |
| ENSECAT00000021202 | ENSECAG00000019914 | LOC100067767 | 1.65 |
| ENSECAT00000021269 | ENSECAG00000019855 | LMBRD2 | 1.29 |
| ENSECAT00000021327 | ENSECAG00000020066 | DNAJB1 | 2.26 |
| ENSECAT00000021336 | ENSECAG00000019557 | LOC100057389 | 2.16 |
| ENSECAT00000021348 | ENSECAG00000019885 | B2L2W7_HORSE | 1.37 |
| ENSECAT00000021352 | ENSECAG00000019750 | LOC100051700 | 1.54 |
| ENSECAT00000021367 | ENSECAG00000019750 | LOC100051700 | 1.5 |
| ENSECAT00000021368 | ENSECAG00000019593 | LOC100069984 | 1.85 |
| ENSECAT00000021431 | ENSECAG00000020201 | A8CWP2_HORSE | 1.82 |
| ENSECAT00000021435 | ENSECAG00000020180 | CREB5 | 1.99 |
| ENSECAT00000021441 | ENSECAG00000019617 | ATP8B4 | 1.56 |
| ENSECAT00000021443 | ENSECAG00000020162 | LOC100052234 | 1.37 |
| ENSECAT00000021448 | ENSECAG00000020122 | SELL | 1.63 |
| ENSECAT00000021502 | ENSECAG00000020240 | LOC100069511 | 1.48 |
| ENSECAT00000021514 | ENSECAG00000019897 | LOC100065131 | 1.35 |
| ENSECAT00000021519 | ENSECAG00000019875 | LOC100054613 | 2.3 |
| ENSECAT00000021536 | ENSECAG00000019918 | ANKS1A | 2.6 |
| ENSECAT00000021552 | ENSECAG00000020188 | LOC100058159 | 2 |
| ENSECAT00000021553 | ENSECAG00000019997 | LOC100071831 | 1.24 |
| ENSECAT00000021624 | ENSECAG00000018762 | Q0ZNY4_HORSE | 1.53 |
| ENSECAT00000021653 | ENSECAG00000020238 | LOC100063638 | 1.35 |
| ENSECAT00000021655 | ENSECAG00000018147 | AOX1 | 1.64 |
| ENSECAT00000021662 | ENSECAG00000020403 | LOC100052444 | 1.57 |
| ENSECAT00000021714 | ENSECAG00000019884 | LOC100064946 | 1.05 |
| ENSECAT00000021724 | ENSECAG00000020431 | LOC100073151 | 2.14 |
| ENSECAT00000021738 | ENSECAG00000020497 | LOC100064658 | 1.23 |
| ENSECAT00000021759 | ENSECAG00000020374 | LOC100070934 | 4.25 |
| ENSECAT00000021800 | ENSECAG00000019867 | DAPK1 | 1.59 |
| ENSECAT00000021808 | ENSECAG00000020535 | LOC100053417 | 2.07 |
| ENSECAT00000021819 | ENSECAG00000019476 | LOC100053070 | 1.87 |
| ENSECAT00000021863 | ENSECAG00000020218 | LOC100065490 | 1.16 |
| ENSECAT00000021892 | ENSECAG00000019932 | LOC100058771 | 1.35 |
| ENSECAT00000021895 | ENSECAG00000019880 | ADAMTS9 | 4.83 |
| ENSECAT00000021905 | ENSECAG00000020583 | Q865B2_HORSE | 1.92 |
| ENSECAT00000021922 | ENSECAG00000020639 | GLIS2 | 1.56 |
| ENSECAT00000021930 | ENSECAG00000020563 | PIM1 | 2.56 |
| ENSECAT00000021971 | ENSECAG00000020406 | PPP1R12A | 1.71 |
| ENSECAT00000021976 | ENSECAG00000020193 | LOC100072674 | 0.87 |
| ENSECAT00000021990 | ENSECAG00000020656 | LOC100072425 | 1.63 |
| ENSECAT00000021997 | ENSECAG00000020259 | Q8WNS8_HORSE | 1.09 |
| ENSECAT00000022005 | ENSECAG00000020299 | RGL1 | 1.62 |
| ENSECAT00000022009 | ENSECAG00000020740 | BTG1 | 1.27 |
| ENSECAT00000022043 | ENSECAG00000020657 | SYT7 | 1.5 |
| ENSECAT00000022116 | ENSECAG00000020653 | LOC100061060 | 1.25 |
| ENSECAT00000022117 | ENSECAG00000020720 | LOC100068959 | 3.57 |
| ENSECAT00000022222 | ENSECAG00000020664 | LOC100072289 | 1.54 |
| ENSECAT00000022293 | ENSECAG00000020816 | LOC100061326 | 1.56 |
| ENSECAT00000022294 | ENSECAG00000020125 | LOC100052938 | 1.63 |
| ENSECAT00000022299 | ENSECAG00000019533 | Q9XS91_HORSE | 3.47 |
| ENSECAT00000022322 | ENSECAG00000020898 | LOC100070664 | 2.36 |
| ENSECAT00000022353 | ENSECAG00000020699 | LOC100056021 | 1.34 |
| ENSECAT00000022372 | ENSECAG00000020971 | LOC100058747 | 5.13 |
| ENSECAT00000022374 | ENSECAG00000021025 | BEST3 | 1.09 |
| ENSECAT00000022451 | ENSECAG00000020792 | CAMTA1 | 2 |
| ENSECAT00000022482 | ENSECAG00000020289 | | 2.01 |
| ENSECAT00000022500 | ENSECAG00000021185 | LOC100052048 | 3.62 |
| ENSECAT00000022541 | ENSECAG00000021107 | LOC100051582 | 0.93 |
| ENSECAT00000022558 | ENSECAG00000020963 | LOC100071375 | 1.5 |
| ENSECAT00000022588 | ENSECAG00000020055 | PFKP | 1.38 |
| ENSECAT00000022641 | ENSECAG00000021028 | | 0.83 |
| ENSECAT00000022668 | ENSECAG00000021201 | Q95MB9_HORSE | 3.15 |
| ENSECAT00000022775 | ENSECAG00000021437 | FADD | 0.88 |
| ENSECAT00000022776 | ENSECAG00000021310 | LOC100050088 | 1.6 |
| ENSECAT00000022807 | ENSECAG00000021301 | MAN1A1 | 1.89 |
| ENSECAT00000022828 | ENSECAG00000021324 | LOC100070969 | 1.82 |
| ENSECAT00000022853 | ENSECAG00000021240 | SH3RF1 | 1.11 |
| ENSECAT00000022867 | ENSECAG00000021015 | ATP8B1 | 4.17 |
| ENSECAT00000022937 | ENSECAG00000021269 | Q9N1W3_HORSE | 2.96 |
| ENSECAT00000022942 | ENSECAG00000021220 | LOC100052081 | 1.29 |
| ENSECAT00000022944 | ENSECAG00000021497 | LOC100063668 | 3.67 |
| ENSECAT00000022965 | ENSECAG00000021598 | ZFAND2A | 2.71 |
| ENSECAT00000023038 | ENSECAG00000021613 | WWTR1 | 1.82 |
| ENSECAT00000023042 | ENSECAG00000021665 | SGMS2 | 2.84 |
| ENSECAT00000023128 | ENSECAG00000021484 | LOC100055623 | 3.07 |
| ENSECAT00000023132 | ENSECAG00000021627 | Q6SJP3_HORSE | 1.6 |
| ENSECAT00000023154 | ENSECAG00000021765 | LOC100071110 | 1.43 |
| ENSECAT00000023156 | ENSECAG00000021525 | IL4RA_HORSE | 3.12 |
| ENSECAT00000023202 | ENSECAG00000021773 | LOC100061223 | 1.59 |
| ENSECAT00000023206 | ENSECAG00000021806 | MUSTN1 | 2.21 |
| ENSECAT00000023210 | ENSECAG00000021804 | PELI1 | 1.49 |
| ENSECAT00000023244 | ENSECAG00000021122 | C6EN03_HORSE | 1.59 |
| ENSECAT00000023311 | ENSECAG00000021478 | EIF4A1 | 2.14 |
| ENSECAT00000023328 | ENSECAG00000021869 | LOC100054834 | 1.06 |
| ENSECAT00000023330 | ENSECAG00000021330 | Q95MA0_HORSE | 1.06 |
| ENSECAT00000023342 | ENSECAG00000021849 | TFPI | 1.52 |
| ENSECAT00000023380 | ENSECAG00000021529 | EZH2 | 2.14 |
| ENSECAT00000023396 | ENSECAG00000021603 | LOC100059274 | 1.69 |
| ENSECAT00000023419 | ENSECAG00000020933 | ITPR1 | 2.77 |
| ENSECAT00000023490 | ENSECAG00000021700 | Q8WMP2_HORSE | 1.31 |
| ENSECAT00000023507 | ENSECAG00000022059 | LOC100068097 | 4.62 |
| ENSECAT00000023523 | ENSECAG00000021841 | SH3BP2 | 1.34 |
| ENSECAT00000023526 | ENSECAG00000022074 | LOC100146873 | 1.4 |
| ENSECAT00000023532 | ENSECAG00000022078 | LOC100057594 | 1.57 |
| ENSECAT00000023533 | ENSECAG00000022074 | LOC100146873 | 1.32 |
| ENSECAT00000023542 | ENSECAG00000021720 | LOC100071115 | 1.4 |
| ENSECAT00000023552 | ENSECAG00000022037 | Q9N0M3_HORSE | 3 |
| ENSECAT00000023596 | ENSECAG00000021882 | LOC100056060 | 1.47 |
| ENSECAT00000023598 | ENSECAG00000022077 | ELF2 | 1.29 |
| ENSECAT00000023605 | ENSECAG00000022166 | LOC100063263 | 3.57 |
| ENSECAT00000023615 | ENSECAG00000021968 | LOC100051982 | 3.41 |
| ENSECAT00000023626 | ENSECAG00000022110 | ASXL1 | 1 |
| ENSECAT00000023663 | ENSECAG00000022006 | UAP1 | 2.04 |
| ENSECAT00000023693 | ENSECAG00000021679 | CDK17 | 1.55 |
| ENSECAT00000023710 | ENSECAG00000022172 | A5YBM8_HORSE | 2.5 |
| ENSECAT00000023756 | ENSECAG00000022135 | VNN1 | 1.24 |
| ENSECAT00000023782 | ENSECAG00000021996 | LOC100069402 | 1.11 |
| ENSECAT00000023862 | ENSECAG00000022376 | RAPH1 | 1.34 |
| ENSECAT00000023864 | ENSECAG00000022197 | Q3S2Z5_HORSE | 2.21 |
| ENSECAT00000023888 | ENSECAG00000022254 | TPM4_HORSE | 2.33 |
| ENSECAT00000023895 | ENSECAG00000022249 | LOC100071419 | 1.95 |
| ENSECAT00000023935 | ENSECAG00000022424 | TNFRSF12A | 4.04 |
| ENSECAT00000023956 | ENSECAG00000022462 | LOC100065052 | 1.32 |
| ENSECAT00000023958 | ENSECAG00000022301 | LOC100050911 | 1.19 |
| ENSECAT00000024009 | ENSECAG00000022309 | LOC100051976 | 1.3 |
| ENSECAT00000024013 | ENSECAG00000022162 | TBC1D1 | 1.15 |
| ENSECAT00000024026 | ENSECAG00000022495 | LOC100058988 | 1.48 |
| ENSECAT00000024069 | ENSECAG00000021742 | MYO5A | 4.02 |
| ENSECAT00000024194 | ENSECAG00000022676 | LOC100071468 | 2.14 |
| ENSECAT00000024281 | ENSECAG00000022741 | LOC100070148 | 1.31 |
| ENSECAT00000024394 | ENSECAG00000022859 | LOC100058651 | 1.34 |
| ENSECAT00000024431 | ENSECAG00000022690 | LOC100072405 | 1.23 |
| ENSECAT00000024458 | ENSECAG00000022742 | APBB1IP | 1.9 |
| ENSECAT00000024506 | ENSECAG00000021905 | HXK2_HORSE | 2.22 |
| ENSECAT00000024524 | ENSECAG00000021671 | | 0.9 |
| ENSECAT00000024527 | ENSECAG00000021905 | HXK2_HORSE | 2.14 |
| ENSECAT00000024561 | ENSECAG00000022999 | LOC100069444 | 2.5 |
| ENSECAT00000024624 | ENSECAG00000022948 | LOC100063088 | 2.34 |
| ENSECAT00000024666 | ENSECAG00000022527 | FMNL1 | 2.24 |
| ENSECAT00000024681 | ENSECAG00000023844 | LAMA2 | 1.96 |
| ENSECAT00000024708 | ENSECAG00000022933 | ELL2 | 2.29 |
| ENSECAT00000024742 | ENSECAG00000023011 | LOC100051086 | 2.9 |
| ENSECAT00000024840 | ENSECAG00000023216 | VNN2 | 1.47 |
| ENSECAT00000024991 | ENSECAG00000022405 | NOTCH1 | 0.92 |
| ENSECAT00000025016 | ENSECAG00000023381 | LOC100056907 | 1.37 |
| ENSECAT00000025030 | ENSECAG00000023009 | LOC100050663 | 1.66 |
| ENSECAT00000025033 | ENSECAG00000023260 | SLC7A6 | 1 |
| ENSECAT00000025038 | ENSECAG00000023363 | S26A2_HORSE | 1.71 |
| ENSECAT00000025048 | ENSECAG00000022653 | B0I1H0_HORSE | 1.26 |
| ENSECAT00000025148 | ENSECAG00000022873 | ATP8A1 | 0.94 |
| ENSECAT00000025158 | ENSECAG00000023399 | LOC100054996 | 3.07 |
| ENSECAT00000025181 | ENSECAG00000023460 | LOC100068620 | 0.96 |
| ENSECAT00000025225 | ENSECAG00000022880 | HIP1 | 0.72 |
| ENSECAT00000025226 | ENSECAG00000023420 | LOC100058000 | 1.15 |
| ENSECAT00000025287 | ENSECAG00000023172 | LOC100051333 | 1.12 |
| ENSECAT00000025292 | ENSECAG00000023546 | ETV6 | 1.73 |
| ENSECAT00000025294 | ENSECAG00000023516 | LOC100055372 | 1.5 |
| ENSECAT00000025307 | ENSECAG00000023358 | LOC100054274 | 1.96 |
| ENSECAT00000025368 | ENSECAG00000023685 | LOC100055536 | 2.09 |
| ENSECAT00000025373 | ENSECAG00000023642 | SPHK1 | 1 |
| ENSECAT00000025384 | ENSECAG00000023667 | LOC100062248 | 3.35 |
| ENSECAT00000025417 | ENSECAG00000023596 | LOC100065847 | 1.85 |
| ENSECAT00000025456 | ENSECAG00000023741 | EGR3 | 3.46 |
| ENSECAT00000025508 | ENSECAG00000023061 | LOC100056128 | 1.22 |
| ENSECAT00000025523 | ENSECAG00000023807 | LOC100060143 | 1.52 |
| ENSECAT00000025531 | ENSECAG00000023644 | PPAT | 1.53 |
| ENSECAT00000025590 | ENSECAG00000023253 | LOC100054721 | 2.04 |
| ENSECAT00000025612 | ENSECAG00000023859 | LOC100054048 | 2.35 |
| ENSECAT00000025661 | ENSECAG00000023430 | TRPM2 | 4.38 |
| ENSECAT00000025666 | ENSECAG00000023534 | CIITA | 3.2 |
| ENSECAT00000025674 | ENSECAG00000023430 | TRPM2 | 1.97 |
| ENSECAT00000025709 | ENSECAG00000023777 | LOC100071019 | 1.05 |
| ENSECAT00000025740 | ENSECAG00000023722 | LOC100061522 | 1.02 |
| ENSECAT00000025742 | ENSECAG00000023805 | LOC100060125 | 2.27 |
| ENSECAT00000025766 | ENSECAG00000023992 | LOC100056472 | 2.16 |
| ENSECAT00000025803 | ENSECAG00000024055 | LOC100067469 | 2.74 |
| ENSECAT00000025824 | ENSECAG00000024048 | TIPARP | 3.66 |
| ENSECAT00000025886 | ENSECAG00000024018 | LOC100054895 | 2.4 |
| ENSECAT00000025889 | ENSECAG00000024065 | LOC100068961 | 2.83 |
| ENSECAT00000025955 | ENSECAG00000024134 | LOC100053426 | 1.99 |
| ENSECAT00000025983 | ENSECAG00000024169 | | 1.47 |
| ENSECAT00000025985 | ENSECAG00000024139 | LONRF3 | 2.58 |
| ENSECAT00000026030 | ENSECAG00000024082 | ITGAX | 3.58 |
| ENSECAT00000026032 | ENSECAG00000024198 | SELI | 1.02 |
| ENSECAT00000026035 | ENSECAG00000024235 | TTC9 | 1.38 |
| ENSECAT00000026075 | ENSECAG00000024244 | CASP10 | 2.4 |
| ENSECAT00000026106 | ENSECAG00000024205 | Q9N1V8_HORSE | 1.48 |
| ENSECAT00000026110 | ENSECAG00000024304 | LOC100057911 | 1.28 |
| ENSECAT00000026111 | ENSECAG00000024236 | LOC100055680 | 1.44 |
| ENSECAT00000026117 | ENSECAG00000024136 | LOC100057764 | 2.45 |
| ENSECAT00000026189 | ENSECAG00000024369 | NIP7 | 1.23 |
| ENSECAT00000026267 | ENSECAG00000024246 | GIGYF2 | 1.74 |
| ENSECAT00000026270 | ENSECAG00000024186 | LOC100070369 | 3.69 |
| ENSECAT00000026279 | ENSECAG00000024428 | LOC100069445 | 2.25 |
| ENSECAT00000026339 | ENSECAG00000024457 | LOC100051397 | 1.93 |
| ENSECAT00000026380 | ENSECAG00000024448 | LOC100071234 | 3.38 |
| ENSECAT00000026397 | ENSECAG00000024512 | | 3.1 |
| ENSECAT00000026406 | ENSECAG00000024531 | Q9N0F0_HORSE | 1.77 |
| ENSECAT00000026410 | ENSECAG00000024512 | | 3.42 |
| ENSECAT00000026425 | ENSECAG00000024540 | HSPB7 | 0.79 |
| ENSECAT00000026447 | ENSECAG00000024352 | MYO1E | 1.4 |
| ENSECAT00000026474 | ENSECAG00000024297 | LOC100051633 | 1.69 |
| ENSECAT00000026482 | ENSECAG00000024543 | LOC100060766 | 1.99 |
| ENSECAT00000026496 | ENSECAG00000024591 | LOC100052536 | 1.02 |
| ENSECAT00000026610 | ENSECAG00000024699 | CYTH1 | 1.38 |
| ENSECAT00000026675 | ENSECAG00000024756 | LOC100051845 | 1.31 |
| ENSECAT00000026677 | ENSECAG00000024700 | LOC100060113 | 2.09 |
| ENSECAT00000026681 | ENSECAG00000024723 | LOC100058074 | 2.16 |
| ENSECAT00000026701 | ENSECAG00000024719 | LOC100053371 | 3.93 |
| ENSECAT00000026705 | ENSECAG00000024753 | LOC100064454 | 1.22 |
| ENSECAT00000026709 | ENSECAG00000024702 | LOC100058689 | 1.51 |
| ENSECAT00000026729 | ENSECAG00000024562 | Q8SPL6_HORSE | 2.2 |
| ENSECAT00000026804 | ENSECAG00000024623 | AT1A1_HORSE | 3.13 |
| ENSECAT00000026814 | ENSECAG00000024788 | LOC100064170 | 0.93 |
| ENSECAT00000026822 | ENSECAG00000024743 | LOC100063194 | 1.52 |
| ENSECAT00000026836 | ENSECAG00000024851 | EDNRB_HORSE | 1.21 |
| ENSECAT00000026841 | ENSECAG00000024847 | LOC100064387 | 3.03 |
| ENSECAT00000026843 | ENSECAG00000024761 | Q9BDH6_HORSE | 2.15 |
| ENSECAT00000026856 | ENSECAG00000024810 | PA24A_HORSE | 0.87 |
| ENSECAT00000026962 | Known psuedogene | | 1.82 |
| ENSECAT00000026964 | ENSECAG00000024988 | PARP14 | 1.56 |
| ENSECAT00000026966 | ENSECAG00000024993 | LOC100064066 | 2.16 |
| ENSECAT00000027002 | ENSECAG00000024991 | LOC100059292 | 0.85 |
| ENSECAT00000027037 | ENSECAG00000024979 | LOC100064655 | 1.59 |
| ENSECAT00000027038 | ENSECAG00000025036 | RSL1D1 | 1.14 |
| ENSECAT00000027063 | ENSECAG00000025055 | LOC100061723 | 3.25 |
| ENSECAT00000027073 | ENSECAG00000025059 | LOC100053057 | 1.06 |
| ENSECAT00000027094 | ENSECAG00000025087 | SEC23B | 3.05 |
| ENSECAT00000027105 | ENSECAG00000025107 | LOC100061847 | 1.03 |
| ENSECAT00000027125 | ENSECAG00000025124 | LOC100052424 | 1.18 |
| ENSECAT00000027138 | ENSECAG00000025129 | NDRG1 | 2.89 |
| ENSECAT00000028823 | ENSECAG00000009714 | RIN3 | 1.41 |
| ENSECAT00000028832 | ENSECAG00000026984 | LOC100051820 | 1.44 |
| ENSECAT00000028861 | ENSECAG00000012336 | ARHGAP26 | 2.06 |
| ENSECAT00000028870 | ENSECAG00000026816 | LOC100054472 | 1.86 |
| ENSECAT00000028895 | ENSECAG00000021067 | RAPGEF6 | 3.12 |
| ENSECAT00000029053 | ENSECAG00000020180 | CREB5 | 3.1 |
| ENSECAT00000029075 | ENSECAG00000026890 | LOC100059160 | 1.09 |
| ENSECAT00000029121 | ENSECAG00000011107 | | 0.87 |
| ENSECAT00000029140 | ENSECAG00000011966 | ZNF217 | 1.12 |

1. List of down-regulated DEGs in muscle tissue

| **Transcript name** | **Gene name (Ensembl)** | **Gene name** | **Average log2 ratio** |
| --- | --- | --- | --- |
| Cluster 1562 | Novel gene |  | -0.83 |
| Cluster 20688 | Novel gene |  | -3.02 |
| Cluster 37772 | Novel gene |  | -0.98 |
| ENSECAT00000000067 | ENSECAG00000000046 | LOC100062666 | -1.52 |
| ENSECAT00000000336 | ENSECAG00000000196 | MMP15 | -0.94 |
| ENSECAT00000000584 | ENSECAG00000000703 | LOC100051475 | -0.98 |
| ENSECAT00000000772 | ENSECAG00000000928 | | -1.32 |
| ENSECAT00000000826 | ENSECAG00000001012 | | -1.28 |
| ENSECAT00000001185 | ENSECAG00000001247 | LOC100060547 | -1 |
| ENSECAT00000001233 | ENSECAG00000001409 | LOC100056720 | -1.27 |
| ENSECAT00000001417 | ENSECAG00000000132 | Q5NU31_HORSE | -1.2 |
| ENSECAT00000001664 | ENSECAG00000000294 | ZMYM3 | -1.26 |
| ENSECAT00000002236 | ENSECAG00000000294 | ZMYM3 | -1.34 |
| ENSECAT00000002292 | ENSECAG00000000588 | LOC100059476 | -1.5 |
| ENSECAT00000002317 | ENSECAG00000000588 | LOC100059476 | -1.44 |
| ENSECAT00000002656 | ENSECAG00000001756 | LOC100058439 | -1.14 |
| ENSECAT00000002660 | ENSECAG00000001373 | LOC100066324 | -1.69 |
| ENSECAT00000002772 | ENSECAG00000002379 | RFX7 | -1.61 |
| ENSECAT00000002811 | ENSECAG00000002580 | LOC100053044 | -1.2 |
| ENSECAT00000002864 | ENSECAG00000002958 | LRRC4 | -1.4 |
| ENSECAT00000002934 | ENSECAG00000003067 | LOC100054863 | -1.38 |
| ENSECAT00000003027 | ENSECAG00000003167 | LOC100060243 | -0.29 |
| ENSECAT00000003584 | ENSECAG00000003704 | LOC100056093 | -1.36 |
| ENSECAT00000003690 | ENSECAG00000003822 | LOC100052291 | -0.96 |
| ENSECAT00000003850 | ENSECAG00000003990 | LOC100066512 | -0.92 |
| ENSECAT00000004086 | ENSECAG00000004107 | LOC100072811 | -1.36 |
| ENSECAT00000004400 | ENSECAG00000004450 | LOC100066548 | -1.45 |
| ENSECAT00000004614 | ENSECAG00000004658 | LOC100066889 | -2.32 |
| ENSECAT00000005073 | ENSECAG00000005106 | FIBIN_HORSE | -2.05 |
| ENSECAT00000005340 | ENSECAG00000004436 | LOC100070107 | -1.08 |
| ENSECAT00000005398 | ENSECAG00000005000 | PPM1K | -1.35 |
| ENSECAT00000005680 | ENSECAG00000005767 | CLDN12 | -1.23 |
| ENSECAT00000006283 | ENSECAG00000006273 | LOC100073095 | -1.33 |
| ENSECAT00000006305 | ENSECAG00000006300 | LOC100064013 | -1.12 |
| ENSECAT00000006446 | ENSECAG00000001653 | LOC100061669 | -1.53 |
| ENSECAT00000006678 | ENSECAG00000006568 | LOC100058244 | -0.81 |
| ENSECAT00000006708 | ENSECAG00000006267 | LOC100054466 | -1.5 |
| ENSECAT00000007072 | ENSECAG00000006669 | LOC100068672 | -1.24 |
| ENSECAT00000007216 | ENSECAG00000007133 | LOC100066008 | -1.15 |
| ENSECAT00000007261 | ENSECAG00000007220 | LOC100068636 | -1.01 |
| ENSECAT00000007273 | ENSECAG00000007233 | LOC100067766 | -0.88 |
| ENSECAT00000007287 | ENSECAG00000006609 | LOC100063598 | -1.6 |
| ENSECAT00000007575 | ENSECAG00000007095 | LOC100063506 | -0.82 |
| ENSECAT00000007606 | ENSECAG00000005968 | SCN4A | -0.96 |
| ENSECAT00000007771 | ENSECAG00000007698 | FZD4 | -1.5 |
| ENSECAT00000008115 | ENSECAG00000007315 | ACAP3 | -0.95 |
| ENSECAT00000008116 | ENSECAG00000007330 | ALS2CL | -1.15 |
| ENSECAT00000008213 | ENSECAG00000007948 | LOC100064028 | -1.56 |
| ENSECAT00000008403 | ENSECAG00000008151 | WDR73 | -1.07 |
| ENSECAT00000008482 | ENSECAG00000008346 | LOC100069761 | -1.05 |
| ENSECAT00000008512 | ENSECAG00000008381 | LOC100146166 | -3.04 |
| ENSECAT00000008599 | ENSECAG00000008407 | LOC100146441 | -1.64 |
| ENSECAT00000008619 | ENSECAG00000008410 | LOC100146704 | -1.3 |
| ENSECAT00000008755 | ENSECAG00000008518 | LOC100073038 | -0.9 |
| ENSECAT00000008790 | ENSECAG00000008093 | LOC100056322 | -1.28 |
| ENSECAT00000008938 | ENSECAG00000007446 | LOC100052236 | -0.93 |
| ENSECAT00000008974 | ENSECAG00000008059 | PDE4C | -1.15 |
| ENSECAT00000009030 | ENSECAG00000008489 | SYNRG | -0.93 |
| ENSECAT00000009068 | ENSECAG00000008842 | ASB10 | -0.96 |
| ENSECAT00000009105 | ENSECAG00000008270 | LOC100057538 | -1.29 |
| ENSECAT00000009106 | ENSECAG00000007984 | LOC100051842 | -1.31 |
| ENSECAT00000009352 | ENSECAG00000009135 | LOC100050350 | -1.53 |
| ENSECAT00000009369 | ENSECAG00000008342 | USP20 | -1.2 |
| ENSECAT00000009413 | ENSECAG00000009174 | ARSD | -1.77 |
| ENSECAT00000009467 | ENSECAG00000009287 | TRIM32 | -0.93 |
| ENSECAT00000009511 | ENSECAG00000008625 | MYBPC1 | -4.07 |
| ENSECAT00000009642 | ENSECAG00000009467 | LOC100061844 | -1.1 |
| ENSECAT00000009785 | ENSECAG00000009352 | OBSL1 | -1.19 |
| ENSECAT00000009788 | ENSECAG00000008985 | NBEA | -0.94 |
| ENSECAT00000009851 | ENSECAG00000009326 | MED1 | -1.08 |
| ENSECAT00000009856 | ENSECAG00000007647 | COL5A3 | -3.12 |
| ENSECAT00000009943 | ENSECAG00000009275 | AARSD1 | -2.51 |
| ENSECAT00000009969 | ENSECAG00000009768 | LOC100072697 | -1.54 |
| ENSECAT00000009974 | ENSECAG00000009453 | LOC100050834 | -1.18 |
| ENSECAT00000009977 | ENSECAG00000008405 | LOC100146115 | -1.62 |
| ENSECAT00000010011 | ENSECAG00000008405 | LOC100146115 | -0.51 |
| ENSECAT00000010133 | ENSECAG00000009159 | LOC100056548 | -1.51 |
| ENSECAT00000010210 | ENSECAG00000009793 | LOC100061511 | -1.65 |
| ENSECAT00000010243 | ENSECAG00000009707 | Q306F0_HORSE | -1.49 |
| ENSECAT00000010246 | ENSECAG00000009259 | LOC100070717 | -0.64 |
| ENSECAT00000010342 | ENSECAG00000009971 | LOC100056024 | -2.21 |
| ENSECAT00000010376 | ENSECAG00000008662 | LOC100054410 | -1.43 |
| ENSECAT00000010467 | ENSECAG00000009259 | LOC100070717 | -1.67 |
| ENSECAT00000010513 | ENSECAG00000009773 | ABCG2 | -0.99 |
| ENSECAT00000010647 | ENSECAG00000009999 | LOC100057973 | -1.21 |
| ENSECAT00000010663 | ENSECAG00000010035 | SCUBE2 | -1.66 |
| ENSECAT00000010674 | ENSECAG00000010259 | LOC100066246 | -1.24 |
| ENSECAT00000010966 | ENSECAG00000010506 | PLEKHH3 | -1.42 |
| ENSECAT00000011352 | ENSECAG00000011001 | LOC100058396 | -1.25 |
| ENSECAT00000011365 | ENSECAG00000010939 | LOC100052931 | -1.2 |
| ENSECAT00000011424 | ENSECAG00000011109 | GPR116 | -1.16 |
| ENSECAT00000011436 | ENSECAG00000010876 | RTKN | -1.48 |
| ENSECAT00000011583 | ENSECAG00000010869 | CAPN6 | -1.76 |
| ENSECAT00000011699 | ENSECAG00000010305 | ALBU_HORSE | -0.82 |
| ENSECAT00000011719 | ENSECAG00000010741 | LOC100072833 | -2.71 |
| ENSECAT00000011725 | ENSECAG00000010741 | LOC100072833 | -1.32 |
| ENSECAT00000011756 | ENSECAG00000010790 | COG6 | -7 |
| ENSECAT00000011821 | ENSECAG00000010790 | COG6 | -6.3 |
| ENSECAT00000011899 | ENSECAG00000011554 | LOC100068548 | -1.19 |
| ENSECAT00000011972 | ENSECAG00000011609 | LOC100051332 | -2.14 |
| ENSECAT00000011982 | ENSECAG00000011460 | LOC100062287 | -0.95 |
| ENSECAT00000012016 | ENSECAG00000010632 | LOC100069098 | -1.61 |
| ENSECAT00000012084 | ENSECAG00000011732 | LOC100055744 | -1.06 |
| ENSECAT00000012231 | ENSECAG00000011719 | MMRN1 | -1.06 |
| ENSECAT00000012290 | ENSECAG00000011582 | DLGAP4 | -11.07 |
| ENSECAT00000012311 | ENSECAG00000011762 | BTNL9 | -1.82 |
| ENSECAT00000012426 | ENSECAG00000011767 | KRI1 | -1.88 |
| ENSECAT00000012479 | ENSECAG00000011471 | AXL | -2.86 |
| ENSECAT00000012496 | ENSECAG00000011964 | IL27RA | -2.04 |
| ENSECAT00000012530 | ENSECAG00000012006 | BCL9L | -1.31 |
| ENSECAT00000012626 | ENSECAG00000011281 | LOC100055416 | -1.56 |
| ENSECAT00000012730 | ENSECAG00000012156 | LOC100070053 | -1.62 |
| ENSECAT00000012943 | ENSECAG00000012249 | LOC100054105 | -1.3 |
| ENSECAT00000013017 | ENSECAG00000011942 | Q95MA4_HORSE | -1.13 |
| ENSECAT00000013124 | ENSECAG00000012525 | PRR12 | -1.6 |
| ENSECAT00000013138 | ENSECAG00000012650 | KY | -1.86 |
| ENSECAT00000013198 | ENSECAG00000012351 | FUK | -1.12 |
| ENSECAT00000013214 | ENSECAG00000012758 | ZNF212 | -1.25 |
| ENSECAT00000013227 | ENSECAG00000012773 | CMPK2 | -1.11 |
| ENSECAT00000013276 | ENSECAG00000012654 | LOC100062997 | -0.85 |
| ENSECAT00000013331 | ENSECAG00000012841 | LOC100060091 | -1.05 |
| ENSECAT00000013352 | ENSECAG00000012739 | LOC100058612 | -1.22 |
| ENSECAT00000013420 | ENSECAG00000012547 | LOC100146163 | -1.27 |
| ENSECAT00000013449 | ENSECAG00000012582 | LOC100058665 | -4.2 |
| ENSECAT00000013451 | ENSECAG00000012812 | LOC100064295 | -1.29 |
| ENSECAT00000013481 | ENSECAG00000012302 | LOC100059541 | -1.79 |
| ENSECAT00000013493 | ENSECAG00000012454 | LOC100069884 | -1.5 |
| ENSECAT00000013516 | ENSECAG00000012302 | LOC100059541 | -2.83 |
| ENSECAT00000013523 | ENSECAG00000009361 | LOC100069057 | -2.83 |
| ENSECAT00000013524 | ENSECAG00000012686 | LOC100063985 | -1.65 |
| ENSECAT00000013583 | ENSECAG00000012628 | LOC100064464 | -1.33 |
| ENSECAT00000013693 | ENSECAG00000012987 | DIXDC1 | -2.01 |
| ENSECAT00000013714 | ENSECAG00000012847 | LOC100072565 | -1.17 |
| ENSECAT00000013739 | ENSECAG00000012667 | LOC100066233 | -0.96 |
| ENSECAT00000014015 | ENSECAG00000013367 | SLC9A3R2 | -1.43 |
| ENSECAT00000014125 | ENSECAG00000013251 | LOC100051400 | -1.56 |
| ENSECAT00000014483 | ENSECAG00000013613 | | -1.88 |
| ENSECAT00000014511 | ENSECAG00000013613 | | -1.69 |
| ENSECAT00000014760 | ENSECAG00000013291 | LOC100073046 | -1.59 |
| ENSECAT00000014879 | ENSECAG00000014243 | LOC100070511 | -1.82 |
| ENSECAT00000014919 | ENSECAG00000014275 | LOC100146301 | -1.71 |
| ENSECAT00000014948 | ENSECAG00000014210 | C20orf160 | -1.39 |
| ENSECAT00000015010 | ENSECAG00000014231 | TONSL | -1.23 |
| ENSECAT00000015063 | ENSECAG00000014015 | APBB1 | -1.48 |
| ENSECAT00000015173 | ENSECAG00000014515 | LOC100053337 | -1.37 |
| ENSECAT00000015281 | ENSECAG00000012668 | PTPRF | -1.8 |
| ENSECAT00000015365 | ENSECAG00000014219 | SRCIN1 | -1.23 |
| ENSECAT00000015535 | ENSECAG00000014769 | LOC100058602 | -1.19 |
| ENSECAT00000015856 | ENSECAG00000015081 | LOC100054379 | -1.3 |
| ENSECAT00000015887 | ENSECAG00000014474 | LOC100059101 | -1.27 |
| ENSECAT00000015974 | ENSECAG00000015154 | LOC100070930 | -1.38 |
| ENSECAT00000016105 | ENSECAG00000014882 | CORO1B | -1.22 |
| ENSECAT00000016252 | ENSECAG00000015106 | LOC100054391 | -1.51 |
| ENSECAT00000016256 | ENSECAG00000015476 | LOC100050618 | -1.05 |
| ENSECAT00000016320 | ENSECAG00000015612 | LOC100052858 | -1.66 |
| ENSECAT00000016428 | ENSECAG00000015499 | XPC | -1.87 |
| ENSECAT00000016463 | ENSECAG00000015593 | LOC100066378 | -1.46 |
| ENSECAT00000016563 | ENSECAG00000015829 | LOC100068226 | -1.28 |
| ENSECAT00000016629 | ENSECAG00000015789 | LOC100052677 | -1.23 |
| ENSECAT00000016673 | ENSECAG00000015762 | SNAPC4 | -1.39 |
| ENSECAT00000016761 | ENSECAG00000015884 | LOC100061085 | -1.2 |
| ENSECAT00000016779 | ENSECAG00000015950 | CCDC68 | -1.33 |
| ENSECAT00000016798 | ENSECAG00000016028 | LOC100064704 | -0.87 |
| ENSECAT00000016991 | ENSECAG00000016227 | NAGLU | -1.04 |
| ENSECAT00000017222 | ENSECAG00000016210 | ADAMTS10 | -1.49 |
| ENSECAT00000017335 | ENSECAG00000016332 | PIGO | -0.93 |
| ENSECAT00000017426 | ENSECAG00000016217 | RNF213 | -0.94 |
| ENSECAT00000017452 | ENSECAG00000016486 | LOC100070547 | -1.56 |
| ENSECAT00000017673 | ENSECAG00000016820 | KANK2 | -0.98 |
| ENSECAT00000017716 | ENSECAG00000016798 | LOC100050635 | -1.18 |
| ENSECAT00000017842 | ENSECAG00000016625 | LOC100057788 | -1.37 |
| ENSECAT00000017881 | ENSECAG00000016976 | RARB | -0.85 |
| ENSECAT00000018023 | ENSECAG00000017010 | LOC100058549 | -1.17 |
| ENSECAT00000018038 | ENSECAG00000016949 | ZNF594 | -1.99 |
| ENSECAT00000018068 | ENSECAG00000014246 | LOC100069423 | -0.79 |
| ENSECAT00000018085 | ENSECAG00000015514 | LOC100147640 | -9.06 |
| ENSECAT00000018098 | ENSECAG00000016379 | MAPK8IP3 | -2.24 |
| ENSECAT00000018101 | ENSECAG00000017147 | LOC100057278 | -1.52 |
| ENSECAT00000018117 | Known psuedogene | | -1.06 |
| ENSECAT00000018120 | ENSECAG00000016753 | LOC100067531 | -1.16 |
| ENSECAT00000018122 | ENSECAG00000016600 | PPFIA4 | -1.24 |
| ENSECAT00000018133 | ENSECAG00000017017 | LOC100054101 | -1.53 |
| ENSECAT00000018139 | ENSECAG00000015514 | LOC100147640 | -3.55 |
| ENSECAT00000018145 | ENSECAG00000016379 | MAPK8IP3 | -7.57 |
| ENSECAT00000018152 | ENSECAG00000016022 | MADD | -1.59 |
| ENSECAT00000018196 | ENSECAG00000016856 | SLC7A2 | -1.29 |
| ENSECAT00000018199 | ENSECAG00000017227 | LOC100061944 | -1.83 |
| ENSECAT00000018300 | ENSECAG00000017179 | PHC2 | -4 |
| ENSECAT00000018488 | ENSECAG00000017116 | LOC100059685 | -1.3 |
| ENSECAT00000018612 | ENSECAG00000013693 | O46388_HORSE | -4.31 |
| ENSECAT00000018628 | ENSECAG00000017058 | NEK1 | -3.27 |
| ENSECAT00000018835 | ENSECAG00000017826 | LOC100062831 | -1.74 |
| ENSECAT00000019031 | ENSECAG00000018029 | LOC100146573 | -1.71 |
| ENSECAT00000019096 | ENSECAG00000017413 | LOC100067764 | -1.11 |
| ENSECAT00000019139 | ENSECAG00000016864 | LOC100054979 | -1.46 |
| ENSECAT00000019214 | ENSECAG00000017550 | LOC100072696 | -1.24 |
| ENSECAT00000019232 | ENSECAG00000018137 | LOC100055668 | -1.13 |
| ENSECAT00000019252 | ENSECAG00000017484 | LOC100066493 | -3.23 |
| ENSECAT00000019323 | ENSECAG00000013693 | O46388_HORSE | -2.24 |
| ENSECAT00000019335 | ENSECAG00000018173 | LOC100050811 | -1.18 |
| ENSECAT00000019355 | ENSECAG00000017934 | LOC100070527 | -1.42 |
| ENSECAT00000019432 | ENSECAG00000017934 | LOC100070527 | -3.16 |
| ENSECAT00000019592 | ENSECAG00000018310 | LOC100057871 | -1.09 |
| ENSECAT00000019739 | ENSECAG00000017353 | LOC100062842 | -2.02 |
| ENSECAT00000020087 | ENSECAG00000013693 | O46388_HORSE | -1.22 |
| ENSECAT00000020159 | ENSECAG00000018816 | LOC100053170 | -1.44 |
| ENSECAT00000020240 | ENSECAG00000018566 | LOC100057436 | -1.12 |
| ENSECAT00000020291 | ENSECAG00000018928 | PER3 | -1.15 |
| ENSECAT00000020293 | ENSECAG00000018474 | LOC100050861 | -1.04 |
| ENSECAT00000020632 | ENSECAG00000019391 | LOC100064377 | -1.95 |
| ENSECAT00000020693 | ENSECAG00000019067 | LOC100055364 | -1.77 |
| ENSECAT00000020750 | ENSECAG00000019588 | A4UZ24_HORSE | -1.52 |
| ENSECAT00000020765 | ENSECAG00000019551 | LOC100049872 | -1.44 |
| ENSECAT00000020784 | ENSECAG00000019367 | LOC100049851 | -0.89 |
| ENSECAT00000020883 | ENSECAG00000013693 | O46388_HORSE | -7.77 |
| ENSECAT00000020889 | ENSECAG00000019247 | TTC13 | -4.49 |
| ENSECAT00000020941 | ENSECAG00000019779 | LOC100056640 | -1.91 |
| ENSECAT00000021026 | ENSECAG00000019866 | FIGN | -0.92 |
| ENSECAT00000021065 | ENSECAG00000019756 | SLC26A10 | -3.07 |
| ENSECAT00000021193 | ENSECAG00000019756 | SLC26A10 | -1.69 |
| ENSECAT00000021235 | ENSECAG00000018992 | ABCA8 | -2.07 |
| ENSECAT00000021287 | ENSECAG00000019901 | LOC100068731 | -1.15 |
| ENSECAT00000021432 | ENSECAG00000019998 | AKNAD1 | -1.63 |
| ENSECAT00000021557 | ENSECAG00000019918 | ANKS1A | -0.72 |
| ENSECAT00000021577 | ENSECAG00000018147 | AOX1 | -2.56 |
| ENSECAT00000021583 | ENSECAG00000020307 | LOC100065838 | -0.94 |
| ENSECAT00000021647 | ENSECAG00000020213 | PGS1 | -4.34 |
| ENSECAT00000021919 | ENSECAG00000020218 | LOC100065490 | -3.05 |
| ENSECAT00000022026 | ENSECAG00000020314 | ANO1 | -1.75 |
| ENSECAT00000022162 | ENSECAG00000020558 | LOC100062950 | -0.6 |
| ENSECAT00000022396 | ENSECAG00000021092 | LOC100060862 | -1.2 |
| ENSECAT00000022464 | ENSECAG00000021042 | LOC100051887 | -1.11 |
| ENSECAT00000022507 | ENSECAG00000020337 | DOCK11 | -1.37 |
| ENSECAT00000022533 | ENSECAG00000020859 | AGAP3 | -1 |
| ENSECAT00000022586 | ENSECAG00000020691 | LOC100059651 | -1.17 |
| ENSECAT00000022634 | ENSECAG00000020691 | LOC100059651 | -1.68 |
| ENSECAT00000022644 | ENSECAG00000020768 | LOC100059647 | -1.7 |
| ENSECAT00000022950 | ENSECAG00000020835 | LOC100054665 | -3.08 |
| ENSECAT00000023122 | ENSECAG00000021680 | LOC100070896 | -1.36 |
| ENSECAT00000023357 | ENSECAG00000019844 | MYH7_HORSE | -2.62 |
| ENSECAT00000023382 | ENSECAG00000021430 | LOC100066445 | -1.1 |
| ENSECAT00000023503 | ENSECAG00000021756 | LOC100056770 | -1.51 |
| ENSECAT00000023792 | ENSECAG00000020336 | KIF1B | -0.92 |
| ENSECAT00000023795 | ENSECAG00000022332 | LOC100051404 | -0.65 |
| ENSECAT00000023823 | ENSECAG00000021391 | CLASP1 | -1.8 |
| ENSECAT00000023832 | ENSECAG00000022302 | LOC100050927 | -0.98 |
| ENSECAT00000023846 | ENSECAG00000021675 | LOC100056914 | -3.45 |
| ENSECAT00000024175 | ENSECAG00000022121 | LOC100060213 | -1.83 |
| ENSECAT00000024391 | ENSECAG00000022739 | LOC100071384 | -1.36 |
| ENSECAT00000024614 | ENSECAG00000022897 | LOC100147095 | -1.26 |
| ENSECAT00000024899 | ENSECAG00000023163 | LOC100067795 | -1.34 |
| ENSECAT00000024900 | ENSECAG00000023268 | LOC100066978 | -1.72 |
| ENSECAT00000024971 | ENSECAG00000023217 | LOC100146295 | -1.11 |
| ENSECAT00000025098 | ENSECAG00000022556 | LOC100062524 | -3.3 |
| ENSECAT00000025145 | ENSECAG00000022873 | ATP8A1 | -1.55 |
| ENSECAT00000025300 | ENSECAG00000023111 | LOC100057452 | -2.27 |
| ENSECAT00000025353 | ENSECAG00000023676 | LOC100049936 | -0.86 |
| ENSECAT00000025367 | ENSECAG00000023588 | EHHADH | -1.01 |
| ENSECAT00000025427 | ENSECAG00000023334 | LOC100071840 | -1.84 |
| ENSECAT00000025591 | ENSECAG00000023798 | LOC100054889 | -2.05 |
| ENSECAT00000025647 | ENSECAG00000023920 | LOC100060676 | -1.1 |
| ENSECAT00000025685 | ENSECAG00000023946 | LOC100070525 | -1.25 |
| ENSECAT00000025704 | ENSECAG00000023907 | WDR6 | -1.07 |
| ENSECAT00000025753 | ENSECAG00000023793 | LOC100059087 | -1.09 |
| ENSECAT00000025910 | ENSECAG00000023905 | LOC100067247 | -1.12 |
| ENSECAT00000025997 | ENSECAG00000024202 | LOC100070799 | -1.46 |
| ENSECAT00000026104 | ENSECAG00000024297 | LOC100051633 | -3.89 |
| ENSECAT00000026341 | ENSECAG00000024113 | ABLIM1 | -0.79 |
| ENSECAT00000026377 | ENSECAG00000024517 | | -1.3 |
| ENSECAT00000026424 | ENSECAG00000024549 | LOC100061258 | -1.58 |
| ENSECAT00000026441 | ENSECAG00000024423 | Q30BK7_HORSE | -1.29 |
| ENSECAT00000026478 | ENSECAG00000024480 | TBX2 | -1.74 |
| ENSECAT00000026490 | ENSECAG00000024500 | LOC100147266 | -1.21 |
| ENSECAT00000026546 | ENSECAG00000024533 | LOC100069575 | -1.13 |
| ENSECAT00000026766 | ENSECAG00000024769 | O97950_HORSE | -1.24 |
| ENSECAT00000026767 | ENSECAG00000024745 | USH1C | -1.29 |
| ENSECAT00000026798 | ENSECAG00000024839 | MBD5 | -4.55 |
| ENSECAT00000026829 | Known psuedogene | | -1.23 |
| ENSECAT00000026834 | ENSECAG00000024886 | LOC100071548 | -1.09 |
| ENSECAT00000026907 | ENSECAG00000024939 | ZNF385A | -2.08 |
| ENSECAT00000026953 | ENSECAG00000024966 | RAVER1 | -1.57 |
| ENSECAT00000026956 | ENSECAG00000024973 | LOC100051718 | -1.97 |
| ENSECAT00000027124 | ENSECAG00000025115 | CC2D2A | -2.99 |
| ENSECAT00000028992 | ENSECAG00000020433 | LOC100051352 | -0.89 |
| ENSECAT00000029018 | ENSECAG00000026913 | | -0.78 |

1. List of up-regulated DEGs in blood tissue

| **Transcript name** | **Gene name (Ensembl)** | **Gene name** | **Average log2 ratio** |
| --- | --- | --- | --- |
| Cluster 12565 | Novel gene |  | 1.3 |
| Cluster 18603 | Novel gene |  | 1.62 |
| Cluster 19804 | Novel gene |  | 2.23 |
| Cluster 20343 | Novel gene |  | 1.4 |
| Cluster 23120 | Novel gene |  | 1.34 |
| Cluster 24137 | Novel gene |  | 3.46 |
| Cluster 25386 | Novel gene |  | 1.2 |
| Cluster 28671 | Novel gene |  | 1.52 |
| Cluster 30602 | Novel gene |  | 1.27 |
| Cluster 33676 | Novel gene |  | 1.47 |
| Cluster 34818 | Novel gene |  | 0.32 |
| Cluster 34849 | Novel gene |  | 1.13 |
| Cluster 35502 | Novel gene |  | 1.76 |
| Cluster 35526 | Novel gene |  | 1.29 |
| Cluster 41786 | Novel gene |  | 1.01 |
| Cluster 518 | Novel gene |  | 1.97 |
| Cluster 5910 | Novel gene |  | 1.27 |
| Cluster 7691 | Novel gene |  | 0.88 |
| ENSECAT00000000047 | ENSECAG00000000015 | LOC100055669 | 0.48 |
| ENSECAT00000000176 | ENSECAG00000000207 | ACTA1 | 0.72 |
| ENSECAT00000001012 | ENSECAG00000001110 | LOC100050888 | 1.34 |
| ENSECAT00000003005 | ENSECAG00000000836 | Q95MB0_HORSE | 3.28 |
| ENSECAT00000003790 | ENSECAG00000003823 | LOC100050888 | 1.4 |
| ENSECAT00000006178 | ENSECAG00000006067 | USP48 | 1.79 |
| ENSECAT00000008016 | ENSECAG00000005833 | PTPRK | 2.18 |
| ENSECAT00000008343 | ENSECAG00000002429 | MED12L | 33.28 |
| ENSECAT00000009943 | ENSECAG00000009275 | AARSD1 | 2.44 |
| ENSECAT00000010174 | ENSECAG00000009958 | LOC100069834 | 1.3 |
| ENSECAT00000010863 | ENSECAG00000009336 | LOC100061058 | 1.82 |
| ENSECAT00000010991 | ENSECAG00000010560 | B7UBX3_HORSE | 1.52 |
| ENSECAT00000011049 | ENSECAG00000010324 | | 1.56 |
| ENSECAT00000011109 | ENSECAG00000010078 | CIT | 5.41 |
| ENSECAT00000012015 | ENSECAG00000011276 | FAM160B1 | 0.95 |
| ENSECAT00000013362 | ENSECAG00000012635 | LOC100058937 | 1.53 |
| ENSECAT00000014432 | ENSECAG00000013231 | CLPTM1L | 2.93 |
| ENSECAT00000014446 | ENSECAG00000013245 | LOC100050841 | 1.56 |
| ENSECAT00000014856 | ENSECAG00000014260 | LOC100051632 | 2.42 |
| ENSECAT00000018145 | ENSECAG00000016379 | MAPK8IP3 | 9.01 |
| ENSECAT00000018852 | ENSECAG00000017346 | LOC100056731 | 0.97 |
| ENSECAT00000019012 | ENSECAG00000017901 | LOC100062490 | 1.17 |
| ENSECAT00000019056 | ENSECAG00000018002 | LOC100065680 | 2.85 |
| ENSECAT00000019339 | ENSECAG00000017904 | DUS1L | 2.71 |
| ENSECAT00000020163 | ENSECAG00000018428 | LOC100062150 | 1.81 |
| ENSECAT00000021205 | ENSECAG00000019708 | LOC100050532 | 1.63 |
| ENSECAT00000022162 | ENSECAG00000020558 | LOC100062950 | 1.42 |
| ENSECAT00000022178 | ENSECAG00000020664 | LOC100072289 | 0.54 |
| ENSECAT00000023515 | ENSECAG00000020933 | ITPR1 | 0.73 |
| ENSECAT00000023788 | ENSECAG00000020336 | KIF1B | 1.22 |
| ENSECAT00000024120 | ENSECAG00000022472 | LOC100146297 | 1.05 |
| ENSECAT00000024193 | ENSECAG00000022117 | LOC100064689 | 1.45 |
| ENSECAT00000024490 | ENSECAG00000022890 | LOC100054178 | 0.55 |
| ENSECAT00000024991 | ENSECAG00000022405 | NOTCH1 | 1.45 |
| ENSECAT00000025135 | ENSECAG00000022653 | B0I1H0_HORSE | 0.72 |
| ENSECAT00000025472 | ENSECAG00000023539 | VPS8 | 1.03 |
| ENSECAT00000025604 | ENSECAG00000023253 | LOC100054721 | 0.57 |
| ENSECAT00000025749 | ENSECAG00000023793 | LOC100059087 | 5.6 |
| ENSECAT00000025794 | ENSECAG00000023978 | | 0.99 |
| ENSECAT00000026266 | ENSECAG00000024246 | GIGYF2 | 8.66 |
| ENSECAT00000026382 | ENSECAG00000024334 | LRRC16A | 2.19 |
| ENSECAT00000026515 | ENSECAG00000024461 | SF3B2 | 1.55 |
| ENSECAT00000027073 | ENSECAG00000025059 | LOC100053057 | 2.28 |
| ENSECAT00000027105 | ENSECAG00000025107 | LOC100061847 | 1.84 |

1. List of down-regulated DEGs in blood tissue

| **Transcript name** | **Gene name (Ensembl)** | **Gene name** | **Average log2 ratio** |
| --- | --- | --- | --- |
| Cluster 1222 | Novel gene |  | -2.14 |
| Cluster 1247 | Novel gene |  | -2.25 |
| Cluster 1259 | Novel gene |  | -2.39 |
| Cluster 18835 | Novel gene |  | -1.28 |
| Cluster 19162 | Novel gene |  | -1.11 |
| Cluster 20362 | Novel gene |  | -2.2 |
| Cluster 21102 | Novel gene |  | -1.25 |
| Cluster 25906 | Novel gene |  | -0.8 |
| Cluster 25907 | Novel gene |  | -1.86 |
| Cluster 2869 | Novel gene |  | -2.05 |
| Cluster 28830 | Novel gene |  | -1.43 |
| Cluster 29457 | Novel gene |  | -1.63 |
| Cluster 31183 | Novel gene |  | -0.83 |
| Cluster 3256 | Novel gene |  | -3.06 |
| Cluster 34327 | Novel gene |  | -0.76 |
| Cluster 3665 | Novel gene |  | -1.27 |
| Cluster 36737 | Novel gene |  | -4.06 |
| Cluster 38412 | Novel gene |  | -1.36 |
| Cluster 3879 | Novel gene |  | -0.98 |
| Cluster 39637 | Novel gene |  | -1.53 |
| Cluster 39719 | Novel gene |  | -1.36 |
| Cluster 41573 | Novel gene |  | -2.21 |
| Cluster 478 | Novel gene |  | -1.74 |
| Cluster 6966 | Novel gene |  | -1.53 |
| ENSECAT00000002826 | ENSECAG00000002984 | LOC100065485 | -1.47 |
| ENSECAT00000004370 | ENSECAG00000002173 | LOC100053589 | -2.41 |
| ENSECAT00000004839 | ENSECAG00000004913 | LPAR6 | -1.42 |
| ENSECAT00000005088 | ENSECAG00000000631 | LOC100050973 | -1.67 |
| ENSECAT00000005829 | ENSECAG00000000767 | SLC4A7 | -1.03 |
| ENSECAT00000006655 | ENSECAG00000006543 | LOC100052387 | -0.73 |
| ENSECAT00000007012 | ENSECAG00000006851 | | -1.23 |
| ENSECAT00000007393 | ENSECAG00000006377 | SEC16A | -1.4 |
| ENSECAT00000008021 | ENSECAG00000005833 | PTPRK | -1.39 |
| ENSECAT00000008320 | ENSECAG00000002429 | MED12L | -24.54 |
| ENSECAT00000009029 | ENSECAG00000008307 | UBE4B | -15.38 |
| ENSECAT00000011821 | ENSECAG00000010790 | COG6 | -1.81 |
| ENSECAT00000012096 | ENSECAG00000011111 | LOC100050357 | -1.33 |
| ENSECAT00000012302 | ENSECAG00000011111 | LOC100050357 | -3.47 |
| ENSECAT00000012575 | ENSECAG00000011582 | DLGAP4 | -12.54 |
| ENSECAT00000012935 | ENSECAG00000011234 | LOC100063696 | -1.67 |
| ENSECAT00000013510 | ENSECAG00000011972 | UBR1 | -1.9 |
| ENSECAT00000014203 | ENSECAG00000013660 | LOC100055210 | -1.18 |
| ENSECAT00000015036 | ENSECAG00000014218 | LOC100066351 | -1.6 |
| ENSECAT00000015473 | ENSECAG00000013676 | CAST | -1.41 |
| ENSECAT00000015533 | ENSECAG00000014347 | Q4KU44_HORSE | -1.5 |
| ENSECAT00000015628 | ENSECAG00000014250 | | -1.49 |
| ENSECAT00000016023 | ENSECAG00000014922 | LOC100067366 | -0.91 |
| ENSECAT00000016091 | ENSECAG00000013677 | FRY | -0.64 |
| ENSECAT00000016130 | ENSECAG00000015063 | LOC100060706 | -1.93 |
| ENSECAT00000016623 | ENSECAG00000015413 | LOC100063073 | -1.74 |
| ENSECAT00000016784 | ENSECAG00000015440 | TBC1D9 | -0.79 |
| ENSECAT00000017423 | ENSECAG00000016473 | ANKZF1 | -1.25 |
| ENSECAT00000017524 | ENSECAG00000015910 | KTN1 | -2.48 |
| ENSECAT00000017771 | ENSECAG00000016768 | GATA2 | -1.61 |
| ENSECAT00000017918 | ENSECAG00000016150 | VPS13D | -2.06 |
| ENSECAT00000018480 | ENSECAG00000017220 | MX2 | -0.94 |
| ENSECAT00000018503 | ENSECAG00000017559 | LOC100061845 | -1 |
| ENSECAT00000018697 | ENSECAG00000017520 | LOC100055892 | -1.15 |
| ENSECAT00000018724 | ENSECAG00000016582 | | -1.41 |
| ENSECAT00000019195 | ENSECAG00000016864 | LOC100054979 | -1.82 |
| ENSECAT00000019577 | ENSECAG00000018012 | Q6DTK4_HORSE | -2.04 |
| ENSECAT00000019802 | ENSECAG00000017799 | MYO1G | -1.6 |
| ENSECAT00000022846 | ENSECAG00000020168 | Q8WN21_HORSE | -1.11 |
| ENSECAT00000023351 | ENSECAG00000021934 | CC2D1B | -1.84 |
| ENSECAT00000023743 | ENSECAG00000020336 | KIF1B | -8.34 |
| ENSECAT00000024151 | ENSECAG00000022117 | LOC100064689 | -1.54 |
| ENSECAT00000024160 | ENSECAG00000022503 | LOC100060212 | -1.05 |
| ENSECAT00000024385 | ENSECAG00000022603 | PPP4R1L | -2.89 |
| ENSECAT00000024857 | ENSECAG00000023122 | PALLD | -0.69 |
| ENSECAT00000025134 | ENSECAG00000022950 | LOC100059072 | -4.48 |
| ENSECAT00000025590 | ENSECAG00000023253 | LOC100054721 | -1.15 |
| ENSECAT00000025685 | ENSECAG00000023946 | LOC100070525 | -1 |
| ENSECAT00000026276 | ENSECAG00000024128 | LOC100052467 | -1.46 |
| ENSECAT00000026331 | ENSECAG00000024113 | ABLIM1 | -2.68 |
| ENSECAT00000026671 | ENSECAG00000024638 | MED23 | -1.5 |
| ENSECAT00000027104 | ENSECAG00000025107 | LOC100061847 | -1.37 |
| ENSECAT00000027178 | ENSECAG00000025168 | LOC100073102 | -0.71 |
| ENSECAT00000028924 | ENSECAG00000010552 | | -3.01 |
| ENSECAT00000029034 | ENSECAG00000025132 | LOC100072600 | -2.55 |
| ENSECAT00000029077 | ENSECAG00000014364 | LOC100062679 | -2.69 |

**Supplementary Table S17**. Expression profiles of known exercise-related horse genes.

| **Exercise-related genes** | **Ensembl transcript name** | **Average log2 ratio** | |
| --- | --- | --- | --- |
| Alpha 3 actinin | ENSECAT00000021149 | -0.10 | -0.14 |
| Myosin light chain | ENSECAT00000016385 | 0.10 | -0.27 |
| Insulin-like growth factor I | ENSECAT00000010554 | N/D | N/D |
| Myostatin | ENSECAT00000022792 | N/D | N/D |
| B2 bradykinin receptor | ENSECAT00000005325 | N/D | N/D |
| Hypoxia-inducible factor 1, alpha subunit (HIF1A) | ENSECAT00000016943 | 1.85 | -0.14 |
| Beta-2 adrenergic receptor (ADRB2) | ENSECAT00000004802 | 1.72 | -0.25 |
| Muscle creatine kinase | ENSECAT00000023921 | -0.11 | 0.15 |
| Glycogen synthase 1 (muscle) | ENSECAT00000023453 | -0.49 | -0.08 |
| Vitamin D3 receptor | ENSECAT00000016853 | N/D | -0.19 |
| Peroxisome proliferator-activated receptor delta (PPARD) | ENSECAT00000008669 | 2.69 | 0.00 |
| peroxisome proliferator activated receptor alpha (PPARA) | ENSECAT00000000699 | -0.59 | -0.37 |
| Vascular endothelial growth factor (VEGF) | ENSECAT00000009850 | 1.54 | 0.02 |
| Endothelial PAS domain-containing protein 1 (EPAS-1) | ENSECAT00000015749 | 0.52 | 0.74 |
| Angiotensin I converting enzyme 2 | ENSECAT00000002458 | N/D | N/D |
| CAD36004.1| unnamed protein product | ENSECAT00000008036 | N/D | N/D |
| Procollagen alpha 1 (I) | ENSECAT00000020087 | N/D | N/D |
| Tenascin c | ENSECAT00000019034 | 3.25 | -0.70 |
| Matrix metalloproteinase 3 | ENSECAT00000002326 | N/D | N/D |
| Dopamine D1 receptor | ENSECAT00000011983 | N/D | N/D |
| Dopamine D2 receptor | ENSECAT00000014466 | N/D | N/D |
| Dopamine receptor D3 | ENSECAT00000017512 | N/D | N/D |
| D4 dopamine receptor | ENSECAT00000010377 | N/D | N/D |
| Serotonin transporter | ENSECAT00000018693 | N/D | N/D |
| Brain-derived neurotrophic factor (BDNF) | ENSECAT00000016720 | 2.59 | N/D |

**Supplementary Table S18. Comparison between DEGs in muscle tissue and the DEGs which are responsible to exercise training .**

1. **Up-regulated DEGs which are responsible to exercise training**

| **Gene symbol** | **Tag** | **Gene name** | **FC1^a^** | **Ensembl annotation** | **FC2^a^** |
| --- | --- | --- | --- | --- | --- |
| ACADVL | gctgctctgcagtctga | Acyl-Coenzyme A dehydrogenase, very long chain | 0.72 | ENSECAT00000026882 | No DEG |
| ACTR3B | gaataattgaagactgg | Arp3 Actin-Related Protein 3 Homolog B | 2.37 | ENSECAT00000019685 | 1.25 |
| C14orf153 | gcgtccttgaggtccgg | Chromosome 14 open reading frame 153 | 1.14 | ENSECAT00000013952 | No DEG |
| CUL3 | ctgtttttctgtttttt | Cullin 3 | 1.43 | ENSECAT00000012128 | No DEG |
| FBXO32 | tgataccaatattcagt | F-box protein 32 | 1.17 | ENSECAT00000017032 | 1.69 |
| GOT1 | cagaaagagcagggaag | Glutamic-oxaloacetic transaminase 1, soluble | 1.46 | ENSECAT00000000188 | No DEG |
| GRHPR | tggatgtgtggctatgg | Glyoxylate Reductase/Hydroxypyruvate Reductase | 1.45 | ENSECAT00000009721 | No DEG |
| IGFN1 | ggacccatgaaggacca | Immunoglobulin-like and fibronectin type III domain-containing protein 1 | 2.03 | ENSECAT00000000246 | No DEG |
| KIAA1303 | tggttctgtttgttttg | P150 target of rapamycin (TOR)-scaffold protein | 2.7 | ENSECAT00000019239 | No DEG |
| MRPS21 | gagtgcagcctttcacc | 28 S ribosomal protein S21, mitochondrial | 3.24 | ENSECAT00000010095 | No DEG |
|  | accagagagatgaatgt |  | 1.76 |  |  |
| PER2 | tgttgaagcgatgcagt | Period homolog 2 | 29.26 | ENSECAT00000014506 | No DEG |
| PER3 | tgttggtaagtagatcg | Period homolog 3 (Drosophila) | 1.05 | ENSECAT00000020291 | -1.15 |
| SLC25A29 | tggctgtatggggaggc | Solute carrier family 25 member 29 | 1.32 | ENSECAT00000006414 | No DEG |
| TNNT3 | gccttctgcacccagaa | Troponin T Type 3 (Skeletal, Fast) | 1.55 | ENSECAT00000025982 | No DEG |
| ZAK | ttaaatatacttggaag | Sterile alpha motif and leucine zipper containing kinase AZK | 2.08 | ENSECAT00000000576 | No DEG |

^a^Fold change in the previous study

^b^Fold change in this study

1. **Down-regulated DEGs which are responsible to exercise training**

| **Gene symbol** | **Tag** | **Gene name** | **FC1^a^** | **Ensembl annotation** | **FC2^a^** |
| --- | --- | --- | --- | --- | --- |
| ACTN3 | acccgagagacagccga | actinin, alpha 3 | -0.97 | ENSECAT00000021149 | No DEG |
| AHCYL2 | acacagttagttaattt | Putative adenosylhomocysteinase 3 | -1.39 | ENSECAT00000015484 | No DEG |
|  | acacagttagttaattt |  | -1.39 |  |  |
| ANKHD1 | taattttatttttttta | Ankyrin repeat and KH domain containing 1 | -2.3 | ENSECAT00000016611 | No DEG |
| APOOL | ttttccctcacatcttc | Apolipoprotein O-like | -0.54 | ENSECAT00000009540 | No DEG |
| ATP2B1 | cttagtgtgtatatctc | ATPase, Ca++ transporting, plasma membrane 1 | -0.62 | ENSECAT00000009733 | No DEG |
| BCL6 | atcattattttaccttt | B-cell CLL/lymphoma 6 | -3.09 | ENSECAT00000005687 | No DEG |
| C1orf51 | acggttttccccagatc | Chromosome 1 open reading frame 51 | -1.61 | ENSECAT00000010683 | 1.61 |
| C21orf7 | cactggccaaaagattt | Chromosome 21 open reading frame 7 | -3.08 | ENSECAT00000022903 | No DEG |
| CALM3 | ccactaccctcttactc | Calmodulin3 | -1.46 | ENSECAT00000009414 | No DEG |
|  | acagacacttggctaaa |  | -0.83 |  |  |
| CCNDBP1 | aacagaatcaaggagct | Cyclin-D1-binding protein 1 | -0.81 | ENSECAT00000012136 | No DEG |
| DAG1 | gaaaacagtagctaaag | dystroglycan 1 | -1.49 | ENSECAT00000009899 | No DEG |
| EIF3F | ctcaacagcaacatcaa | Eukaryotic translation initiation factor 3, subunit F | -0.52 | ENSECAT00000001158 | No DEG |
| FBXL17 | tccagcctcaaagcatt | F-Box And Leucine-Rich Repeat Protein 17 | -0.52 | ENSECAT00000012274 | No DEG |
| GATM | aactgtagtgctttaaa | Glycine Amidinotransferase | -1.35 | ENSECAT00000014879 | -1.82 |
|  | taggttttacctccatt |  | -1.35 |  |  |
| GLUL | ctggaacaggggcgaac | Glutamate-ammonia ligase | -3.94 | ENSECAT00000016824 | No DEG |
| GPSN2 | cccatcatccccttcct | GLYCOPROTEIN, SYNAPTIC 2 | -1.06 | ENSECAT00000020391 | No DEG |
| GSTM5 | aagtcccaccccaatat | Glutathione S-transferase M5 | -1.1 | ENSECAT00000026303 | No DEG |
| HOXC9 | tccacccataagcagat | Homeobox protein Hox-C9 | -1.04 | ENSECAT00000000599 | No DEG |
| IGFBP5 | ggactgtctttattttt | Insulin-like growth factor binding protein-5 | -2.32 | ENSECAT00000014055 | No DEG |
|  | gtaaccctacacagtca |  | -2.12 |  |  |
| IRF2BP2 | cccagaaagacatttgt | Interferon regulatory factor 2 binding protein 2 | -1.57 | ENSECAT00000025106 | No DEG |
| KCMF1 | caaaaggctctcctaat | potassium channel modulatory factor 1 | -0.81 | ENSECAT00000026787 | No DEG |
| KPNA1 | tttccattcaacaaaaa | Karyopherin alpha 1 | -1.8 | ENSECAT00000018698 | No DEG |
| KPNA3 | aattactctttcactgt | Karyopherin alpha 3 | -1.55 | ENSECAT00000017211 | No DEG |
| LRRFIP1 | cttttcacacacaaaac | Leucine Rich Repeat | -0.95 | ENSECAT00000017623 | No DEG |
|  |  |  |  | ENSECAT00000017654 | No DEG |
|  |  |  |  | ENSECAT00000017689 | No DEG |
| MAP3K4 | ttaagtgccattactac | Mitogen-activated protein kinase kinase kinase 4 | -0.85 | ENSECAT00000013945 | No DEG |
| MLEC | ccccaccctactcccac | Malectin | -1.13 | ENSECAT00000023785 | No DEG |
| MSTN | tatgacagaaaagcaac | Myostatin | -2.56 |  | No DEG |
|  | atgactgtataatgtga |  | -2.55 |  |  |
|  | gttcctaaataaataat |  | -4.2 | ENSECAT00000022792 |  |
| MYLK2 | ctgctgagcggcctctc | Myosin Light Chain Kinase 2, Skeletal Muscle | -1.94 | ENSECAT00000011562 | No DEG |
| MYO9A | gctcattaaagaacaaa | Myosin Ixa | -1.03 | ENSECAT00000017899 | No DEG |
|  |  |  |  | ENSECAT00000017832 | No DEG |
| NAT12 | ctatcttttccttttct | N-acetyltransferase MAK3 homolog | -0.68 | ENSECAT00000013399 | No DEG |
| NEDD4 | attgtttaaatatcact | Neural precursor cell expressed, developmentally down-regulated 4 | -0.97 | ENSECAT00000013007 | No DEG |
| NLN | aaatcccaccctcccct | Neurolysin | -1.02 | ENSECAT00000012307 | No DEG |
| PALLD | tccagctttctattctt | Palladin, cytoskeletal associated protein | -1.06 | ENSECAT00000024847 | No DEG |
|  |  |  |  | ENSECAT00000024857 | No DEG |
| PCBP4 | cttctttccccacctcc | Poly(rC) binding protein 4 | -0.82 | ENSECAT00000026188 | No DEG |
| GNPNAT1 | taattgcagtttactat | Similar to glucosamine-phosphate N-acetyltransferase 1 | -1.23 | ENSECAT00000020943 | No DEG |
| PTDSS1 | ctctagaacatttacct | Phosphatidylserine synthase 1 | -0.62 | ENSECAT00000017216 | No DEG |
| PTPLA | agagtcaatataaaggt | Protein Tyrosine Phosphatase-Like | -2.07 | ENSECAT00000021501 | No DEG |
| RAB33B | aaatctaaagttaaata | RAB33B, member RAS oncogene family | -1.24 | ENSECAT00000020853 | No DEG |
| RYK | cagccctgggggcctac | Ryk Receptor-Like Tyrosine Kinase | -0.94 | ENSECAT00000008925 | No DEG |
| SEPT7 | attcccatttctagtaa | Transcribed locus | -0.72 | ENSECAT00000024587 | No DEG |
| SLC10A2 | tgaggacaaagctcagg | Solute carrier family 10 | -4.11 | ENSECAT00000006307 | No DEG |
| SNAP29 | ccttcaactcaacaaat | Synaptosomal-associated protein, 29 kDa | -0.43 | ENSECAT00000021742 | No DEG |
| SNORA24 | gtattgtaagatattaa | Small nucleolar RNA SNORA24 | -0.89 | ENSECAT00000028045 | No DEG |
|  |  |  |  | ENSECAT00000027643 | No DEG |
| TET1 | aagcaagaaataaattt | Tet oncogene 1 | -1.4 | ENSECAT00000016139 | No DEG |
| TLE1 | atgcagagcaccacaga | transducin-like enhancer of split 1 | -1.58 | ENSECAT00000015131 | No DEG |
| TOR3A | actgacatatgtaaaga | torsin family 3, member A | -1.49 | ENSECAT00000019110 | No DEG |
| TPD52 | tgcctatcacctgccgg | Tumor protein D52 | -0.68 | ENSECAT00000014729 | No DEG |
| TSPAN3 | tcctttcagtcttcaca | Tetraspanin 3 | -0.58 | ENSECAT00000014849 | No DEG |
| ULK2 | ctttgtgacttccaagt | Unc-51-like kinase 2 | -0.72 | ENSECAT00000009563 | No DEG |
| VCL | aaaccatatttcttccc | Vinculin | -1.13 | ENSECAT00000014550 | No DEG |
|  |  |  |  | ENSECAT00000014556 | No DEG |
|  |  |  |  | ENSECAT00000014559 | No DEG |
| ZC3H3 | cccaggcccgtccctgc | Zinc finger CCCH-type containing 3 | -1.28 | ENSECAT00000013097 | No DEG |
| ZNF704 | aagtctaacttccattt | Zinc finger protein 704 | -0.93 | ENSECAT00000013396 | No DEG |
| ZWINT | ttagtttcttttcttta | ZW10 interactor | -1.21 | ENSECAT00000000583 | No DEG |

^a^Fold change in the previous study

^b^Fold change in this study

**Supplementary Table S19. List of transcription factors differentially expressed in muscle and blood tissues.**

1. Muscle tissue

| **No** | **Transcript name** | **Gene name** | **Average Log2 ratio** | **Description of general functions** |
| --- | --- | --- | --- | --- |
| 1 | ENSECAT00000011931 | LOC100050849 | 5.62 | - |
| 2 | ENSECAT00000014939 | NR4A2 | 5.29 | In smooth muscle cells, Nurr1 inhibits proliferation and inflammatory responses. It is a potential transcriptional mediator of inflammatory signals in activated macrophages |
| 3 | ENSECAT00000014856 | LOC100051632 | 4.91 | - |
| 4 | ENSECAT00000004699 | RUNX1 | 4.9 | Core binding factor (CBF) is a heterodimeric transcription factor that binds to the core element of many enhancers and promoters. The target genes are thought to be involved in the development of normal hematopoiesis. |
| 5 | ENSECAT00000022500 | LOC100052048 | 3.62 | - |
| 6 | ENSECAT00000025456 | EGR3 | 3.46 | Egr transcription factors are essential for conversion of the mitogenic signal of the epidermal growth factor into a proliferative response. |
| 7 | ENSECAT00000011460 | LOC100051444 | 3.29 | - |
| 8 | ENSECAT00000029053 | CREB5 | 3.1 | The product of this gene belongs to the CRE- (cAMP response element) binding protein family. Members of this family contain zinc-finger and bZIP DNA-binding domains. The encoded protein specifically binds to CRE as a homodimer or a heterodimer with c-Jun or CRE-BP1, and functions as a CRE-dependent trans-activator. |
| 9 | ENSECAT00000021435 | CREB5 | 1.99 |
| 10 | ENSECAT00000013715 | LOC100062273 | 3.01 | - |
| 11 | ENSECAT00000022937 | Q9N1W3_HORSE | 2.96 | - |
| 12 | ENSECAT00000012354 | ZNF217 | 2.91 | ZNF217 assembles a distinct set of histone modifying proteins at target DNA sites that act synergistically in transcriptional repression. |
| 13 | ENSECAT00000029140 | ZNF217 | 1.12 |
| 14 | ENSECAT00000008669 | LOC100053404 | 2.8 | - |
| 15 | ENSECAT00000025803 | LOC100067469 | 2.74 | - |
| 16 | ENSECAT00000014165 | LOC100065093 | 2.68 | - |
| 17 | ENSECAT00000013536 | ZNF295 | 2.57 | ZNF295 encodes the zinc-finger transcription factor; however, its function has not been characterized yet. |
| 18 | ENSECAT00000010895 | LOC100052058 | 2.53 | - |
| 19 | ENSECAT00000024561 | LOC100069444 | 2.5 | - |
| 20 | ENSECAT00000010038 | LOC100071451 | 2.5 | - |
| 21 | ENSECAT00000018999 | LOC100063253 | 2.47 | - |
| 22 | ENSECAT00000022322 | LOC100070664 | 2.36 | - |
| 23 | ENSECAT00000003723 | LOC100065217 | 2.35 | - |
| 24 | ENSECAT00000019701 | LOC100067790 | 2.31 | - |
| 25 | ENSECAT00000008241 | BAZ1A | 2.29 | BAZ1A is an accessory subunit of the ATP-dependent chromatin assembly factor (ACF), a member of the ISWI (‘imitation switch’) family of chromatin remodeling complexes. |
| 26 | ENSECAT00000015683 | LOC100068622 | 2.22 | - |
| 27 | ENSECAT00000013742 | LOC100067188 | 2.21 | - |
| 28 | ENSECAT00000019925 | LOC100060953 | 2.15 | - |
| 29 | ENSECAT00000017731 | LOC100061353 | 2.15 | - |
| 30 | ENSECAT00000010015 | LOC100073197 | 2.15 | - |
| 31 | ENSECAT00000015549 | XBP1 | 2.1 | XBP1 is a key regulator of mammalian ER stress response. |
| 32 | ENSECAT00000009314 | PRDM1 | 2.09 | PRDM1 acts as a repressor of beta-interferon gene expression. It especially binds to beta-IFN gene promoters. A key role for PRDM1 in the negative regulation of NK activation. |
| 33 | ENSECAT00000022451 | CAMTA1 | 2 | CAMTA1’s function has not been characterized yet. |
| 34 | ENSECAT00000025307 | LOC100054274 | 1.96 | - |
| 35 | ENSECAT00000026339 | LOC100051397 | 1.93 | - |
| 36 | ENSECAT00000021905 | Q865B2_HORSE | 1.92 | - |
| 37 | ENSECAT00000016943 | LOC100061166 | 1.9 | - |
| 38 | ENSECAT00000020973 | LOC100064714 | 1.88 | - |
| 39 | ENSECAT00000007199 | LOC100062536 | 1.86 | - |
| 40 | ENSECAT00000010837 | ZNF398 | 1.86 | This gene encodes a member of the Kruppel family of C2H2-type zinc-finger transcription factor proteins. |
| 41 | ENSECAT00000021368 | LOC100069984 | 1.85 | - |
| 42 | ENSECAT00000022828 | LOC100070969 | 1.82 | - |
| 43 | ENSECAT00000015869 | LOC100059809 | 1.75 | - |
| 44 | ENSECAT00000025292 | ETV6 | 1.73 | Expression of the ETV6-NTRK3 gene fusion is a primary event in human secretory breast carcinoma. |
| 45 | ENSECAT00000011058 | KLF4 | 1.72 | KLF4 is necessary for preventing the entry into mitosis following DNA damage. |
| 46 | ENSECAT00000012055 | - | 1.62 | - |
| 47 | ENSECAT00000015842 | LOC100060644 | 1.6 | - |
| 48 | ENSECAT00000018255 | Q6X9Y0_HORSE | 1.6 | - |
| 49 | ENSECAT00000021922 | GLIS2 | 1.56 | GLIS2 promotes neuronal differentiation and regulates kidney morphogenesis. |
| 50 | ENSECAT00000009359 | LOC100050053 | 1.56 | - |
| 51 | ENSECAT00000005851 | NFE2L2 | 1.52 | This protein has basic leucine zipper (bZIP) transcription factors which are different from normal bZIP TFs such as JUN and FOS. It regulates EpRE/Nrf2 signaling pathways by c-Myc through both interaction with the EpRE binding complex and increased degradation of Nrf2. |
| 52 | ENSECAT00000015344 | KDM5B | 1.5 | KDM5B regulates chromatin structure and transcription through a KDM5B-dependent pathway. |
| 53 | ENSECAT00000010456 | ZSCAN10 | 1.48 | Zfp206 is an embryonic transcription factor that plays a role in regulating pluripotency of embryonic stem cells. |
| 54 | ENSECAT00000025983 | - | 1.47 | - |
| 55 | ENSECAT00000016309 | LOC100067894 | 1.47 | - |
| 56 | ENSECAT00000019058 | LOC100056246 | 1.44 | - |
| 57 | ENSECAT00000011498 | O62767_HORSE | 1.44 | - |
| 58 | ENSECAT00000010956 | LOC100057650 | 1.4 | - |
| 59 | ENSECAT00000010020 | IRF7 | 1.37 | IRF7 has a role in the transcriptional activation of virus-inducible cellular genes, including type I interferon genes. |
| 60 | ENSECAT00000025016 | LOC100056907 | 1.37 | - |
| 61 | ENSECAT00000009562 | LOC100057639 | 1.36 | - |
| 62 | ENSECAT00000020453 | ZNF800 | 1.36 | ZNF800 encodes the zinc-finger transcription factor; however, its function has not been characterized yet. |
| 63 | ENSECAT00000023598 | ELF2 | 1.29 | ELF2/NERF promotes VCP transcription; ELF2/NERF-VCP pathways may be important for cell survival and proliferation under cytokine stress. |
| 64 | ENSECAT00000019521 | ETF1 | 1.26 | ETF1 encodes eukaryotic translation termination factor 1. |
| 65 | ENSECAT00000013604 | ZBTB10 | 1.22 | ZBTB10 encodes a protein containing zinc finger and BTB domains together. |
| 66 | ENSECAT00000011300 | ERG | 1.18 | TMPRSS2-ERG plays a critical role in cancer progression. |
| 67 | ENSECAT00000004048 | LOC100062761 | 1.15 | - |
| 68 | ENSECAT00000010372 | IRF5 | 1.14 | IRF5 has transcription factors with diverse roles, including virus-mediated activation of interferon, and modulation of cell growth, differentiation, apoptosis, and immune system activity. |
| 69 | ENSECAT00000008287 | LOC100054237 | 1.12 | - |
| 70 | ENSECAT00000021997 | Q8WNS8_HORSE | 1.09 | - |
| 71 | ENSECAT00000013323 | RLF | 1.09 | RLF encodes zinc finger proteins. |
| 72 | ENSECAT00000019618 | LASS2 | 1.05 | This gene encodes a protein that has a sequence similarity to yeast longevity assurance gene 1, which can change the lifespan of yeasts. |
| 73 | ENSECAT00000017881 | RARB | -0.9 | RARB encodes the retinoic acid receptor, beta, which promotes transcription of downstream target genes through recruiting coactivator proteins. |
| 74 | ENSECAT00000025353 | LOC100049936 | -0.9 | - |
| 75 | ENSECAT00000019232 | LOC100055668 | -1.1 | - |
| 76 | ENSECAT00000002811 | LOC100053044 | -1.2 | - |
| 77 | ENSECAT00000016629 | LOC100052677 | -1.2 | - |
| 78 | ENSECAT00000013214 | ZNF212 | -1.3 | This gene belongs to the C2H2-type zinc finger gene family. |
| 79 | ENSECAT00000001233 | LOC100056720 | -1.3 | - |
| 80 | ENSECAT00000016563 | LOC100068226 | -1.3 | - |
| 81 | ENSECAT00000004400 | LOC100066548 | -1.5 | - |
| 82 | ENSECAT00000006708 | LOC100054466 | -1.5 | - |
| 83 | ENSECAT00000026424 | LOC100061258 | -1.6 | - |
| 84 | ENSECAT00000002772 | RFX7 | -1.6 | Winged-helix transcription factor expressed ubiquitously in all tissues examined, especially brain tissue. |
| 85 | ENSECAT00000008599 | LOC100146441 | -1.6 | - |
| 86 | ENSECAT00000026478 | TBX2 | -1.7 | Tbx2 is required for proper optic cup formation and plays a critical early role in regulating regional retinal growth and the acquisition of shape during optic vesicle invagination |
| 87 | ENSECAT00000018038 | ZNF594 | -2 | ZNF594 encodes the zinc-finger transcription factor; however, its function has not been characterized yet. |
| 88 | ENSECAT00000008512 | LOC100146166 | -3 | - |

1. Blood tissue

| **No** | **Transcript name** | **Gene name** | **Average Log2 ratio** | **Description of general functions** |
| --- | --- | --- | --- | --- |
| 1 | ENSECAT00000014856 | LOC100051632 | 2.42 | - |
| 2 | ENSECAT00000025794 | - | 0.99 | - |
| 3 | ENSECAT00000017771 | GATA2 | -1.61 | GATA2 proteins play an essential role in regulating transcription of genes involved in the development and proliferation of hematopoietic and endocrine cell lineages. |

**Supplementary Table S20. RT-PCR primers for seven transcription factors.**

| **Gene name** | **Primer sequence** | | **Product size (bp)** | **TM (℃)** |
| --- | --- | --- | --- | --- |
|  | **(5')-Forward primer-(3')** | **(5')-Reverse primer-(3')** |  |  |
| ENSECAT00000010015 | CACCTCCTGCAAGAACATCC | CAGCCGTACATCTGCTTGAA | 216 | 60 |
| ENSECAT00000011931 | TGGCATTTAGCGAAGTTGTG | CAGAAACCAGATCTGAGGAAAGT | 178 | 59 |
| ENSECAT00000018999 | ACTTCAGCTGCAAGGAGGAG | GTCACTGGTCCTGCTCACCT | 204 | 60 |
| ENSECAT00000021368 | AGTGCATATGGGAGCCAGTC | TGGGCATGGGGTGTTAAATA | 193 | 60 |
| ENSECAT00000021905 | AACGACCACGAGTTCATGC | CTCGGCTCACACTCTGTCCT | 173 | 60 |
| ENSECAT00000025803 | GGAAAAGGAAGCTGGAGAGG | CAACTGCTGGGTTAGCATGA | 176 | 60 |
| ENSECAT00000026478 | AGCACTAGCCTCCTCACCAC | CAGCCCACTCACCAGTCTCT | 198 | 60 |

**Supplementary Table S21**. RT-PCR results of differentially expressed transcription factors in muscle tissue.

| **No** | **Transcript name** | **Gene name** | **Average log2 ratio** | **Individual log2 ratios (GAPDH)** | | | | | |
| --- | --- | --- | --- | --- | --- | --- | --- | --- | --- |
|  |  |  | **DEGs** | **RT-PCR** | | | | | |
| 1 | ENSECAT00000010015 | LOC100073197 | 2.15 | 4.57 | 2.96 | 0.16 | 2.39 | 2.96 | 3.01 |
| 2 | ENSECAT00000011931 | LOC100050849 | 5.62 | 6.63 | 6.57 | 5.05 | 6.90 | 3.65 | 4.10 |
| 3 | ENSECAT00000018999 | LOC100063253 | 2.47 | 3.56 | 3.14 | 2.19 | 1.98 | 2.62 | 4.09 |
| 4 | ENSECAT00000021368 | LOC100069984 | 1.85 | 4.14 | 1.15 | 1.28 | 1.10 | 2.79 | 3.29 |
| 5 | ENSECAT00000021905 | Q865B2_HORSE | 1.92 | 3.66 | 0.92 | 0.68 | 2.65 | 1.98 | 2.51 |
| 6 | ENSECAT00000025803 | LOC100067469 | 2.74 | 1.01 | 3.98 | 1.21 | 1.11 | 1.28 | 3.12 |
| 7 | ENSECAT00000026478 | TBX2 | -1.74 | -3.35 | -0.23 | -1.84 | -2.06 | -0.43 | -4.81 |

**Supplementary Table S22. The list of four genes of which alternative splicing forms showed reversed expression patterns before and after exercising**

| **No** | **Transcript name** | **Genename** | **Tissue** | **Description of general functions** |
| --- | --- | --- | --- | --- |
| 1 | ENSECAG00000011471 | DYNC1 | Muscle | Cytoplasmic dynein 1 light intermediate chain |
| 2 | ENSECAG00000020218 | AXL | Muscle | AXL receptor tyrosine kinase |
| 3 | ENSECAG00000007095 | PLEKHG1 | Muscle | Pleckstrin homology domain containing family G member 1 |
| 4 | ENSECAG00000025107 | COBLL1 | Blood | Cordon-bleu protein-like 1 |

**Supplementary Figure S11. Expression profiles of the genes of which alternative splicing forms showed reversed expression patterns before and after exercising**

**
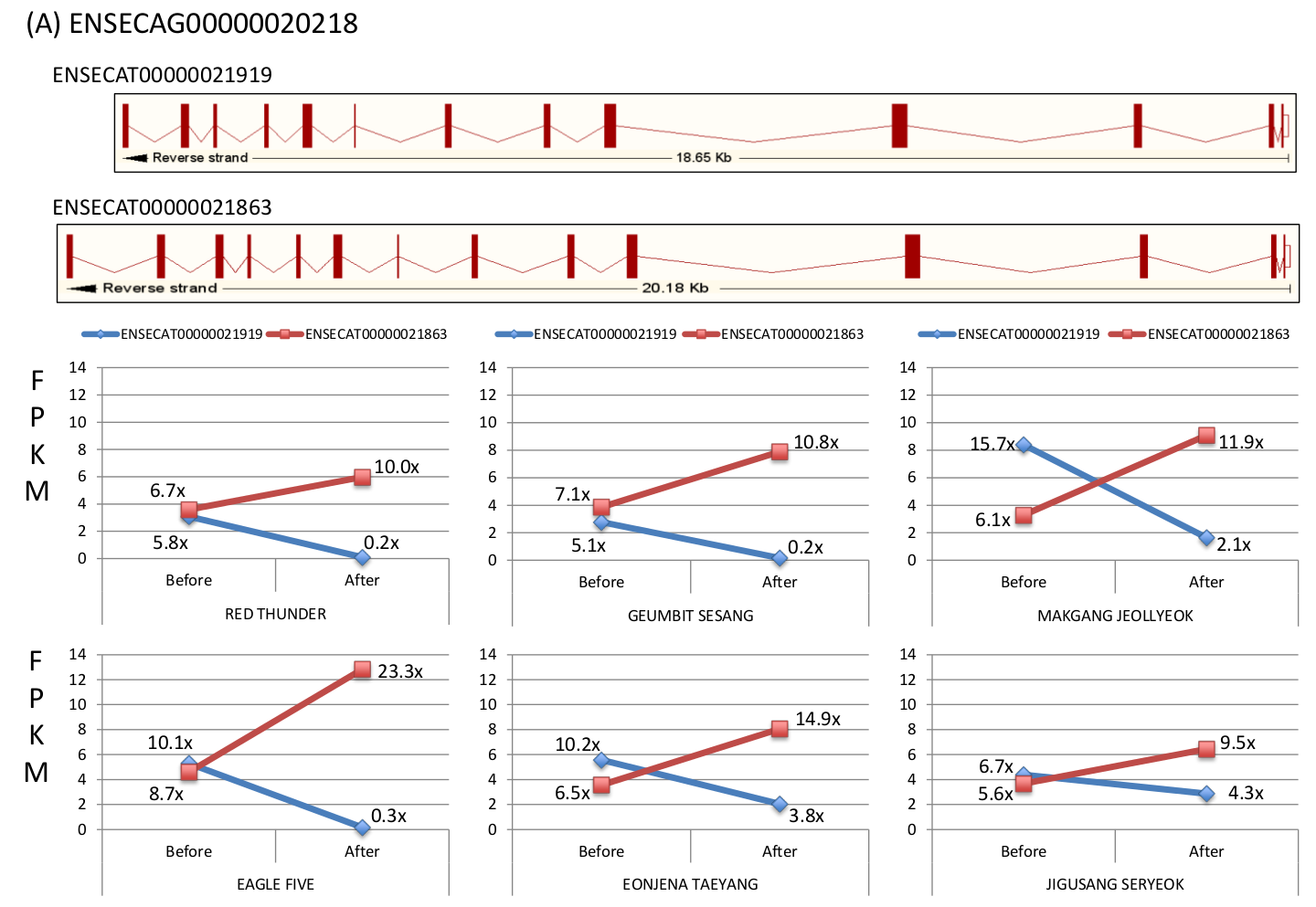
**

**
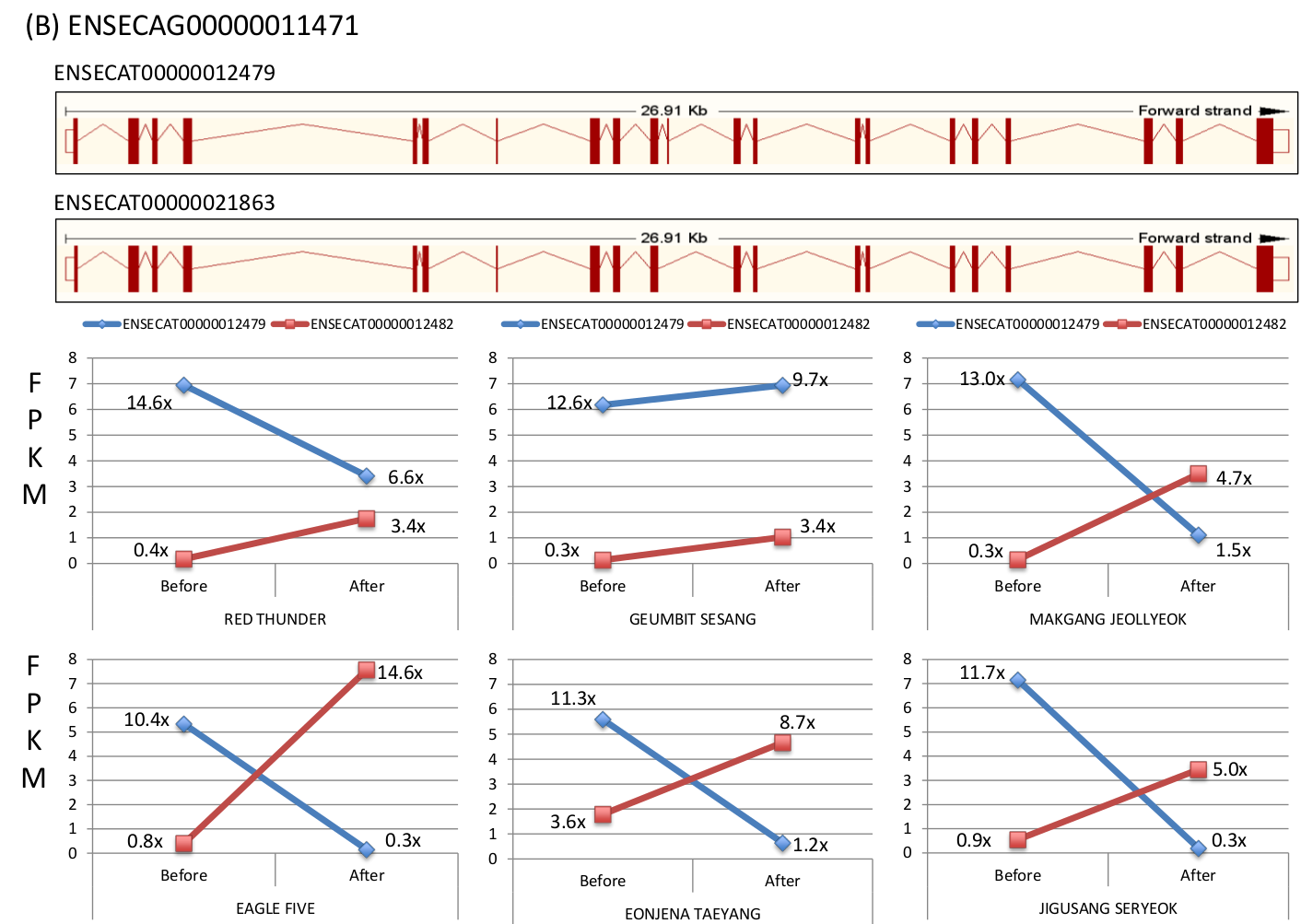
**

**
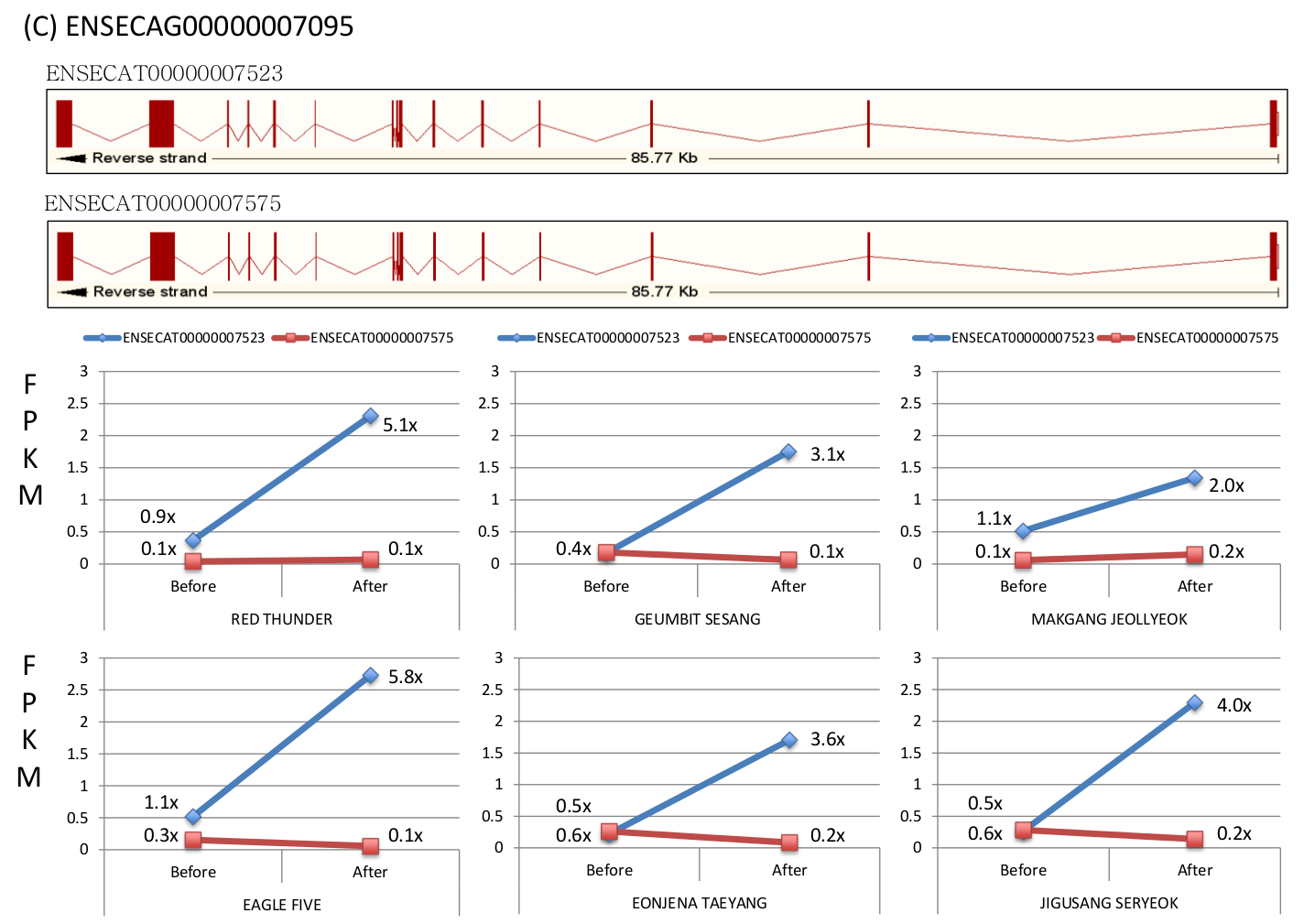
**

**
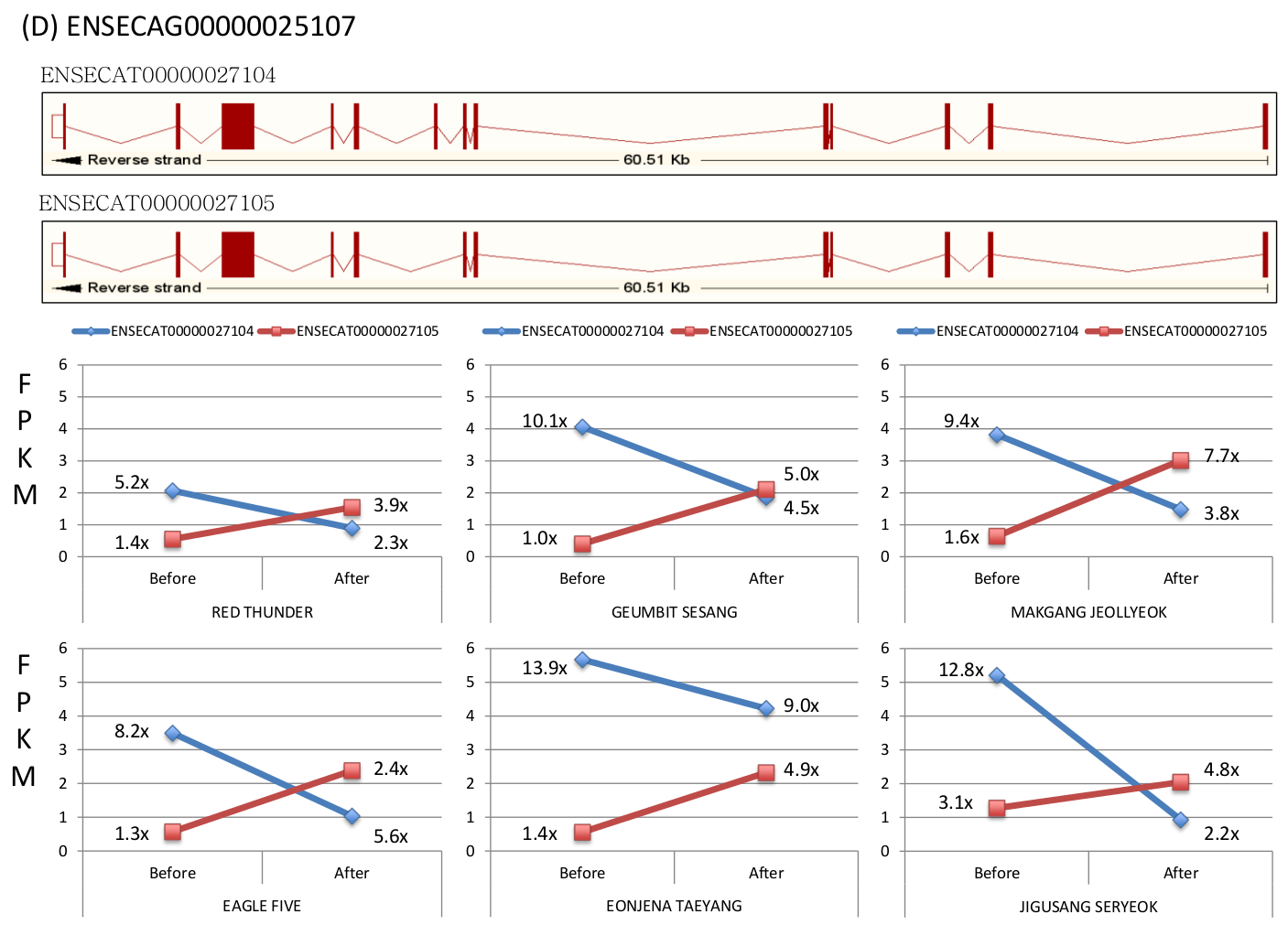
**

**Supplementary Table S23. Number of filtered *de novo* transcripts identified by Cufflink.**

| **Sample name** | **# of valid *de novo* transcripts** | **# of filtered-out *de novo* transcripts** |
| --- | --- | --- |
| BF1B | 24,299 | 49,806 |
| BF1P | 25,972 | 51,520 |
| BF2B | 26,958 | 55,347 |
| BF2P | 25,909 | 56,088 |
| BF3B | 24,021 | 50,018 |
| BF3P | 25,490 | 51,917 |
| BS1B | 27,626 | 57,701 |
| BS1P | 25,130 | 55,535 |
| BS2B | 25,190 | 55,249 |
| BS2P | 25,148 | 49,820 |
| BS3B | 24,606 | 52,429 |
| BS3P | 23,447 | 47,943 |
| MF1B | 20,619 | 30,411 |
| MF1P | 19,404 | 28,962 |
| MF2B | 20,836 | 25,463 |
| MF2P | 19,265 | 25,225 |
| MF3B | 19,184 | 26,227 |
| MF3P | 18,268 | 21,183 |
| MS1B | 21,896 | 29,350 |
| MS1P | 21,739 | 28,176 |
| MS2B | 18,941 | 23,816 |
| MS2P | 18,828 | 26,672 |
| MS3B | 17,770 | 21,332 |
| MS3P | 16,171 | 22,213 |

**Supplementary methods**

Sample preparation of horse muscle and blood samples

The muscle samples of six retired thoroughbred horses were taken from the triceps brachii muscle in the right leg, and the first batch of blood samples was taken from the jugular vein. The second batch of muscle samples was taken from the same location after giving the horses four hours of rest and 30 minutes of trot. Thoroughbred horses usually take 17-18 minutes of canter (lungeing) per day. For our study, we let them have a greater amount of trots (30 minutes) to make the total exercise equivalent to 17-18 minutes of canter.

50 ml of blood was taken from each horse from the carotid artery using a syringe. It was divided into smaller quantity (4 ml) tubes of different types (Citrate, EDTA, and SST). The tubes were stored at 4℃ after mixing sufficiently with anticoagulants.

RNA Seq Library preparation and sequencing

Total RNAs from muscle and blood were prepared using TRIzol (Invitrogen) and a RNeasy RNA Purification Kit with DNase treatment (Qiagen), according to the instruction manual. One microliter of cleaned total RNA was consumed to check RNA quality using BioAnalyzer 2011 with an RNA chip (RIN > 7 and 28S:18S ratio > 1.0). Messenger RNA sequencing libraries were generated using an mRNA Seq Sample Prep Kit following the manufacturer’s manual (Illumina). 5 ug of poly(A) mRNA was isolated from the total RNA using oligo-dT beads. The mRNA was then fragmented and randomly primed for reverse transcription, followed by the second-strand synthesis to create double-stranded cDNA fragments. The double-stranded short cDNA fragments were purified with a QiaQuick PCR extraction kit (Qiagen) and resuspended in an EB buffer. Each end of cDNA was repaired with a combination of fill-in reactions and blunted by exonuclease. The addition of A-base was applied to the blunt end DNA followed by sequencing adaptor ligation. The required fragments were purified by agarose gel electrophoresis and a QIAquick Gel Extraction Kit (Qiagen), and enriched by PCR amplification. The library quantity and quality were tested using Qubit (BioRad) and BioAnalyzer 2100, followed by qRT-PCR for accurate quantification. The sequencing of the library was performed using an Illumina HiSeq2000 machine as 90 pair-end reads (Supplementary Table S2).

Alignment of 1.3 billion reads against the horse reference genome

We performed the alignment of all RNA-Seq raw sequences against the Ensembl genome (Release 62) using TopHat 1.2.0 with two options, --mate-inner-dist=200, --allow-indels for paired-end sequences . To increase the performance of the alignment process, we used cluster computers with the SGE (Sun Grid Engine 6.2; <http://wikis.sun.com/display/GridEngine/Home/>), and all processes were finished within one day with 96 CPU cores.

The procedure of selecting the exons identified by the Cufflink without a gene model

Usually, unigene sequences should contain the translated regions with sufficiently long lengths. Assuming that most of unigene sequences obtained from this study were not full-length cDNA sequences, the state machine to select a series of exons was designed with two main conditions: i) In the case of unigene clusters containing one exon, the unigene sequences of which 40% region was translated well were selected, and ii) In the case of multiple exons, once the translated region in a certain exon was found, it should at least continue to the next exons.

Identification of unigene clusters with the current gene model as well as *de novo* exon structures provided by Cufflink

To identify novel genes not predicted by the gene prediction software, we utilized the results generated by Cufflink without genome annotation data. From the results of 24 samples, we attempted to cluster the novel genes based on the genomic coordination to define unigene clusters (UCs). The generated UCs were subjected to the filter which extracted UCs overlapping with the genome annotation. The expressed genes annotated by the pipeline and the filtered UCs were merged as the final set of UCs.

Assembly of the unmapped sequences with the SOAPdenovo

From the BAM files generated by TOPHAT, a Perl script extracted two categories of raw reads: i) raw reads of which both pair-end sequences were not matched on the reference genome, and ii) raw reads of which one of the pair-end sequences was not mapped on the reference genome. To find an optimized k-mer value for these unmapped sequences, we tested from k=17 to k=25, and k=21 showed the best results in *de novo* assembly (Supplementary Table S6). With k=21, we assembled 24 sets of the unassembled sequences (Supplementary Table S7).

Calculating the total span of the unigenes identified in this study considering alternative splicing forms

Because Cufflink provided alternative splicing forms, we first removed redundant exons according to the genomic coordinate. After that, the total span of the unigenes was calculated from the non-redundant exons.

Generating unigene sequences from the unigene clusters with merging alternative splicing forms from 24 samples

A unigene cluster is a set of alternative splicing forms identified from 24 samples. We removed the redundancy of the alternative splicing forms in each unigene cluster. Based on the non-redundant alternative splicing forms with the horse reference genome, sequences were generated by considering the single nucleotide variations identified by an in-house bioinformatics pipeline.

Filtering single nucleotide variations and small InDels from the alignment results of TopHat

We discovered SNPs and INDELs from the alignment results of TopHap 1.2.0 . Using samtools with a pileup option, we could find all the bases mapped to each genomic position. From the alignments, we detected candidate SNPs or INDELs containing alternative mapping to the reference. Next, we applied the serial filters to the candidate SNVs to get reliable SNVs only (Figure S5 and Table S11). INDELs were restricted with at least ten reads mapped to the position and five reads with alternative mapping. The alternative reads had to occupy at least 30% of the total reads mapped. For SNPs, we used criteria based on the parameters of mapping: # of total reads mapped to the position >10; # of alternative reads mapped to the position >5; # of alternative read / # of total read >30%; the average of mapping quality of alternative reads >30; and average alignment quality of reads >110. SNPs were, then, restricted to positions in an exon area identified by Cufflink 0.9.3 . We observed that the bases on read ends at near INDELs may be misaligned due to the limitation of the alignment algorithm, which is known to produce a great number of false-positive SNPs (the exon-intron boundary mis-alignment filter). To avoid this problem, we discarded the SNPs with over 30% of alternative bases located on read ends and SNPs located closer than five bp to INDELs.

Checking the open reading frame of the final unigene sequences considering exon structure

To remove false-positive messenger RNAs from RNA-Seq data, the unigene sequences generated by considering single nucleotide variations were investigated, whether they had proper open reading frame or not. Exon structures of all unigenes predicted by Cufflink were also considered. The largest and the second largest amino acids translated with six frames were checked in order to identify: i) whether the translated amino acid occupied more than 50% of the unigene length if the unigene has only one exon, or ii) whether the translated amino acid occupied more than 80% of the unigene length if the unigene had multiple exons.

Gene Ontology analysis of four RNA-Seq datasets

To calculate the distribution of gene ontology terms of two previous datasets of RNA-Seq, mouse muscle dataset (SRA accession is SRP000198) and human muscle RNA-seq data from the Illumina BodyMap2 project (SRA accession is ERR030899), we used TopHat 1.2.0 with default options for mapping raw sequences against reference genomes, *Mus musculus* and *Homo sapiens*, provided by Ensembl (<http://www.ensembl.org/>; release 62). After getting mapped genes from the datasets, a BLAST search for all genes (e-value cutoff: 1e-3) was conducted against non-redundant datasets. As the next step, the homolog proteins were assigned based on the Gene Ontology (GO) terms provided by the NCBI . When categorizing the proteins, the third depth of the GO tree (May 25, 2011) was considered.

Identification of differentially expressed genes between two conditions

To identify the genes that are differently expressed by exercise, we selected the genes that showed more than 2x up- or 2x down-regulation in at least four horses.

Identification of transcription factors from differentially expressed genes

To select transcription factors, the predicted horse transcription factors from the DBD ([http://dbd.mrc-lmb.cam.ac.uk](http://dbd.mrc-lmb.cam.ac.uk/)/) were downloaded and matched to the list of DEGs we identified using the Ensembl transcript names.

Reverse transcript PCR for confirming novel transcripts

To confirm the novel transcripts, reverse transcript PCR (RT-PCR) was performed on a Mastercycler Pro-S (Eppendorf) using TaKaRa Taq^TM^ Hot Start Version (TaKaRa). cDNA per reaction was prepared from 1 ul of four individuals’ horse blood. Primer sequences for the novel transcripts were designed using Primer3 software (<http://frodo.wi.mit.edu/primer3/>) . Additional information, such as melting temperature (TM) and product size of each primer, is shown in Supplementary Table S4. The RT-PCR conditions were as follows: an initial step of 94℃ for 4 min, 30 cycles of 94℃ for 40 sec, TM ℃ for each primer for 1 min, and 72℃ for 1 min, and the last 72℃ elongation for 7 min. The RT-PCR products were separated on electrophoresis on a 1.2% agarose gel with marker sequences.

Quantitative Reverse Transcript PCR

Primers for seven transcription factors (Supplementary Table S20) were designed using Primer3 software (<http://frodo.wi.mit.edu/primer3/>) . SYBR green quantitative real-time reverse transcript-PCR (qRT-PCR) was performed on a Rotor Gene 3000 (Corbett Research) using 1 ul of cDNA template and 10 ul QuantiTect® SYBR® Green PCR Master Mix (Qiagen), adjusted to 20 ul with water and primer solutions, per reaction. The quantitative real-time RT-PCR amplification was carried out for 50 cycles of 94℃ for 10 sec, 58℃ for 15 sec, and 72℃ for 15 sec. A melting curve was produced by varying the temperature from 55℃ to 99℃ over 30 sec while data was collected by Rotor-gene 3000 software version 6.0.19 (Corbett Research). Independent experiments were performed in triplicate. GAPDH mRNAs were used to normalize the results of the qRT-PCR. Amplification specificity was tested through melting curve analysis. Non-specific PCR products were not detected for either primer pair. As a reference, the *GAPDH* gene was amplified with a forward primer 5’-ATCTGACCTGCCGCCTGGAG-3’ and a reverse primer 5’- CGATGCCTGCTTCACCACCTTC -3’ (GenBank accession no. AF157126).

**References**

1. Trapnell C, Williams BA, Pertea G, Mortazavi A, Kwan G, van Baren MJ, Salzberg SL, Wold BJ, Pachter L: **Transcript assembly and quantification by RNA-Seq reveals unannotated transcripts and isoform switching during cell differentiation**. *Nat Biotechnol* 2010, **28**(5):511-515.

2. Gregg C, Zhang J, Weissbourd B, Luo S, Schroth GP, Haig D, Dulac C: **High-resolution analysis of parent-of-origin allelic expression in the mouse brain**. *Science* 2010, **329**(5992):643-648.

3. Wade CM, Giulotto E, Sigurdsson S, Zoli M, Gnerre S, Imsland F, Lear TL, Adelson DL, Bailey E, Bellone RR *et al*: **Genome sequence, comparative analysis, and population genetics of the domestic horse**. *Science* 2009, **326**(5954):865-867.

4. Schroder W, Klostermann A, Distl O: **Candidate genes for physical performance in the horse**. *Vet J* 2010.

5. Trapnell C, Pachter L, Salzberg SL: **TopHat: discovering splice junctions with RNA-Seq**. *Bioinformatics* 2009, **25**(9):1105-1111.

6. Li R, Yu C, Li Y, Lam TW, Yiu SM, Kristiansen K, Wang J: **SOAP2: an improved ultrafast tool for short read alignment**. *Bioinformatics* 2009, **25**(15):1966-1967.

7. Mortazavi A, Williams BA, McCue K, Schaeffer L, Wold B: **Mapping and quantifying mammalian transcriptomes by RNA-Seq**. *Nat Methods* 2008, **5**(7):621-628.

8. Ashburner M, Ball CA, Blake JA, Botstein D, Butler H, Cherry JM, Davis AP, Dolinski K, Dwight SS, Eppig JT *et al*: **Gene ontology: tool for the unification of biology. The Gene Ontology Consortium**. *Nat Genet* 2000, **25**(1):25-29.

9. Wilson D, Charoensawan V, Kummerfeld SK, Teichmann SA: **DBD--taxonomically broad transcription factor predictions: new content and functionality**. *Nucleic Acids Res* 2008, **36**(Database issue):D88-92.

10. Rozen S, Skaletsky H: **Primer3 on the WWW for general users and for biologist programmers**. *Methods Mol Biol* 2000, **132**:365-386.
